# Safety and antitumor activity of the anti–PD-1 antibody pembrolizumab in patients with advanced colorectal carcinoma

**DOI:** 10.1371/journal.pone.0189848

**Published:** 2017-12-28

**Authors:** Bert H. O’Neil, John M. Wallmark, David Lorente, Elena Elez, Judith Raimbourg, Carlos Gomez-Roca, Samuel Ejadi, Sarina A. Piha-Paul, Mark N. Stein, Albiruni R. Abdul Razak, Katia Dotti, Armando Santoro, Roger B. Cohen, Marlena Gould, Sanatan Saraf, Karen Stein, Sae-Won Han

**Affiliations:** 1 Department of Hematology/Oncology, Indiana University Simon Cancer Center, Indianapolis, Indiana, United States of America; 2 Department of Hematology/Oncology, Maryland Hematology Oncology, Rockville, Maryland, United States of America; 3 Prostate Cancer Targeted Therapy Group, The Royal Marsden, Sutton, United Kingdom; 4 Vall d’Hebron Institute of Oncology, Vall d'Hebron University Hospital, Barcelona, Spain; 5 Department of Oncology Medical, Institut de Cancérologie de l’Ouest, Nantes, France; 6 Department of Oncology Medical, Institut Claudius Regaud and IUCT-Oncopole, Toulouse, France; 7 Department of Oncology, Scottsdale Healthcare, Virginia G. Piper Cancer Center, Scottsdale, Arizona, United States of America; 8 Department of Investigational Clinical Therapeutics, The University of Texas MD Anderson Cancer Center, Houston, Texas, United States of America; 9 Department of Medical Oncology, Rutgers Cancer Institute of New Jersey, New Brunswick, New Jersey, United States of America; 10 Clinical Cancer Research Unit, Princess Margaret Cancer Centre, Toronto, Canada; 11 Department of Medical Oncology, Fondazione I.R.C.C.S. Istituto Nazionale dei Tumori, Milan, Italy; 12 Humanitas Cancer Center, Humanitas Research Hospital, Rozzano, Italy; 13 Division of Hematology-Oncology, University of Pennsylvania, Philadelphia, Pennsylvania, United States of America; 14 Department of Clinical Research, Merck & Co., Inc., Kenilworth, New Jersey, United States of America; 15 Division of Hematology and Medical Oncology, Seoul National University Hospital, Seoul, Republic of Korea; Emory University Winship Cancer Institute, UNITED STATES

## Abstract

**Background:**

Colorectal cancers (CRCs) expressing programmed death ligand 1 (PD-L1) have poor prognosis. In the multicohort KEYNOTE-028 trial, the anti–PD-1 antibody pembrolizumab was evaluated in 20 PD-L1–positive advanced solid tumors. Herein, we report results for the advanced CRC cohort.

**Methods:**

Patients with advanced, treatment-resistant PD-L1–positive carcinoma of the colon or rectum were enrolled, regardless of microsatellite instability (MSI) status. Pembrolizumab 10 mg/kg was administered every 2 weeks for up to 2 years or until disease progression/unacceptable toxicity. Response was assessed every 8 weeks for the first 6 months and every 12 weeks thereafter. Primary end points were safety and overall response rate by investigator review per Response Evaluation Criteria in Solid Tumors version 1.1. Data cutoff was June 20, 2016.

**Results:**

Of 137 patients with CRC and samples evaluable for PD-L1 expression, 33 (24%) had PD-L1–positive tumors, of which 23 were enrolled. Median follow-up was 5.3 months, and 8 patients (35%) reported treatment-related adverse events (AEs), most commonly fatigue (n = 3, 13%), stomatitis (n = 2, 9%), and asthenia (n = 2, 9%). One patient (4%) experienced grade 4 treatment-related increased blood bilirubin. No grade 3 AEs, discontinuations, or deaths were attributed to treatment. Most patients (n = 15, 65%) experienced progressive disease. One partial response occurred in a patient (4%) with MSI-high CRC.

**Conclusion:**

Pembrolizumab demonstrated a favorable safety profile in advanced PD-L1–positive CRC. Antitumor activity was observed in a single patient with MSI-high CRC, warranting further evaluation in this patient population. (Clinicaltrials.gov registration: NCT02054806)

## Introduction

Colorectal cancer (CRC) is the third most commonly diagnosed cancer in the United States and the third leading cause of cancer-related deaths in men and women [1,2]. Worldwide, almost 700,000 people died as a result of CRC in 2012 [3]. The occurrence of CRC and CRC-related mortality rate increases with age; median age at diagnosis is 68 years, and 93% of deaths occur in people aged ≥50 years [1,4]. The relative 5-and 10-year survival rates for CRC after diagnosis are 65% and 58%, respectively [1]. Survival is strongly associated with stage at diagnosis; most cases are diagnosed at later stages, and the 5-year survival rate is only 13% for those with distant metastases [1].

CRC is a heterogeneous disease driven in part by loss of genomic stability. Molecular phenotypes of CRC are defined by the mutational status of genes encoding mismatch repair (MMR) proteins (encoded by *MLH1*, *MSH2*, *MSH6*, or *PMS2*), RAS (encoded by *KRAS*), and RAF (encoded by *BRAF*) [5]. MMR deficiency, which results in microsatellite instability (MSI), occurs across the CRC genome and reflects genetic dysfunction, whereas *KRAS* and *BRAF* mutations are thought to drive disease development [5]. High levels of MSI are found in approximately 15% of CRC tumors (known as MSI-high [MSI-H] tumors) and are generally attributable to either epigenetic silencing or germline mutations in *MMR* genes [5]. MSI status is generally thought of as a positive prognostic marker for early-stage CRC; overall survival (OS) is superior for patients with early disease and the MSI-H subtype compared with those with microsatellite-stable (MSS) disease [5]. However, for patients with metastatic CRC, MSI status is considered a negative prognostic marker.

The interaction between the programmed death 1 (PD-1) receptor with its ligands, PD-L1 and PD-L2, normally functions as an immune checkpoint that regulates the balance between T-cell activation, immune tolerance, and immune-related tissue damage, and is a pathway hijacked by tumors to evade immune surveillance [6–8]. PD-1 is expressed on T, B, and natural killer T cells, as well as activated monocytes and a large proportion of tumor-infiltrating lymphocytes (TILs) in various tumors [6,8]. Binding of PD-L1 (expressed on cells of multiple lineages) or PD-L2 (expressed on macrophages and dendritic cells) to the PD-1 receptor ultimately inhibits T-cell function [9].

Both PD-1 ligands can be constitutively expressed or induced in a variety of cell types, including tumor cells [7,8]. PD-L1 overexpression by tumors (eg, in breast, lung, melanoma, liver, head and neck, and colon tumors) might enable tumor cells to block antitumor immune responses, and is associated with poor prognosis [6,8,9]. Expression of PD-1 pathway components on tumor cells and evidence that they play a critical role in tumor immune evasion renders this pathway an attractive target for therapeutic intervention. In CRC tumors, PD-L1 expression is predominantly associated with TILs, with limited PD-L1 expression on the tumor cells themselves [10].

Pembrolizumab is a highly selective humanized immunoglobulin G4/κ monoclonal antibody designed to directly block the PD-1:PD-L1/PD-L2 interaction by binding to PD-1. Pembrolizumab has demonstrated robust antitumor activity and a favorable safety profile in multiple tumor types, and is currently approved in more than 60 countries for one or more advanced malignancies.

An association between therapeutic response to PD-L1 blockade and pretreatment tumor PD-L1 expression has been reported [11]. However, therapeutic responses have been observed in patients with PD-L1–negative tumors, and the prognostic/predictive utility of tumor PD-L1 expression has yet to be validated because such expression is heterogeneous and may be affected by prior therapies [12]. Herein, we report the safety and antitumor activity of pembrolizumab in a cohort of patients with advanced PD-L1–positive CRC (both MSI and MSS) enrolled in the phase Ib multicohort KEYNOTE-028 trial (ClinicalTrials.gov identifier: NCT02054806).

## Patients and methods

### Study design and patients

KEYNOTE-028 was an international, multicenter, open-label, nonrandomized, single-arm phase Ib trial that was designed to assess the safety of pembrolizumab and to explore whether pembrolizumab showed antitumor activity in 20 different cohorts of patients with advanced solid tumors considered to have significant unmet medical need. All patients were screened prior to enrollment for PD-L1 positivity (as defined in the Treatment and Assessments section). Herein, we report results for the cohort of patients with advanced colon or rectal adenocarcinoma. This study was conducted at 15 investigational sites in Canada, France, Italy, the Republic of Korea, Spain, the United Kingdom, and the United States.

Patient eligibility criteria included age ≥18 years, measurable disease at baseline based on Response Evaluation Criteria in Solid Tumors (RECIST) version 1.1, Eastern Cooperative Oncology Group performance status of 0 or 1, and adequate organ function (hematologic, renal, hepatic, and coagulation) as determined by laboratory testing within 10 days of the first pembrolizumab dose. For the CRC cohort, all patients had tumor samples assessed for MMR proficiency, and eligible patients must have had PD-L1–positive, histologically or cytologically confirmed, locally advanced, or metastatic colon or rectal adenocarcinoma for which prior standard therapy was ineffective or for which standard therapy did not exist or was not considered appropriate. The procedure for MMR testing was not specified in the study protocol, and therefore, MMR status was retrospectively determined by the investigator.

Exclusion criteria included prior anticancer monoclonal antibody therapy within the 4 weeks preceding the first pembrolizumab dose; chemotherapy, targeted small-molecule therapy, or radiation therapy within the 2 weeks preceding the first pembrolizumab dose; diagnosis of immunodeficiency or need for systemic steroid therapy within the 7 days preceding the first pembrolizumab dose; prior therapy with antibodies against PD-1, PD-L1, or any other immune-checkpoint inhibitor; active autoimmune disease; interstitial lung disease; active infection necessitating systemic therapy; and active brain metastases.

### Treatment and assessments

Patients received pembrolizumab intravenously at a dose of 10 mg/kg once every 2 weeks (Q2W) for 24 months or until confirmed disease progression, unacceptable adverse events (AEs), withdrawal of consent, or investigator decision to discontinue pembrolizumab. Response was assessed by computed tomography (CT) or magnetic resonance imaging (MRI) every 8 weeks for the first 6 months, and every 12 weeks thereafter. AEs were monitored throughout the study and for 30 days after treatment discontinuation and were graded according to the National Cancer Institute Common Terminology Criteria for Adverse Events, version 4.0. Information on serious AEs was collected for 90 days after the end of treatment. AEs of special interest were defined as events with potentially drug-related immunologic causes that were consistent with an immune phenomenon, regardless of attribution to treatment or immune relatedness by the investigator.

An archived formalin-fixed, paraffin-embedded tumor sample or a newly obtained biopsy specimen was assessed at a central laboratory for PD-L1 expression at screening with a laboratory-developed prototype immunohistochemistry (IHC) assay (QualTek Molecular Laboratories, Goleta, CA, USA) [13] using the 22C3 antibody (Merck & Co., Inc., Kenilworth, NJ, USA). PD-L1 positivity was defined as membrane staining in ≥1% of scorable cells or the presence of a distinctive interface pattern in neoplastic cells and contiguous mononuclear inflammatory cells [13].

### End points

Primary end points were safety and overall response rate (ORR) by investigator review. ORR was defined as the proportion of patients experiencing complete response or partial response per RECIST v1.1 at any time during the study. A confirmation assessment of ORR was required per RECIST v1.1. Secondary end points were progression-free survival (PFS), defined as time from enrollment to the first documented instance of disease progression according to RECIST v1.1 or death from any cause, whichever occurred first; OS, defined as time from enrollment to death from any cause; and duration of response (DOR), defined as time from first RECIST v1.1–based response to disease progression in patients who experienced partial response or better.

### Study oversight

The study protocol (number MK-3475-028-02; S1 Protocol) and all amendments were approved by the appropriate institutional review boards and ethics committees at each participating institution. The study was conducted in accordance with the protocol, Good Clinical Practice guidelines, and the ethical principles outlined in the Declaration of Helsinki. All patients provided written informed consent.

### Statistical analyses

Per protocol, multiple interim analyses could be performed because of the sequential design of the study. A sequential monitoring procedure was used to evaluate efficacy and futility after ≥6 patients in a specific cohort had ≥1 postbaseline response assessment. Enrollment continued provided ≥1 of the first 6 patients responded. Using the sequential probability ratio test (SPRT) method in the open-source R software environment (available at https://www.r-project.org/), a sample size of 22 evaluable patients per cohort was calculated to provide 80% power to demonstrate that the best ORR exceeded 10% at an overall one-sided 8% alpha-level if the true best ORR was 35%. An ORR of 35% was considered to be clinically meaningful for each of the 20 tumor types investigated in the study. The efficacy analysis population included all patients who received ≥1 dose of pembrolizumab and had measurable disease at baseline. The safety analysis population included all patients who received ≥1 dose of pembrolizumab. The truncated sequential probability test was used to evaluate ORR, whereas PFS, OS, and DOR were analyzed using the Kaplan-Meier method. All statistical tests were conducted at the one-sided alpha level of 0.025 using SAS, version 9.3 (SAS Institute, Cary, NC, USA). The data cutoff for this analysis was June 20, 2016.

## Results

### Patient baseline characteristics

**Fig 1:**
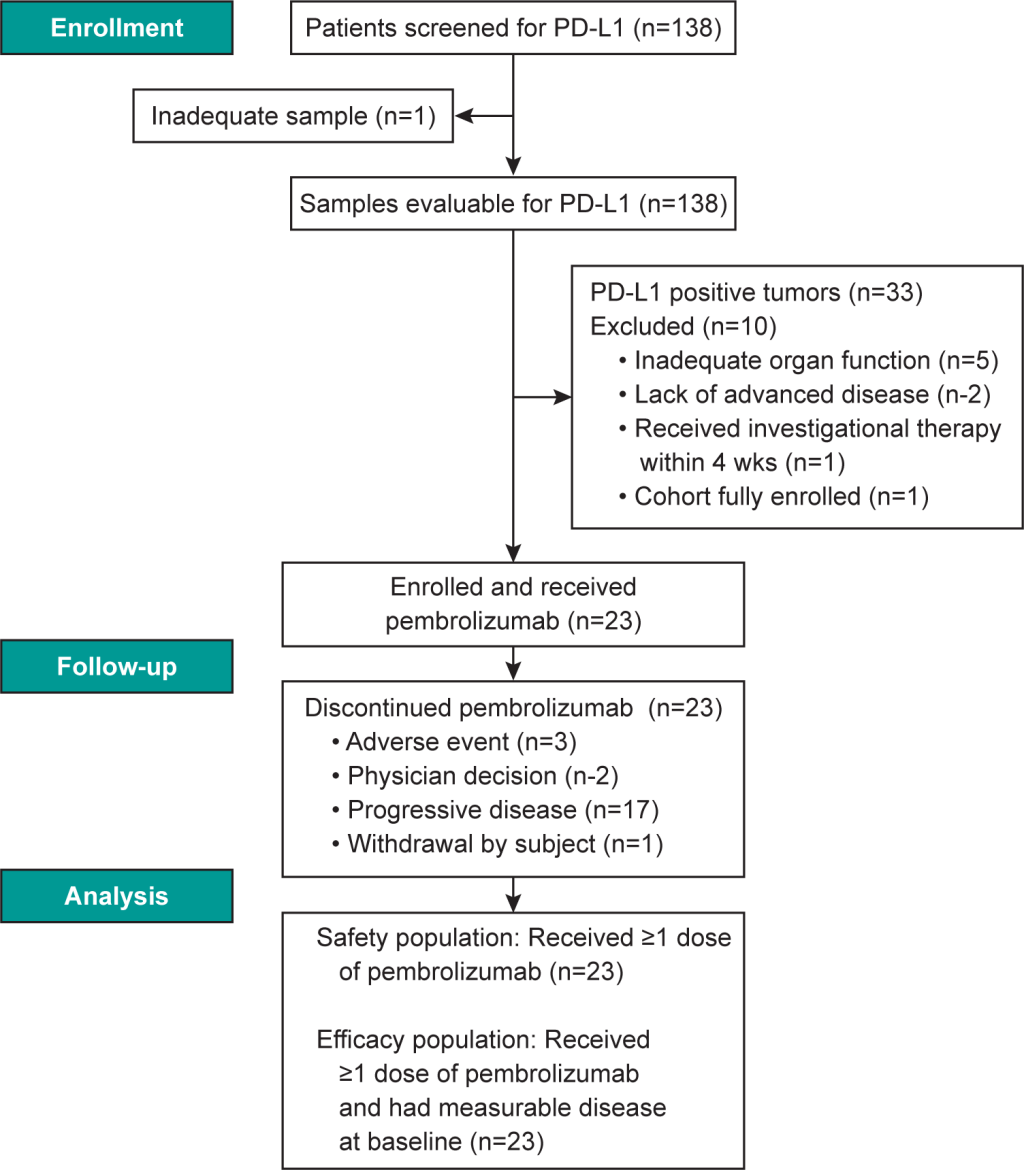
CONSORT diagram.

A total of 138 patients with CRC were screened for tumor PD-L1 expression (Fig 1). Adequate tumor samples for evaluation of PD-L1 expression were available for 137 of these patients, 33 (24%) of which had PD-L1‒positive tumors. Ten of these 33 patients were excluded from enrollment because of inadequate organ function (*n* = 5), lack of advanced disease (*n* = 2), having received investigational therapy within 4 weeks preceding the first pembrolizumab dose (*n* = 1), withdrawal of consent (*n* = 1), or cohort enrollment quota having been achieved (*n* = 1). The 23 remaining patients were enrolled in the study between March and June 2014. Median age was 57 years (range, 40–78 years) and 13 patients (57%) were men (**Table 1**). The majority of patients had received previous treatment for advanced disease, with 15 (65%) having received ≥ 3 prior therapies. One patient (4%) had MSI-H CRC.

**Table 1:** Baseline demographics and clinical characteristics.

Characteristic	*N* = 23
Median age, years (range)	57 (40–78)
Sex, *n* (%)	
Male	13 (57)
Female	10 (43)
Race, *n* (%)	
White	11 (48)
Asian	6 (26)
Black or African American	2 (9)
Not specified	4 (17)
ECOG performance status, *n* (%)	
0	6 (26)
1	16 (70)
Unknown	1 (4)
MMR mutational status, *n* (%)	
MSS	22 (96)
MSI-H	1 (4)
Tumor histology, *n* (%)	
Adenocarcinoma	22 (96)
Lieberkuhn adenocarcinoma	1 (4)
Tumor location, *n* (%)	
Colon	16 (70)
Rectum	5 (22)
Cecum	1 (4)
Colon and rectum	1 (4)
Prior adjuvant or neoadjuvant systemic therapy, *n* (%)	11 (48)
Prior lines of therapy for advanced disease, *n* (%)	
0	1 (4)
2	7 (30)
3	7 (30)
4	5 (22)
≥5	3 (13)
Categories of prior therapy for early or advanced disease,* *n* (%)	
Chemotherapy	23 (100)
Monoclonal antibody	18 (78)
Antibody therapy	5 (22)
Investigational therapy	2 (9)
Hormonal therapy	1 (4)
Immunomodulatory therapy	1 (4)
Unknown	2 (9)

Abbreviations: ECOG, Eastern Cooperative Group Oncology Status; MMR, mismatch repair; MSI-H, microsatellite instability-high; MSS, microsatellite-stable.

*Patients may have received ≥1 category of prior therapy.

### Safety

The median follow-up duration as of the data cutoff date was 5.3 months (range, 1.0–26.2 months). Treatment-related AEs were reported for 8 patients (35%). Events that occurred in more than one patient were fatigue (*n* = 3, 13%), stomatitis (*n* = 2, 9%), and asthenia (*n* = 2, 9%) (**Table 2**). There were no grade 3 treatment-related AEs. One patient (4%) experienced grade 4 treatment-related increased blood bilirubin level (**Table 2**). One patient (4%) experienced an AE of likely immune etiology: grade 2 hyperthyroidism. There were no AE-related study discontinuations or deaths attributable to treatment.

**Table 2:** Treatment-related adverse events.

Treatment-related adverse events *N* = 23	Grade 1 or 2 *n* (%)	Grade 3 *n* (%)	Grade 4 *n* (%)
Fatigue	3 (13)	0	0
Asthenia	2 (9)	0	0
Stomatitis	2 (9)	0	0
Arthralgia	1 (4)	0	0
Constipation	1 (4)	0	0
Decreased appetite	1 (4)	0	0
Diarrhea	1 (4)	0	0
Erythema	1 (4)	0	0
Flatulence	1 (4)	0	0
Myalgia	1 (4)	0	0
Nausea	1 (4)	0	0
Pruritus	1 (4)	0	0
Vomiting	1 (4)	0	0
Increased blood bilirubin	0	0	1 (4)

Adverse events were graded according to the National Cancer Institute Common Terminology Criteria for Adverse Events, version 4.0, where grade 1 = mild, with asymptomatic or mild symptoms or clinical or diagnostic observations only, with intervention not indicated; grade 2 = moderate, with minimal, local, or noninvasive intervention indicated or limiting of age-appropriate instrumental activities of daily living; grade 3 = severe or medically significant but not immediately life threatening or hospitalization or prolongation of hospitalization indicated or disabling or limiting of self-care activities of daily living; grade 4 = life-threatening consequences or urgent intervention indicated; and grade 5 = leads to death.

### Antitumor activity

**Fig 2:**
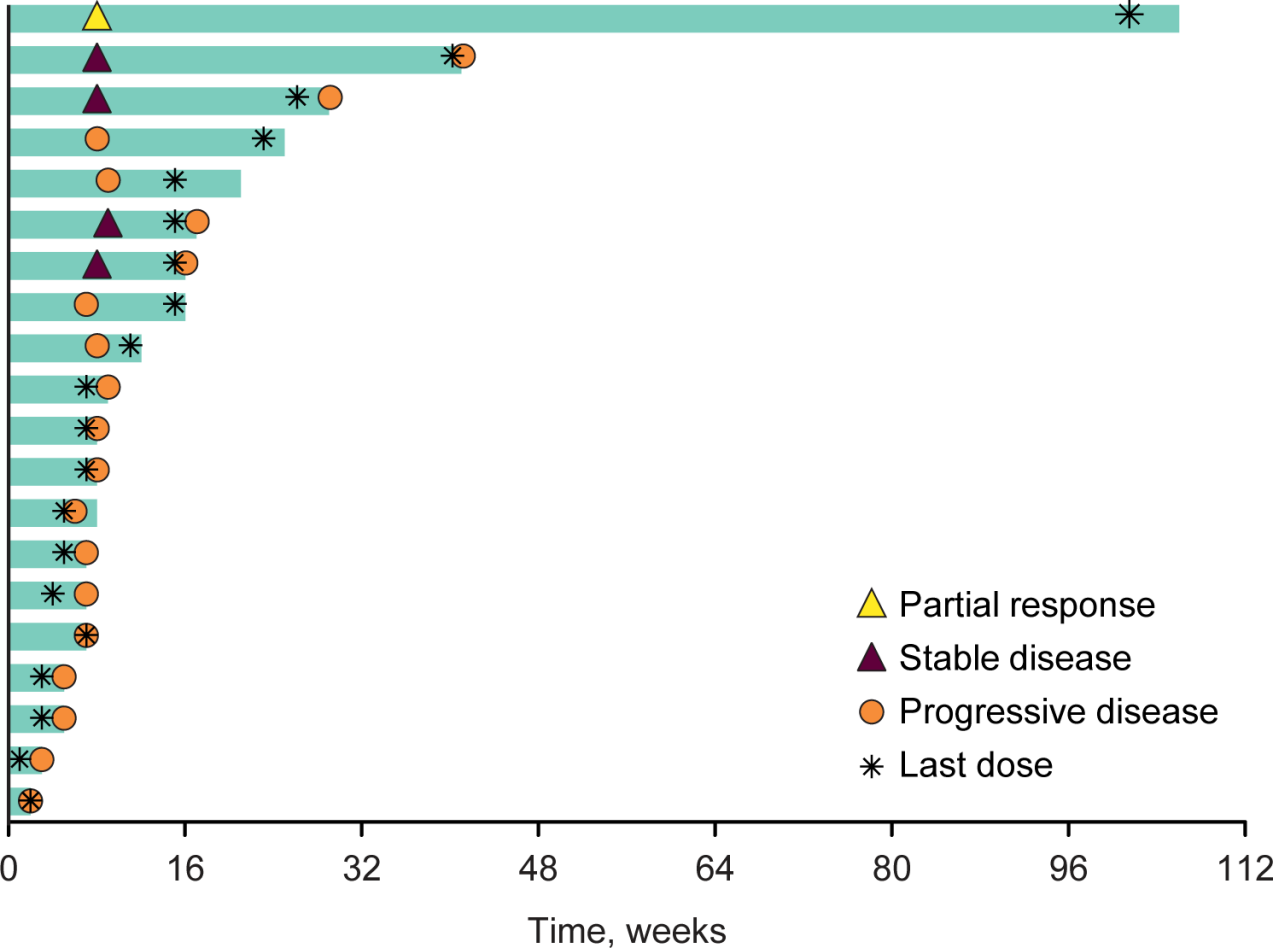
Treatment exposure and response duration. Includes patients evaluable for best overall response per Response Evaluation Criteria in Solid Tumors version 1.1 by investigator review (*n* = 20). The length of each bar represents the time to the last imaging assessment.

**Fig 3:**
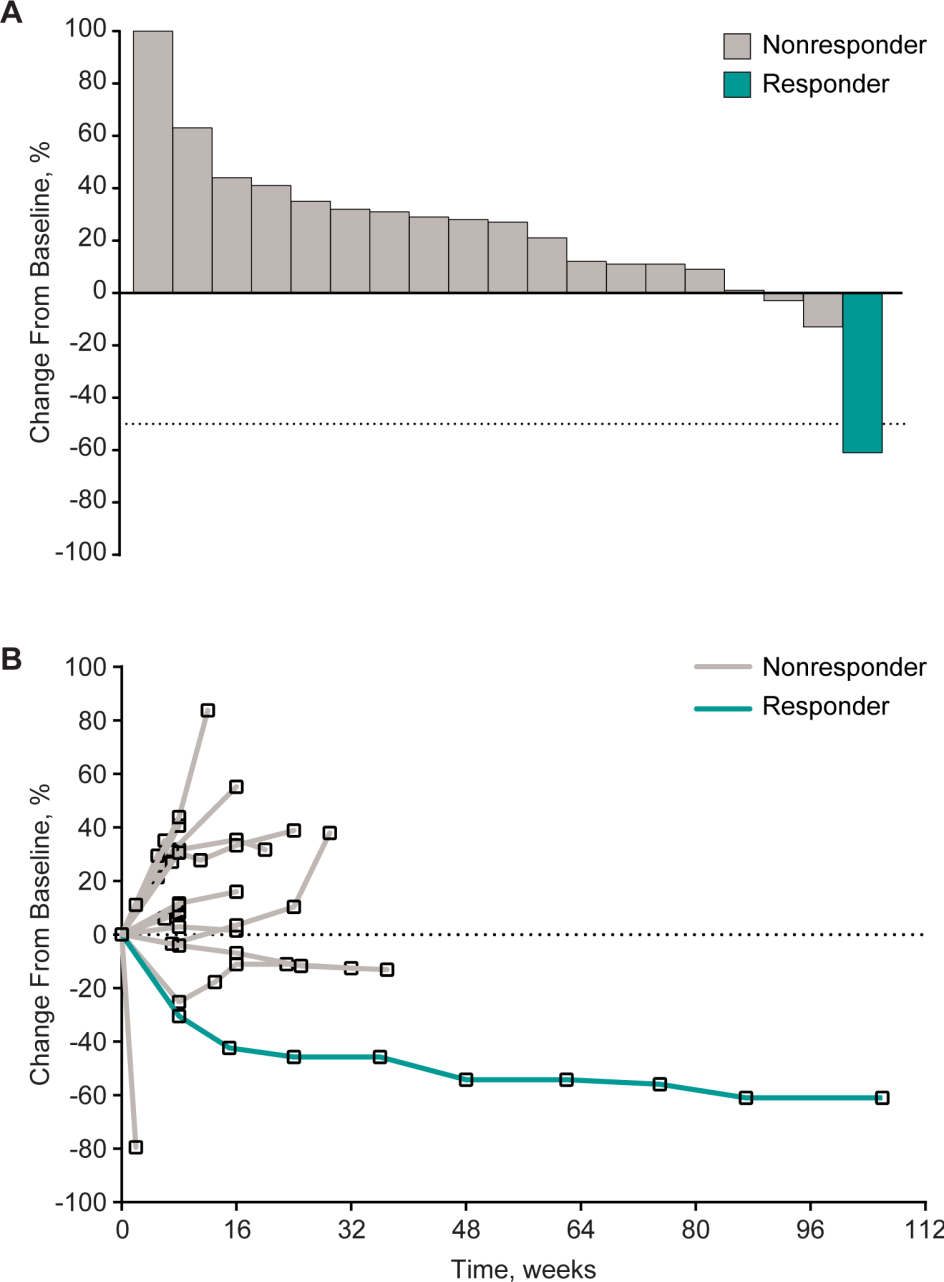
Change from baseline in tumor size. (A) Maximum change from baseline. (B) Longitudinal change from baseline.Both panels include patients with ≥1 evaluable postbaseline tumor assessment per Response Evaluation Criteria in Solid Tumors version 1.1 by investigator review (*n* = 19).

The ORR was 4% (95% confidence interval [CI], 0.1%–22%); 1 patient in this cohort experienced partial response based on RECIST v1.1 by investigator review, with a time to response of 1.7 months (Fig 2). Four patients (17%; 95% CI, 5%–39%) experienced stable disease with a median duration of 5.1 months (range, 3.6–9.3 months; Fig 2). Among the remaining patients, 15 (65%; 95% CI, 43%–84%) had progressive disease (Fig 2) and 3 were not assessed for efficacy: 2 because of discontinuation owing to AEs unrelated to treatment and 1 because of clinical progression before the first imaging assessment. There was no obvious discernible pattern with regards to number of lines of prior therapy or location of metastases between the 1 patient with partial response, the 4 patients with stable disease, and the remaining patients. The overall clinical benefit rate (proportion of patients who experienced complete response, partial response, or stable disease ≥6 months) was 13% (95% CI, 3%–34%).

There was a decrease from baseline in the size of the target lesions in 3 of 19 patients (16%) with an evaluable postbaseline tumor assessment (Fig 3). In the 1 patient who experienced partial response to treatment, the response was maintained over multiple assessments (dark blue line in Fig 3), with a response duration of 22.7 months as of the data cutoff date (blue bar with yellow triangle in Fig 2).

The 1 patient who responded to pembrolizumab treatment was a 54-year-old man who was the only patient in this cohort with MSI-H CRC. This patient’s tumor also harbored a *BRAF*^V600E^ mutation. A detailed report of this patient is described in a separate publication [14]. This patient withdrew study consent and discontinued treatment at 23.2 months, at which time there was no evidence of disease progression.

**Fig 4:**
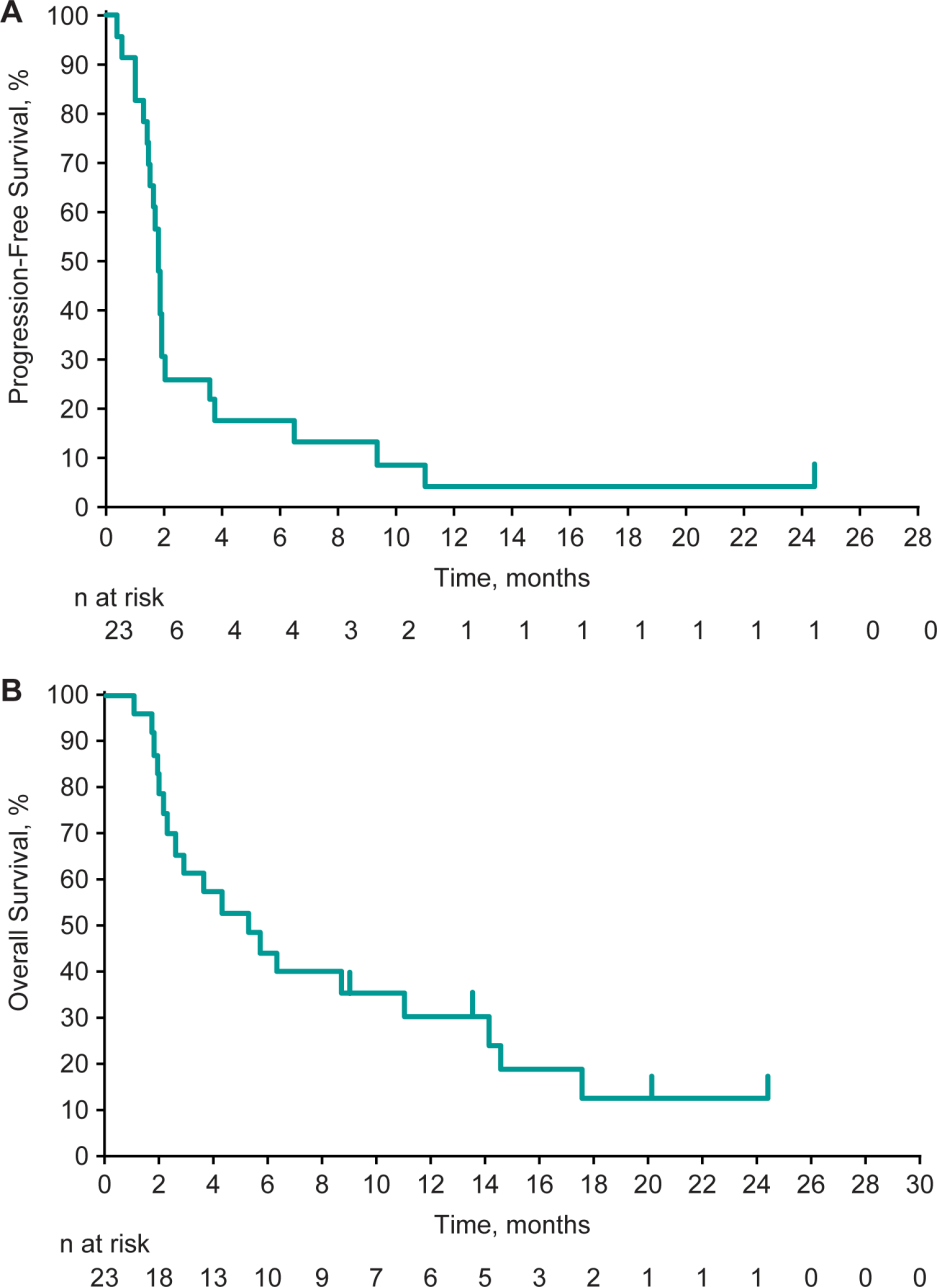
Kaplan-Meier estimates of survival. (A) Progression-free survival. (B) Overall survival.

Median PFS was 1.8 months (95% CI, 1.4–1.9 months), and the 6-month and 12-month PFS rates were 17.4% and 4.3%, respectively (Fig 4A). Median OS was 5.3 months (95% CI, 2.2–11.0 months), and the 6-month and 12-month OS rates were 43.5% and 29.8%, respectively (Fig 4B).

## Discussion

The current study indicates that pembrolizumab monotherapy was well tolerated in patients with heavily pretreated PD-L1–positive advanced CRC but that there was limited antitumor activity in this otherwise unselected patient population. The safety profile reported for this cohort was consistent with that of previous experience for pembrolizumab in advanced solid tumors [15]. Antitumor response was in line with other reported studies of anti–PD-1 therapy in advanced CRC. For example, in a phase I study of the anti–PD-L1 antibody BMS-936559 (MDX-1105) in patients with advanced cancer, no objective responses were reported in patients (*n* = 18) with advanced CRC [16]. In another phase I study, the anti–PD-1 antibody nivolumab was administered to patients who had select advanced solid tumors, including CRC (*n* = 19), again yielding no objective responses in the CRC cohort during the initial study period (treatment for up to 2 years, follow-up of patients with stable disease, or ongoing objective response for 1 year) [17]; 1 patient with MSI-H CRC ultimately experienced complete response and at last evaluation (with no antineoplastic therapy for 3 years) had no evidence of disease recurrence [18]. In line with emerging evidence for a possible correlation between TIL PD-L1 expression and response to anti–PD-1 therapy [10,11], assessment of that patient’s tumor revealed PD-L1–positive infiltrating macrophages and lymphocytes [18]. It has been suggested that because many CRC tumors do not express PD-L1, they are unlikely to respond to anti–PD-1/PD-L1 therapies [19].

In contrast with the other studies mentioned herein, all patients enrolled in the CRC cohort of KEYNOTE-028 had PD-L1–expressing tumors and response was still limited to a single patient with known MSI-H disease and a *BRAF*^V600E^ mutation. *BRAF* mutations in CRC are associated with poor prognosis [20,21], and combined MSI-H/*BRAF* mutant status has been shown to have adverse prognostic significance [22]. MSI-H CRC tumors also seem to have greater PD-L1 expression than their MSS counterparts, possibly suggesting that PD-1 checkpoint blockade may be particularly beneficial in the management of MSI-H CRC [10]. In the current study, antitumor activity was observed in MSI-H CRC (*n* = 1/1) but not in MSS CRC (*n* = 22/23), even when patients were preselected for PD-L1 expression, albeit not for equivalent levels of PD-L1 expression. These results are consistent with those of the phase II KEYNOTE-016 study of pembrolizumab in patients with MSI-H CRC and non-CRC tumors [23,24]. In KEYNOTE-016, the ORR of the CRC and non-CRC MMR-deficient (MSI-H) arms were 57% (95% CI, 39%–73%) and 53% (95% CI, 36%–70%), respectively, whereas that in the MSS CRC arm was 0% (95% CI, 0%–13%) [23,24]. Furthermore, antitumor activity was observed in only the 2 MRR-deficient arms (in which the median PFS and OS were not reached) and not in the MMR-proficient arm (median PFS of 2.3 months, median OS of 6.0 months) [23,24]. Based on these results, the US Food and Drug Administration has granted breakthrough therapy designation to pembrolizumab for the treatment of patients with MSI-H metastatic CRC.

The dose of pembrolizumab administered in the current study (10 mg/kg Q2W) is higher than that used in other studies (2 mg/kg every 3 weeks [Q3W], 10 mg/kg Q3W, or 200 mg Q3W) [15,25,26] and is higher than the currently approved doses (2 mg/kg Q3W and 200 mg Q3W) [27]. In other studies, the tolerability of pembrolizumab across all 3 of these doses was similar in cohorts of patients with various advanced solid tumors [17,28,29] The current study, albeit small, does not support any potential benefit to increasing the pembrolizumab dose as a way of improving the therapeutic response.

The following study limitations should be noted. The KETNOTE-028 study was designed as a multicohort, single-arm, non-randomized study evaluating 20 PD-L1–positive advanced solid tumor types. Because of the multicohort design, the sample size for any given tumor type, including CRC, was small (approximately 22 patients per tumor type), and there were no active comparator groups. Additionally, analyses of association between PD-L1 expression level and response to pembrolizumab were powered to be performed across the 20 advanced solid tumor types, and therefore, are not appropriate to present for any single cohort.

## Conclusion

In conclusion, the results from this small cohort of patients enrolled in the hypothesis-generating phase Ib KEYNOTE-028 trial demonstrate that pembrolizumab monotherapy has an acceptable safety profile but limited antitumor activity in patients with heavily pretreated PD-L1–positive advanced CRC. Ongoing phase II and III studies are assessing the safety and efficacy of pembrolizumab as monotherapy in previously treated advanced MSI-H or MMR-deficient CRC (KEYNOTE-164, ClinicalTrials.gov identifier: NCT02460198) and as monotherapy versus chemotherapy for advanced MSI-H CRC (KEYNOTE-177, ClinicalTrials.gov identifier: NCT02563002). These studies are also evaluating the relationship between genetic variation and response to pembrolizumab with the aim of identifying potential predictive biomarkers. Additionally, novel combinations with pembrolizumab to increase the immunogenicity of CRCs are being widely studied.

## Supporting information

S1 Protocol KEYNOTE-028 study protocol. (PDF)

S1 Checklist TREND checklist. (PDF)

## References

[1] American Cancer Society: Colorectal Cancer Facts and Figures 2014–2016. http://www.cancer.org/acs/groups/content/documents/document/acspc-042280.pdf. Accessed March 7, 2017.

[2] Siegel RL, Miller KD, Jemal A. Cancer statistics, 2016. CA Cancer J Clin 2016;66:7–30. doi: 10.3322/caac.21332 26742998 10.3322/caac.21332

[3] Torre LA, Bray F, Siegel RL, Ferlay J, Lortet-Tieulent J, Jemal A. et al Global cancer statistics, 2012. CA Cancer J Clin 2015;65:87–108. doi: 10.3322/caac.21262 25651787 10.3322/caac.21262

[4] National Cancer Institute: SEER Stat Fact Sheets: Colon and Rectum Cancer 2016. http://seer.cancer.gov/statfacts/html/colorect.html. Accessed June 13, 2016

[5] Kocarnik JM, Shiovitz S, Phipps AI. Molecular phenotypes of colorectal cancer and potential clinical applications. Gastroenterol Rep (Oxford) 2015;3:269–276. 10.1093/gastro/gov046 PMC4650976 26337942

[6] Keir ME, Butte MJ, Freeman GJ, Sharpe AH. PD-1 and its ligands in tolerance and immunity. Annu Rev Immunol 2008;26:677–704. doi: 10.1146/annurev.immunol.26.021607.090331 18173375 10.1146/annurev.immunol.26.021607.090331 PMC10637733

[7] Francisco LM, Sage PT, Sharpe AH. The PD-1 pathway in tolerance and autoimmunity. Immunol Rev 2010;236:219–242. doi: 10.1111/j.1600-065X.2010.00923.x 20636820 10.1111/j.1600-065X.2010.00923.x PMC2919275

[8] Pardoll DM. The blockade of immune checkpoints in cancer immunotherapy. Nat Rev Cancer 2012;12:252–264. doi: 10.1038/nrc3239 22437870 10.1038/nrc3239 PMC4856023

[9] Disis ML. Immune regulation of cancer. J Clin Oncol 2010;28:4531–4538. doi: 10.1200/JCO.2009.27.2146 20516428 10.1200/JCO.2009.27.2146 PMC3041789

[10] Llosa NJ, Cruise M, Tam A, Wicks EC, Hechenbleikner EM, Taube JM et al The vigorous immune microenvironment of microsatellite instable colon cancer is balanced by multiple counter-inhibitory checkpoints. Cancer Discov 2015;5:43–51. doi: 10.1158/2159-8290.CD-14-0863 25358689 10.1158/2159-8290.CD-14-0863 PMC4293246

[11] Herbst RS, Soria JC, Kowanetz M, Fine GD, Hamid O, Gordon MS et al Predictive correlates of response to the anti-PD-L1 antibody MPDL3280A in cancer patients. Nature 2014;515:563–567. doi: 10.1038/nature14011 25428504 10.1038/nature14011 PMC4836193

[12] Kerr KM, Tsao MS, Nicholson AG, Yatabe Y, Wistuba II, Hirsch FR et al Programmed death-ligand 1 immunohistochemistry in lung cancer: in what state is this art? J Thoracic Oncol 2015;10:985–989. 10.1097/JTO.0000000000000526 26134220

[13] Dolled-Filhart M, Locke D, Murphy T, Lynch F, Yearley JH, Frisman D et al Development of a prototype immunohistochemistry assay to measure programmed death ligand-1 expression in tumor tissue. Arch Pathol Lab Med 2016;140:1259–1266. doi: 10.5858/arpa.2015-0544-OA 27788043 10.5858/arpa.2015-0544-OA

[14] Sehdev A, Cramer HM, Ibrahim AA, Younger AE, O'Neil BH. Pathological complete response with anti-PD-1 therapy in a patient with microsatellite instable high, BRAF mutant metastatic colon cancer: a case report and review of literature. Discov Med 2016;21:341–347. 27355330

[15] Deeks ED. Pembrolizumab: a review in advanced melanoma. Drugs 2016;76:375–386. doi: 10.1007/s40265-016-0543-x 26846323 10.1007/s40265-016-0543-x

[16] Brahmer JR, Tykodi SS, Chow LQ, Hwu WJ, Topalian SL, Hwu P et al Safety and activity of anti-PD-L1 antibody in patients with advanced cancer. N Engl J Med 2012;366:2455–2465. doi: 10.1056/NEJMoa1200694 22658128 10.1056/NEJMoa1200694 PMC3563263

[17] Topalian SL, Hodi FS, Brahmer JR, Gettinger SN, Smith DC, McDermott DF et al Safety, activity, and immune correlates of anti-PD-1 antibody in cancer. N Engl J Med 2012;366:2443–2454. doi: 10.1056/NEJMoa1200690 22658127 10.1056/NEJMoa1200690 PMC3544539

[18] Lipson EJ, Sharfman WH, Drake CG, Wollner I, Taube JM, Anders RA et al Durable cancer regression off-treatment and effective reinduction therapy with an anti-PD-1 antibody. Clin Cancer Res 2013;19:462–468. doi: 10.1158/1078-0432.CCR-12-2625 23169436 10.1158/1078-0432.CCR-12-2625 PMC3548952

[19] Norton SE, Ward-Hartstonge KA, Taylor ES, Kemp RA. Immune cell interplay in colorectal cancer prognosis. World J Gastrointestinal Oncol 2015;7:221–232. 10.4251/wjgo.v7.i10.221 PMC4606176 26483876

[20] Roth AD, Tejpar S, Delorenzi M, Yan P, Fiocca R, Klingbiel D et al Prognostic role of KRAS and BRAF in stage II and III resected colon cancer: results of the translational study on the PETACC-3, EORTC 40993, SAKK 60–00 trial. J Clin Oncol 2010;28:466–474. doi: 10.1200/JCO.2009.23.3452 20008640 10.1200/JCO.2009.23.3452

[21] Therkildsen C, Bergmann TK, Henrichsen-Schnack T, Ladelund S, Nilbert M. The predictive value of KRAS, NRAS, BRAF, PIK3CA and PTEN for anti-EGFR treatment in metastatic colorectal cancer: a systematic review and meta-analysis. Acta Oncol 2014;53:852–864. doi: 10.3109/0284186X.2014.895036 24666267 10.3109/0284186X.2014.895036

[22] Lochhead P, Kuchiba A, Imamura Y, Liao X, Yamauchi M, Nishihara R et al Microsatellite instability and BRAF mutation testing in colorectal cancer prognostication. J National Cancer Inst 2013;105:1151–1156. 10.1093/jnci/djt173 PMC3735463 23878352

[23] Le DT, Uram JN, Wang H, Bartlett B, Kemberling H, Eyring A et al Programmed death-1 blockade in mismatch repair deficient colorectal cancer. J Clin Oncol 2016;34(suppl):abstr 103.

[24] Diaz LA, Uram JN, Wang H, Bartlett B, Kemberling H, Eyring A et al Programmed death-1 blockade in mismatch repair deficient cancer independent of tumor histology. J Clin Oncol 2016;34(suppl): abstr 3003.

[25] Langer CJ, Gadgeel SM, Borghaei H, Papadimitrakopoulou VA, Patnaik A, Powell SF et al Carboplatin and pemetrexed with or without pembrolizumab for advanced, non-squamous non-small-cell lung cancer: a randomised, phase 2 cohort of the open-label KEYNOTE-021 study. Lancet Oncol 2016;17:1497–1508. doi: 10.1016/S1470-2045(16)30498-3 27745820 10.1016/S1470-2045(16)30498-3 PMC6886237

[26] Chow LQ, Haddad R, Gupta S, Mahipal A, Mehra R, Tahara M et al Antitumor activity of pembrolizumab in biomarker-unselected patients with recurrent and/or metastatic head and neck squamous cell carcinoma: results from the phase Ib KEYNOTE-012 expansion cohort. J Clin Oncol. 2016; 34: 3838–3845. doi: 10.1200/JCO.2016.68.1478 27646946 10.1200/JCO.2016.68.1478 PMC6804896

[27] Keytruda [package insert]. Whitehouse Station, NJ: Merck Sharp & Dohme Corp.; 2017

[28] Patnaik A, Kang SP, Rasco D, Papadopoulos KP, Elassaiss-Schaap J, Beeram M et al Phase I study of pembrolizumab (MK-3475; anti-PD-1 monoclonal antibody) in patients with advanced solid tumors. Clin Cancer Res 2015;21:4286–4293. doi: 10.1158/1078-0432.CCR-14-2607 25977344 10.1158/1078-0432.CCR-14-2607

[29] Garon EB, Rizvi NA, Hui R, Leighl N, Balmanoukian AS, Eder JP et al Pembrolizumab for the treatment of non-small-cell lung cancer. N Engl J Med 2015;372:2018–2028. doi: 10.1056/NEJMoa1501824 25891174 10.1056/NEJMoa1501824

