# Supplementary material for: Safety and antitumor activity of the anti–PD-1 antibody pembrolizumab in patients with advanced colorectal carcinoma
Source: PLoS One. 2017 Dec 28;12(12):e0189848. doi: 10.1371/journal.pone.0189848 (PMC5746232; doi:10.1371/journal.pone.0189848)
Supplement: S1 Protocol — (PDF) [file pone.0189848.s001.pdf]

Merck & Co., Inc. policy on posting of redacted study protocols on journal websites is described in Guidelines for Publication of Clinical Trials in the Scientific Literature on the www.merck.com website.

For publicly posted protocols, the Company redacts the background and rationale sections because these sections may contain proprietary information. The Company also redacts the names of any individuals due to privacy issues. The appendices, generally, are not provided because they may be lengthy and contain non-essential information. The publicly posted protocol includes all the key sections that are relevant to evaluating the study, specifically those sections describing the study objectives and hypotheses, the patient inclusion and exclusion criteria, the study design and procedures, the efficacy and safety measures, the statistical analysis plan, and amendments relating to those sections.

This report may include approved and non-approved uses, formulations, or treatment regimens. The results reported may not reflect the overall profile of a product. Before prescribing any product mentioned in this report, healthcare professionals should consult local prescribing information for the product approved in their country.

Copyright © 2016 Merck Sharp & Dohme Corp., a subsidiary of Merck & Co., Inc.

All Rights Reserved. Not for regulatory or commercial use.

THIS PROTOCOL AMENDMENT AND ALL OF THE INFORMATION RELATING TO IT ARE CONFIDENTIAL AND PROPRIETARY PROPERTY OF MERCK SHARP & DOHME CORP., A SUBSIDIARY OF MERCK & CO., INC., WHITEHOUSE STATION, NJ, U.S.A.

SPONSOR:

Merck Sharp & Dohme Corp., a subsidiary of Merck & Co., Inc.
(hereafter referred to as the Sponsor or Merck)
One Merck Drive
P.O. Box 100
Whitehouse Station, NJ 08889-0100, U.S.A.

Protocol-specific Sponsor Contact information can be found in the Investigator Trial File Binder (or equivalent).

TITLE:

Phase IB Study of Pembrolizumab (MK-3475) in Subjects with Select Advanced Solid Tumors

IND NUMBER: 110,080
EudraCT NUMBER: 2013-004507-39

TABLE OF CONTENTS

SUMMARY OF CHANGES 10
1.0 TRIAL SUMMARY 16
2.0 TRIAL DESIGN 17
2.1 Trial Design 17
2.2 Trial Diagram 19
3.0 OBJECTIVE(S) & HYPOTHESIS(ES) 20
3.1 Primary Objective(s) & Hypothesis(es) 20
3.2 Secondary Objective(s) & Hypothesis(es) 21
3.3 Exploratory Objective 21
4.0 BACKGROUND & RATIONALE 22
4.1 Background 22
4.1.1 Pharmaceutical and Therapeutic Background 22
4.1.2 Pre-clinical and Clinical Trials 23
4.1.3 Ongoing Clinical Trials 23
4.2 Rationale 23
4.2.1 Rationale for the Trial and Selected Subject Population 23
4.2.2 Rationale for Dose Selection/Regimen/Modification 24
4.2.3 Rationale for Endpoints 24
4.2.3.1 Efficacy Endpoints 24
4.2.3.2 Safety Endpoints 26
4.2.3.3 Biomarker Research 26
4.2.3.4 Future Biomedical Research 27
5.0 METHODOLOGY 28
5.1 Entry Criteria 28
5.1.1 Diagnosis/Condition for Entry into the Trial 28
5.1.2 Subject Inclusion Criteria 28
5.1.3 Subject Exclusion Criteria 30
5.2 Trial Treatment(s) 32
5.2.1 Dose Selection/Modification 33
5.2.1.1 Dose Selection (Preparation) 33

5.2.1.2 Dose Modification 33
5.2.2 Timing of Dose Administration 35
5.2.3 Trial Blinding/Masking 36
5.3 Randomization or Treatment Allocation 36
5.4 Stratification 36
5.5 Concomitant Medications/Vaccinations (Allowed & Prohibited) 37
5.5.1 Acceptable Concomitant Medications 37
5.5.2 Prohibited Concomitant Medications 37
5.6 Rescue Medications & Supportive Care 38
5.6.1 Supportive Care Guidelines 38
5.6.1.1 Supportive Care Guidelines for Events of Clinical Interest and Immune-related Adverse Events (irAEs) 41
5.6.1.2 Supportive Care Guidelines for Pneumonitis 41
5.7 Diet/Activity/Other Considerations 41
5.7.1 Diet 41
5.7.2 Contraception 41
5.7.3 Use in Pregnancy 42
5.7.4 Use in Nursing Women 42
5.8 Subject Withdrawal/Discontinuation Criteria 42
5.8.1 Discontinuation of Study Therapy after CR 44
5.9 Subject Replacement Strategy 44
5.10 Beginning and End of the Trial 44
5.11 Clinical Criteria for Early Trial Termination 44
6.0 TRIAL FLOW CHART 46
6.1 Study Flow Chart 46
6.2 Second Course Phase (Retreatment ONLY) 50
7.0 TRIAL PROCEDURES 52
7.1 Trial Procedures 52
7.1.1 Administrative Procedures 52
7.1.1.1 Informed Consent 52
7.1.1.1.1 General Informed Consent 52

7.1.1.1.2 Consent and Collection of Specimens for Future Biomedical Research 53
7.1.1.2 Inclusion/Exclusion Criteria 53
7.1.1.3 Subject Identification Card 53
7.1.1.4 Medical History 53
7.1.1.5 Prior and Concomitant Medications Review 53
7.1.1.5.1 Prior Medications 53
7.1.1.5.2 Concomitant Medications 53
7.1.1.6 Disease Details and Treatments 54
7.1.1.6.1 Disease Details 54
7.1.1.6.2 Prior Treatment Details 54
7.1.1.6.3 Subsequent Anti-Cancer Therapy Status 54
7.1.1.7 Assignment of Screening Number 54
7.1.1.8 Assignment of Randomization Number 54
7.1.1.9 Trial Compliance (Medication/Diet/Activity/Other) 54
7.1.2 Clinical Procedures/Assessments 55
7.1.2.1 Adverse Event (AE) Monitoring 55
7.1.2.2 Physical Exam 55
7.1.2.2.1 Full Physical Exam 55
7.1.2.2.2 Directed Physical Exam 55
7.1.2.3 Vital Signs 56
7.1.2.4 Eastern Cooperative Oncology Group (ECOG) Performance Scale 56
7.1.2.5 Tumor Imaging and Assessment of Disease 56
7.1.2.5.1 Assessment of Disease 56
7.1.2.5.2 Initial Tumor Imaging 58
7.1.2.5.3 Tumor Imaging During Trial 58
7.1.2.6 Tumor Tissue Collection and Correlative Studies Blood Sampling 58
7.1.2.7 Tumor Marker Assessment (Optional) 59
7.1.3 Laboratory Procedures/Assessments 59
7.1.3.1 Laboratory Safety Evaluations (Hematology, Chemistry and Urinalysis) .. 60
7.1.3.2 Future Biomedical Research 62
7.1.4 Other Procedures 62

7.1.4.1 Withdrawal/Discontinuation 62
7.1.4.1.1 Withdrawal From Future Biomedical Research 62
7.1.4.2 Blinding/Unblinding 63
7.1.4.3 Calibration of Critical Equipment 63
7.1.5 Visit Requirements 63
7.1.5.1 Screening Period 63
7.1.5.2 Treatment Period 64
7.1.5.2.1 Second Course Phase (Retreatment Period) 64
7.1.5.3 Post-Treatment Visits 65
7.1.5.3.1 Safety Follow-Up Visit 65
7.1.5.4 Follow-up Visits 66
7.1.5.4.1 Survival Follow-up 66
7.2 Assessing and Recording Adverse Events 66
7.2.1 Definition of an Overdose for This Protocol and Reporting of Overdose to the Sponsor 67
7.2.2 Reporting of Pregnancy and Lactation to the Sponsor 68
7.2.3 Immediate Reporting of Adverse Events to the Sponsor 68
7.2.3.1 Serious Adverse Events 68
7.2.3.2 Events of Clinical Interest 69
7.2.3.3 Protocol-Specific Exceptions to Serious Adverse Event Reporting 70
7.2.4 Evaluating Adverse Events 70
7.2.5 Sponsor Responsibility for Reporting Adverse Events 73
8.0 STATISTICAL ANALYSIS PLAN 73
8.1 Statistical Analysis Plan Summary 73
8.1.1 Efficacy Analyses 73
8.1.2 Safety Analyses 74
8.1.3 Power and Sample Size 74
8.1.4 Interim Analysis 76
8.2 Statistical Analysis Plan 77
8.2.1 Responsibility for Analyses/In-House Blinding 77
8.2.2 Hypotheses/Estimation 77
8.2.3 Analysis Endpoints 77

8.2.3.1 Efficacy Endpoints 77
8.2.3.2 Safety Endpoints 78
8.2.4 Analysis Populations 78
8.2.4.1 Efficacy Analysis Populations 78
8.2.4.2 Safety Analysis Populations 78
8.2.5 Statistical Methods 79
8.2.5.1 Statistical Methods for Efficacy Analyses 79
8.2.5.2 Statistical Methods for Safety Analyses 80
8.2.5.3 Summaries of Baseline Characteristics, Demographics, and Other Analyses 81
8.2.5.3.1 Demographic and Baseline Characteristics 81
8.2.6 Multiplicity 81
8.2.7 Sample Size and Power Calculations 81
8.2.8 Subgroup Analyses and Effect of Baseline Factors 84
8.2.9 Interim Analyses 84
8.2.10 Compliance (Medication Adherence) 84
8.2.11 Extent of Exposure 85
9.0 LABELING, PACKAGING, STORAGE AND RETURN OF CLINICAL SUPPLIES 85
9.1 Investigational Product 85
9.2 Packaging and Labeling Information 85
9.3 Clinical Supplies Disclosure 85
9.4 Storage and Handling Requirements 85
9.5 Returns and Reconciliation 86
9.6 Standard Policies 86
10.0 ADMINISTRATIVE AND REGULATORY DETAILS 86
10.1 Confidentiality 86
10.1.1 Confidentiality of Data 86
10.1.2 Confidentiality of Subject Records 86
10.1.3 Confidentiality of Investigator Information 87
10.1.4 Confidentiality of IRB/IEC Information 87
10.2 Compliance with Financial Disclosure Requirements 87

10.3 Compliance with Law, Audit and Debarment 88
10.4 Compliance with Trial Registration and Results Posting Requirements 90
10.5 Quality Management System 90
10.6 Data Management 90
10.7 Publications 90
11.0 LIST OF REFERENCES 92
12.0 APPENDICES 95
12.1 Merck Code of Conduct for Clinical Trials 95
12.2 Collection and Management of Specimens for Future Biomedical Research 97
12.3 Understanding the Intent, Scope and Public Health Benefits of Exploratory Biomarker Research: A Guide for IRBs/IECs and Investigational Site Staff 103
12.4 ECOG Performance Status 114
12.5 Common Terminology Criteria for Adverse Events V4.0 (CTCAE) 115
12.6 Response Evaluation Criteria in Solid Tumors (RECIST) 1.1 Criteria For Evaluating Response in Solid Tumors 116
12.7 Updated Response Assessment Criteria for High-Grade Gliomas: Response Assessment in Neuro-Oncology Working Group 117
12.8 Design and End Points of Clinical Trials for Patients With Progressive Prostate Cancer and Castrate Levels of Testosterone: Recommendations of the Prostate Cancer Clinical Trials Working Group 118
12.9 List of Abbreviations 119
13.0 SIGNATURES 122
13.1 Sponsor's Representative 122
13.2 Investigator 122

LIST OF TABLES

Table 1 Adequate Organ Function Laboratory Values 30
Table 2 Trial Treatment 32
Table 3 Dose Modification Guidelines for Drug-Related Adverse Events 34
Table 4 Imaging and Treatment After 1st Radiologic Evidence of PD 36
Table 5 Infusion Reaction Treatment Guidelines 40
Table 6 Imaging and Treatment after 1st Radiologic Evidence of PD 57
Table 7 Tumor Markers and Associated Tumor Type 59
Table 8 Laboratory Tests 61
Table 9 Evaluating Adverse Events 71
Table 10 Summary of Analysis Strategy for Key Efficacy Endpoints 74
Table 11 Decision Rules Based on Efficacy Bounds 76
Table 12 Decision Rules Based on Futility Bounds 76
Table 13 Summary of Interim Analysis Strategy 77
Table 14 Analysis Strategy for Key Efficacy Variables 80
Table 15 Operating Characteristics of the Sequential Monitoring Approach 83
Table 16 Point Estimate and 95% CI for Hypothetical Number of Subjects with a Grade 3-5 irAE 84
Table 17 Product Descriptions 85

LIST OF FIGURES

Figure 1 Trial Design 19
Figure 2 Sequential Monitoring Rules for Efficacy and Futility 75

SUMMARY OF CHANGES

PRIMARY REASON(S) FOR THIS AMENDMENT:

Section Number (s)	Section Title(s)	Description of Change (s)
5.2;<br>5.2.1.2;<br>5.6.1;<br>5.6.1.1;<br><br><br><br>5.6.1.2;<br><br>7.2.3.2	Trial Treatment;<br>Dose Modification;<br>Supportive Care Guidelines;<br>Supportive Care Guidelines for Events of Clinical Interest and Immune-related Adverse Events (irAEs);<br>Supportive Care Guidelines for Pneumonitis<br>Events of Clinical Interest	Dose modification language, supportive care guidelines, and Events of Clinical Interest (ECI) guidance revised with updated ECI guidance document. Detailed description of changes can be found in the "Additional Change(s) for this Amendment" sections.<br><br>RATIONALE: Clarification and consistency in alignment with program level pembrolizumab ECI and dose modification guidance.

ADDITIONAL CHANGE(S) FOR THIS AMENDMENT:

Section Number (s)	Section Title (s)	Description of Change (s)
All sections	Global	Replaced "MK-3475" with "pembrolizumab (MK-3475)"<br><br>RATIONALE: Implemented utilization of generic term 'pembrolizumab' for MK-3475.
All sections	Global	Replaced "Event of Clinical Interest and Immune-Related Adverse Event Guidance Document" with "Event of Clinical Interest Guidance Document"<br><br>RATIONALE: Alignment with title of separate guidance document recently updated
All sections	Global	Replaced "Procedures Manual" with "Pharmacy Manual" in references to drug preparation and administration<br><br>RATIONALE: Dose calculation, preparation of the infusion solution, and administration details removed from Procedures Manual into a separate Pharmacy Manual

Section Number (s)	Section Title (s)	Description of Change (s)
3.1	Primary Objective(s) & Hypothesis(es)	• Add "greater than 10%" to reflect the null hypothesis<br>• Add 'best' before 'overall response rate' to be consistent with the whole protocol<br><br>RATIONALE: To be consistent with the pembrolizumab (MK-3475) program wide objectives for best ORR to have null hypothesis rates included. In KEYNOTE-028 actual analysis, 10% was used as a null hypothesized rate.
4.2.2	Rationale for Dose Selection/ Regimen/Modification	Replaced "protocol" with "KEYNOTE" for certain Merck clinical trials<br><br>RATIONALE: Implemented utilization of "KEYNOTE" designation for MK-3475 trials.
4.2.3.1; 7.1.2.5.1; 12.6	Efficacy Endpoints; Assessment of Disease; RECIST Appendix	Removed references to volumetric analyses.<br><br>RATIONALE: May not provide additional information beyond RECIST, and adds complexity to the study
4.2.3.1	Efficacy Endpoints	Clarified subjects in the GBM and prostate cohorts will undergo RECIST assessments.<br><br>RATIONALE: GBM subjects were always intended to have RECIST (as well as RANO assessments. PCWG2 similarly were always intended to have both PCWG2 and RECIST assessments.
4.2.3.1; 6.1; 6.2; 7.1.2.5	Efficacy Endpoints; Study Flow Chart 'Footnote p'; Study Flow Chart 'Footnote k'; Tumor Imaging and Assessment of Disease	For imaging guidance, references to the procedure manual removed and replaced by Site Imaging Manual (SIM).<br><br>RATIONALE: To clarify which document contains specific details on image acquisition rather than cross referencing the Procedures Manual.
5.1.2; 5.5.2	Subject Inclusion Criteria; Prohibited Concomitant Medications	Exclusion criterion #4 foonote 'c' revised:<br>• Glioblastoma multiforme subjects with any prior bevacizumab treatment are NOT eligible (exceptions may be considered after consultation with the sponsor).<br><br>RATIONALE: External scientific recommendation to allow greater flexibility in enrolling patients in the GBM cohort

Section Number (s)	Section Title (s)	Description of Change (s)
5.1.3	Subject Exclusion Criteria	Exclusion criteria #1, #7 and #13 revised:<br>• Is currently participating and receiving study therapy or has participated in a study of an investigational agent and received study therapy or used an investigation device within 4 weeks of the first dose of treatment.<br>• Has an active autoimmune disease that has required systemic treatment in past 2 years (i.e. with use of disease modifying agents, corticosteroids or immunosuppressive drugs). Replacement therapy (e.g., thyroxine, insulin, or physiologic corticosteroid replacement therapy for adrenal or pituitary insufficiency, etc.) is not considered a form of systemic treatment.<br>• Has previously participated in any other pembrolizumab (MK-3475) trial, or received prior therapy with an anti-PD-1, anti-PD-L1, and anti-PD-L2 (including ipilimumab or any other antibody or drug specifically targeting T-cell co-stimulation or checkpoint pathways).<br><br>RATIONALE: Clarification and consistency in alignment with program level exclusion criteria.
5.2;<br><br>5.2.1.2	Trial Treatment;<br><br>Dose Modification	The following table updated:<br>• Table 2 Trial Treatment footnote updated (replaced "increased" with "withheld")<br>• Table 3 Dose Modification Guidelines for Drug-Related Adverse Events – new table inserted<br><br>The following text removed:<br>• ~~MK-3475 will be withheld for drug-related Grade ≥3 hematologic toxicities (excluding Grade 3 neutropenia, anemia, and thrombocytopenia), non-hematological toxicity ≥ Grade 3 including laboratory abnormalities, and severe or life-threatening AEs as per Table 3 below.~~<br><br>The following modifications made:<br>• In case toxicity **related to pembrolizumab (MK-3475)** does not resolve to Grade 0-1 within 12 weeks after last infusion, trial treatment should be discontinued ~~after consultation with the Sponsor. With investigator and Sponsor agreement,~~ Subjects with a laboratory adverse event still at Grade 2 after 12 weeks may continue treatment in the trial only if asymptomatic and controlled. For information on the management of adverse events, see Section 5.6.1.<br><br>RATIONALE: Clarification and consistency in alignment with program level pembrolizumab ECI and dose modification guidance.

Section Number (s)	Section Title (s)	Description of Change (s)
5.5.2	Prohibited Concomitant Medications	Sponsor consultation required for the use of glucocorticoids for any other purpose than to modulate symptoms of an AE.<br><br>RATIONALE: Allow glucocorticoid use for treatment of AEs beyond just immune related AEs, while still having steroid use monitored by the Sponsor
5.6.1;<br>5.6.1.1;<br><br><br><br>5.6.1.2;<br><br>7.2.3.2	Supportive Care Guidelines;<br>Supportive Care Guidelines for Events of Clinical Interest and Immune-related Adverse Events (irAEs);<br>Supportive Care Guidelines for Pneumonitis;<br>Events of Clinical Interest	• Removed supportive care guidelines for moderate and severe enterocolitis<br>• Updated language in entire section entitled "Supportive Care Guidelines for Events of Clinical Interest and Immune-related Adverse Events (irAEs)" and removed Table 6 "General Approach to Handling irAEs"<br>• Removed dose modification for pneumonitis which includes removal of Table 7 "Recommended Approach to Handling Pneumonitis"<br>• Updated language in section entitled "Events of Clinical Interest" item 3 "Additional Adverse Events"<br><br>RATIONALE: Clarification and consistency in alignment with program level pembrolizumab ECI guidance
6.1; 6.2;<br>7.1.5.3	Study Flow Chart;<br>Second Course Phase;<br>Post-Treatment Visits	Updates and clarifications made:<br>• Visit schedule for Safety Follow-up visit changed to 30 days +/- 3 days **after last dose**<br>• Visit window for Imaging Follow-up visits updated to +/- 7 days<br>• Visit window for Survival Follow-up visits updated to +/- 4 weeks<br><br>RATIONALE: To provide clarification on post treatment visit windows and clarify the 30 day Safety Follow up should be after last dose (not discontinuation date).
6.1; 6.2;<br>7.1.5.3	Study Flow Chart 'Footnote f & g'; Second Course Phase 'Footnote d & f'; Post-Treatment Visits	Clarified post-treatment AE and concomitant medications reporting timeframes<br><br>RATIONALE: To provide clarification.

Section Number (s)	Section Title (s)	Description of Change (s)
7.1.3.1	Laboratory Safety Evaluations	Table 10 Laboratory Tests updated to include the following:<br>• Free T3 (FT3) may be performed instead of Total T3 per local standards<br>• GFR (measured or calculated) or CrCl can be used in place of creatinine<br><br>RATIONALE: Additional labs added to accommodate local standards.
7.1.4.3	Calibration of Critical Equipment	Replaced "Site Operations Manual" with "Site Imaging Manual"<br><br>RATIONALE: Site Operations Manual and Site Imaging Manual are interchangeable.
8.1.1	Efficacy Analyses	Change 'A sequential monitoring approach will be used following the time that a minimum of 6 subjects are enrolled in each indication' to 'A sequential monitoring approach will be used following the time that a minimum of 6 subjects have had at least one post-baseline scan in each indication.'<br><br>RATIONALE: To be clear about the practice for interim monitoring and be consistent with the descriptions of the sequential monitoring approach in other sections.
8.1.3	Power and Sample Size	• Figure 2 Sequential Monitoring Rules for Efficacy and Futility: updated to incorporate scenarios with more than 22 subjects<br>• Table 11 Decision Rules Based on Efficacy Bounds: computation errors corrected<br>• Table 12 Decision Rules Based on Futility Bounds: updated to meet the needs of a potential increase of sample size in some indications<br><br>RATIONALE: Provide efficacy bounds and futility bounds for possible scenarios where more than 22 subjects are enrolled in a given indication
8.2.4.1	Efficacy Analysis Populations	FAS-2 and ITT populations are removed to be consistent with the whole program<br><br>RATIONALE: FAS analysis will be sole primary analysis. FAS-2 and ITT population will not be used in the final analyses, they are removed to be consistent with other protocols.

Section Number (s)	Section Title (s)	Description of Change (s)
8.2.5.1	Statistical Methods for Efficacy Analyses	• FAS-2 and ITT analyses are removed<br>• Table 16, change the title 'Secondary Endpoints/Hypotheses – Within Indication' to 'Secondary Endpoints/Objectives – Within Indication'<br><br>RATIONALE: Please refer to 8.2.4.1
8.2.7	Sample Size and Power Calculations	• Table 16 Point Estimate and 95% CI for Hypothetical Number of Subjects with a Grade 3-5 irAE: updated to incorporate cases with possible 440 subjects<br>• Add 'best' before 'ORR' in order to be clear and consistent with the whole protocol<br><br>RATIONALE: To provide results of point estimate and CI for scenarios with 440 subjects for completeness
8.2.9	Interim Analyses	Clarify that for purpose of interim monitoring, subjects who are still on study but without a post baseline scan will be excluded from the analyses specified in Table 14<br><br>RATIONALE: For interim analyses, the FAS population is not appropriate for denominator as subjects may not have the first valid tumor assessment post baseline while on study. The best overall response rate will much lower than what it should be. This change is to clarify what denominator should be used for computing best ORR in interim analyses.

1.0 TRIAL SUMMARY

Abbreviated Title	Pembrolizumab (MK-3475) Solid All Comers
Trial Phase	1B
Clinical Indication	The treatment of subjects with any of the following advanced (unresectable and/or metastatic) solid tumor indications:<br><br>A1 Colon or Rectal Adenocarcinoma<br>A2 Anal Canal Squamous Cell Carcinoma<br>A3 Pancreas Adenocarcinoma<br>A4 Esophageal Squamous Cell Carcinoma or Adenocarcinoma (Including GE Junction)<br>A5 Biliary Tract Adenocarcinoma (Gallbladder and Biliary Tree but excluding Ampulla of Vater Cancers)<br>A6 Carcinoid Tumors<br>A7 Neuroendocrine Carcinomas (Well or moderately differentiated Pancreatic Neuroendocrine Tumor)<br><br>B1 ER Positive HER2 Negative Breast Cancer<br>B2 Ovarian Epithelial, Fallopian Tube or Primary Peritoneal Carcinoma<br>B3 Endometrial Carcinoma<br>B4 Cervical Squamous Cell Cancer<br>B5 Vulvar Squamous Cell Carcinoma<br><br>C1 Small Cell Lung Cancer<br>C2 Mesothelioma (Malignant Pleural Mesothelioma)<br><br>D1 Thyroid Cancer (Papillary or Follicular Subtype)<br>D2 Salivary Gland Carcinoma<br>D3 Nasopharyngeal Carcinoma<br><br>E1 Glioblastoma Multiforme<br>E2 Leiomyosarcoma<br>E3 Prostate Adenocarcinoma
Trial Type	Interventional
Type of control	No treatment control
Route of administration	Intravenous
Trial Blinding	Unblinded Open-label
Treatment Groups	MK-3475 10mg/kg every 2 weeks
Number of trial subjects	Approximately 320 subjects will be enrolled.
Estimated duration of trial	The sponsor estimates that the trial will require approximately 24 months from the time the first subject signs the informed consent until the last subject's last visit.
Duration of Participation	Each subject will participate in the trial from the time the subject signs the Informed Consent Form (ICF) through the final protocol-specified contact. After a screening phase of 28 days, eligible subjects will receive treatment on Day 1 of each 2-week dosing cycle. Treatment with MK-3475 will continue until documented confirmed disease progression, unacceptable adverse event(s), intercurrent illness that prevents further administration of treatment, investigator's decision to withdraw the subject, subject withdraws consent, pregnancy of the subject,

	noncompliance with trial treatment or procedure requirements; subject receives 24 months of study medication, or administrative reasons. Subjects who attain a complete response may consider stopping trial treatment if they meet criteria for holding therapy. Subjects who stop trial treatment after receiving 24 months of study medication for reasons other than disease progression or intolerability or who attain a complete response and stop trial treatment may be eligible for up to one year of retreatment after experiencing disease progression. The decision to retreat will be at the discretion of the investigator only if they meet the criteria for retreatment and the trial is ongoing. After the end of treatment, each subject will be followed for 30 days for adverse event monitoring (serious adverse events will be collected for 90 days after the end of treatment). Subjects who discontinue for reasons other than disease progression will have post-treatment follow-up for disease status until disease progression, initiating a non-study cancer treatment, withdrawing consent, or becoming lost to follow-up. All subjects will be followed by telephone for overall survival until death, withdrawal of consent, or the end of the study.

A list of abbreviations used in this document can be found in Section 12.9.

2.0 TRIAL DESIGN

2.1 Trial Design

This is a multicenter, nonrandomized, multi-cohort trial of pembrolizumab (MK-3475) in subjects with PD-L1 positive advanced solid tumors. Subjects will be enrolled into one of the following 20 solid tumor cohorts:

A1 Colon or Rectal Adenocarcinoma
A2 Anal Canal Squamous Cell Carcinoma
A3 Pancreas Adenocarcinoma
A4 Esophageal Squamous Cell Carcinoma or Adenocarcinoma (Including GE Junction)
A5 Biliary Tract Adenocarcinoma (Gallbladder and Biliary Tree but excluding Ampulla of Vater Cancers)
A6 Carcinoid Tumors
A7 Neuroendocrine Carcinomas (Well or moderately differentiated Pancreatic Neuroendocrine Tumor)

B1 ER Positive HER2 Negative Breast Cancer
B2 Ovarian Epithelial, Fallopian Tube or Primary Peritoneal Carcinoma
B3 Endometrial Carcinoma
B4 Cervical Squamous Cell Cancer
B5 Vulvar Squamous Cell Carcinoma

C1 Small Cell Lung Cancer
C2 Mesothelioma (Malignant Pleural Mesothelioma)

D1 Thyroid Cancer (Papillary or Follicular Subtype)
D2 Salivary Gland Carcinoma
D3 Nasopharyngeal Carcinoma

E1 Glioblastoma Multiforme
E2 Leiomyosarcoma
E3 Prostate Adenocarcinoma

Approximately 320 subjects will be enrolled in this trial to examine the safety and efficacy in these cohorts to the 10mg/kg dose of pembrolizumab (MK-3475) administered every 2 weeks. Subjects will be evaluated every 8 weeks (56 days ±7 days) with radiographic imaging to assess response to treatment. After 6 months, radiography imaging will be evaluated every 12 weeks (84 days ±7 days). RECIST 1.1 will be used as the primary efficacy endpoint of response rate.

RECIST 1.1 will be adapted as described in Section 4.2.3.1 due to the tumor response patterns seen with pembrolizumab (MK-3475) treatment (e.g., tumor flare), and this adapted RECIST will be used by the sites for treatment decisions. Adverse events will be monitored throughout the trial and graded in severity according to the guidelines outlined in the NCI Common Terminology Criteria for Adverse Events (CTCAE) version 4.0. Treatment with pembrolizumab (MK-3475) will continue until documented disease progression, unacceptable adverse event(s), intercurrent illness that prevents further administration of treatment, investigator's decision to withdraw the subject, subject withdraws consent, pregnancy of the subject, noncompliance with trial treatment or procedure requirements, completion of 24 months of treatment with pembrolizumab (MK-3475), or administrative reasons. Subjects who attain an investigator-determined confirmed complete response (CR) may consider stopping trial treatment after receiving at least 24 weeks of treatment. In addition, subjects who discontinue after at least 24 months of therapy for reasons other than disease progression or intolerability, or who discontinue after attaining a CR and had at least 2 treatments beyond initial CR, may be eligible for up to one year of retreatment if they subsequently experience radiographic disease progression. The decision to retreat will be at the discretion of the investigator only if no cancer treatment was administered since the last dose of pembrolizumab (MK-3475), the subject still meets the safety parameters listed in the Inclusion/Exclusion criteria and the trial remains open (refer to Section 7.1.5.2.1 for further details). After the end of treatment, each subject will be followed for 30 days for adverse event monitoring (serious adverse events will be collected for 90 days after the end of treatment). Subjects who discontinue treatment for reasons other than disease progression will have post-treatment follow-up of disease status until disease progression, initiating a non-study cancer treatment, withdrawing consent, or becoming lost to follow-up. All subjects will be followed by telephone contact for overall survival until death, withdrawal of consent or the end of the study, whichever comes first.

The primary objective of the trial is to evaluate a preliminary signal of potential anti-tumor activity of pembrolizumab (MK-3475) in subjects with PD-L1 positive advanced solid tumors. Secondary objectives include safety and tolerability, progression-free survival (PFS), overall survival (OS) and response duration.

Participation in this trial will be dependent upon supplying tissue from an archival tissue sample or newly obtained biopsy of a tumor lesion not previously irradiated (tumors progressing in a prior site of radiation are allowed for PD-L1 characterization, other exceptions may be considered after Sponsor consultation). This specimen will be evaluated at a central laboratory for expression status of PD-L1 by immunohistochemistry (IHC). Only subjects with PD-L1 positive tumors will be enrolled in the trial.

This study will be conducted in conformance with Good Clinical Practices.

Specific procedures to be performed during the trial, as well as their prescribed times and associated visit windows, are outlined in the Trial Flow Chart - Section 6.0. Details of each procedure are provided in Section 7.0 – Trial Procedures.

2.2 Trial Diagram

The trial design is depicted in Figure 1.

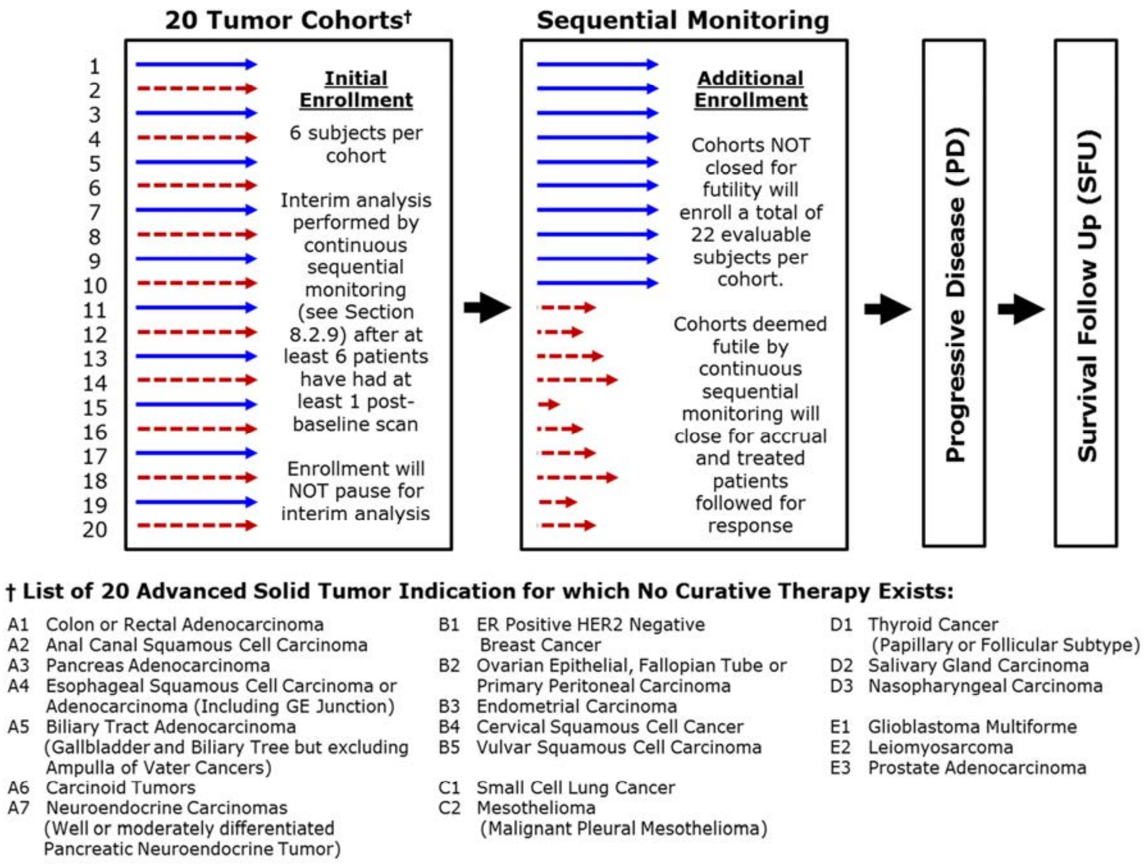

Figure 1 Trial Design

3.0 OBJECTIVE(S) & HYPOTHESIS(ES)

3.1 Primary Objective(s) & Hypothesis(es)

(1) **Objective:** To evaluate preliminary signals of potential anti-tumor activity of pembrolizumab (MK-3475) in subjects with a given histopathologic type of PD-L1 positive advanced solid tumor based on RECIST 1.1 as determined by the investigator in the tumor indications below.

Hypotheses: Intravenous administration of single agent pembrolizumab (MK-3475) to subjects with a given PD-L1 positive solid tumor type will result in a clinically meaningful best overall response rate (ORR) greater than 10% based on RECIST 1.1 criteria.

The primary objective will be tested separately in each tumor indication listed below:

A1 Colon or Rectal Adenocarcinoma
A2 Anal Canal Squamous Cell Carcinoma
A3 Pancreas Adenocarcinoma
A4 Esophageal Squamous Cell Carcinoma or Adenocarcinoma (Including GE Junction)
A5 Biliary Tract Adenocarcinoma (Gallbladder and Biliary Tree but excluding Ampulla of Vater Cancers)
A6 Carcinoid Tumors
A7 Neuroendocrine Carcinomas (Well or moderately differentiated Pancreatic Neuroendocrine Tumor)

B1 ER Positive HER2 Negative Breast Cancer
B2 Ovarian Epithelial, Fallopian Tube or Primary Peritoneal Carcinoma
B3 Endometrial Carcinoma
B4 Cervical Squamous Cell Cancer
B5 Vulvar Squamous Cell Carcinoma

C1 Small Cell Lung Cancer
C2 Mesothelioma (Malignant Pleural Mesothelioma)

D1 Thyroid Cancer (Papillary or Follicular Subtype)
D2 Salivary Gland Carcinoma
D3 Nasopharyngeal Carcinoma

E1 Glioblastoma Multiforme
E2 Leiomyosarcoma
E3 Prostate Adenocarcinoma

3.2 Secondary Objective(s) & Hypothesis(es)

Across-Indication Secondary Objective

(1) **Objective:** To determine the safety and tolerability of pembrolizumab (MK-3475) across selected PD-L1 positive advanced solid tumors.

Within-Indication Secondary Objectives

The following secondary objectives will be evaluated separately in each of the 20 disease indications listed in Section 3.1.

(1) **Objective**: To evaluate the progression-free survival (PFS) in subjects with a given PD-L1 positive advanced solid tumor type receiving pembrolizumab (MK-3475).

(2) **Objective**: To evaluate the overall survival (OS) in subjects with a given PD-L1 positive advanced solid tumor type receiving pembrolizumab (MK-3475).

(3) **Objective**: To evaluate the response duration in subjects with a given PD-L1 positive advanced solid tumor type receiving pembrolizumab (MK-3475).

3.3 Exploratory Objective

Across-Indication Exploratory Objectives

The following exploratory objectives will be evaluated across all 20 disease indications:

(1) **Objective:** To evaluate potential anti-tumor activity of pembrolizumab (MK-3475) in subjects with a given immune signature

(2) **Objective**: To investigate the relationship between other candidate efficacy/resistance biomarkers and anti-tumor activity of pembrolizumab (MK-3475) utilizing tumor tissue and blood sampling.

Within-Indication Exploratory Objectives

The following exploratory objectives will be evaluated separately for each of the 20 disease indications listed in Section 3.1:

(1) **Objective**: To evaluate the anti-tumor activity of pembrolizumab (MK-3475) in subjects with a given PD-L1 positive advanced solid tumor type using continuous tumor measurements.

(2) **Objective**: To explore the relationship between PD-L1 continuous IHC scale score and anti-tumor activity of pembrolizumab (MK-3475) in subjects with a given advanced solid tumor type.

(3) **Objective (optional)**: To evaluate the anti-tumor activity of pembrolizumab (MK-3475) in subjects with a given PD-L1 positive advanced solid tumor type based on RECIST 1.1 as determined by central radiology review, RANO criteria for the glioblastoma multiforme (GBM) cohort, and PCWG2 for the prostate cancer cohort.

4.0 BACKGROUND & RATIONALE

Redacted

Redacted

Redacted

Redacted

Redacted

Redacted

5.0 METHODOLOGY

5.1 Entry Criteria

5.1.1 Diagnosis/Condition for Entry into the Trial

Male/Female subjects with a PD-L1 positive advanced solid tumor (as prespecified in section 2.1) of at least 18 years will be enrolled in this trial.

5.1.2 Subject Inclusion Criteria

In order to be eligible for participation in this trial, the subject must:

1. Be willing and able to provide written informed consent/assent for the trial. The subject may also provide consent/assent for Future Biomedical Research. However, the subject may participate in the main trial without participating in Future Biomedical Research.
2. Be ≥18 years of age on day of signing informed consent.
3. Have histologically or cytologically-documented, locally-advanced, or metastatic solid malignancy that is incurable and has either (a) failed prior standard therapy, (b) for which no standard therapy exists, or (c) standard therapy is not considered appropriate by the patient and treating physician. There is no limit to the number of prior treatment regimens.
4. Have one of the following advanced (unresectable and/or metastatic) solid tumor indications:

   A1 Colon or Rectal Adenocarcinoma
   A2 Anal Canal Squamous Cell Carcinoma
   A3 Pancreas Adenocarcinoma
   A4 Esophageal Squamous Cell Carcinoma or Adenocarcinoma (Including GE Junction)
   A5 Biliary Tract Adenocarcinoma (Gallbladder and Biliary Tree but excluding Ampulla of Vater Cancers)
   A6 Carcinoid Tumors
   A7 Neuroendocrine Carcinomas (Well or moderately differentiated Pancreatic Neuroendocrine Tumor)

   B1 ER Positive HER2 Negative Breast Cancer [a, d]
   B2 Ovarian Epithelial, Fallopian Tube or Primary Peritoneal Carcinoma
   B3 Endometrial Carcinoma [b]
   B4 Cervical Squamous Cell Cancer
   B5 Vulvar Squamous Cell Carcinoma

   C1 Small Cell Lung Cancer
   C2 Mesothelioma (Malignant Pleural Mesothelioma)

D1 Thyroid Cancer (Papillary or Follicular Subtype)
D2 Salivary Gland Carcinoma [b]
D3 Nasopharyngeal Carcinoma

E1 Glioblastoma Multiforme [c]
E2 Leiomyosarcoma
E3 Prostate Adenocarcinoma [d]

[a] Note: *ER positive HER2 negative status for breast cancer cohort defined by local standards.*
[b] Note: *All carcinoma subtypes are allowed however sarcomas or mesenchymal tumors are excluded.*
[c] Note: *Glioblastoma multiforme subjects with any prior bevacizumab treatment are NOT eligible (exceptions may be considered after consultation with the sponsor).*
[d] Note: *Subjects with prostate cancer who are currently on LHRH analogs and breast cancer patients receiving LHRH analogs for ovarian suppression to avoid menses are eligible for this study and may continue to take the LHRH analogs while participating in this study.*

5. Have provided tissue for biomarker analysis from an archival tissue sample or newly obtained core or excisional biopsy of a tumor lesion not previously irradiated (tumors progressing in a prior site of radiation are allowed for PD-L1 characterization, other exceptions may be considered after Sponsor consultation).

6. Have a PD-L1 positive tumor as determined by IHC at a central laboratory from either an archived formalin fixed paraffin embedded (FFPE) tumor sample or a newly obtained biopsy.

7. Have measurable disease based on RECIST 1.1. Tumor lesions situated in a previously irradiated area are considered measurable if progression has been demonstrated in such lesions.

8. Have a performance status of 0 or 1 on the ECOG Performance Scale.

9. Demonstrate adequate organ function as defined in Table 1, all screening labs should be performed within 10 days of treatment initiation.

Table 1 Adequate Organ Function Laboratory Values

System	Laboratory Value
**Hematological**	
Absolute neutrophil count (ANC)	≥1,500 /mcL
Platelets	≥100,000 / mcL
Hemoglobin	≥9 g/dL or ≥5.6 mmol/L
**Renal**	
Creatinine **OR**<br>Measured or calculated[a] creatinine clearance<br>(GFR can also be used in place of creatinine or CrCl)	≤1.5xULN **OR**<br>≥60 mL/min for subject with creatinine levels >1.5x institutional ULN
**Hepatic**	
Total bilirubin	≤1.5xULN **OR**<br>Direct bilirubin ≤ULN for subjects with total bilirubin levels >1.5xULN
AST (SGOT) and ALT (SGPT)	≤2.5xULN **OR**<br>≤5xULN for subjects with liver metastases
**Coagulation**	
International Normalized Ratio (INR) or Prothrombin Time (PT)<br><br>Activated Partial Thromboplastin Time (aPTT)	≤1.5xULN unless subject is receiving anticoagulant therapy as long as PT or PTT is within therapeutic range of intended use of anticoagulants<br>≤1.5xULN unless subject is receiving anticoagulant therapy as long as PT or PTT is within therapeutic range of intended use of anticoagulants
[a] Creatinine clearance should be calculated per institutional standard.	

10. Female subject of childbearing potential should have a negative urine or serum pregnancy test within 72 hours prior to receiving the first dose of study medication. If the urine test is positive or cannot be confirmed as negative, a serum pregnancy test will be required.

11. Female subjects of childbearing potential should be willing to use 2 methods of birth control or be surgically sterile, or abstain from heterosexual activity for the course of the study through 120 days after the last dose of study medication (Reference Section 5.7.2). Subjects of childbearing potential are those who have not been surgically sterilized or have not been free from menses for >1 year.

12. Male subjects should agree to use an adequate method of contraception starting with the first dose of study therapy through 120 days after the last dose of study therapy.

5.1.3 Subject Exclusion Criteria

The subject must be excluded from participating in the trial if the subject:

1. Is currently participating and receiving study therapy or has participated in a study of an investigational agent and received study therapy or used an investigation device within 4 weeks of the first dose of treatment.

2. Has a diagnosis of immunodeficiency or is receiving systemic steroid therapy or any other form of immunosuppressive therapy within 7 days prior to the first dose of trial treatment. The use of physiologic doses of corticosteroids may be approved after consultation with the Sponsor.

   Note: Systemic steroid therapy allowed for subjects in the GBM cohort as long as ≤ dexamethasone 4 mg, or its steroid equivalent (other exceptions may be considered after sponsor consultation).

3. Has had a prior anti-cancer monoclonal antibody (mAb) within 4 weeks prior to study Day 1 or who has not recovered (i.e., ≤ Grade 1 or at baseline) from adverse events due to mAbs administered more than 4 weeks earlier.

4. Has had prior chemotherapy, targeted small molecule therapy, or radiation therapy within 2 weeks prior to study Day 1 or who has not recovered (i.e., ≤ Grade 1 or at baseline) from adverse events due to a previously administered agent.

   Note: Subjects with ≤ Grade 2 neuropathy or ≤ Grade 2 alopecia are an exception to this criterion and may qualify for the study.

   Note: If subject received major surgery, they must have recovered adequately from the toxicity and/or complications from the intervention prior to starting therapy.

   Note: Subjects in the GBM cohort are not eligible if they have had radiation therapy to their only sites of measurable CNS disease within 12 weeks prior to study Day 1.

5. Has a known additional malignancy that is progressing or requires active treatment. Exceptions include basal cell carcinoma of the skin, squamous cell carcinoma of the skin that has undergone potentially curative therapy or in situ cervical cancer.

6. Has known active central nervous system (CNS) metastases and/or carcinomatous meningitis. Subjects with previously treated brain metastases may participate provided they are stable (without evidence of progression by imaging for at least four weeks prior to the first dose of trial treatment and any neurologic symptoms have returned to baseline), have no evidence of new or enlarging brain metastases, and are not using steroids for at least 7 days prior to trial treatment. This exception does not include carcinomatous meningitis which is excluded regardless of clinical stability.

7. Has an active autoimmune disease that has required systemic treatment in past 2 years (i.e. with use of disease modifying agents, corticosteroids or immunosuppressive drugs). Replacement therapy (e.g., thyroxine, insulin, or physiologic corticosteroid replacement therapy for adrenal or pituitary insufficiency, etc.) is not considered a form of systemic treatment.

8. Has evidence of interstitial lung disease.

9. Has an active infection requiring systemic therapy.

10. Has a history or current evidence of any condition, therapy, or laboratory abnormality that might confound the results of the trial, interfere with the subject's participation for the full duration of the trial, or is not in the best interest of the subject to participate, in the opinion of the treating investigator.

11. Has known psychiatric or substance abuse disorders that would interfere with cooperation with the requirements of the trial.

12. Is pregnant or breastfeeding, or expecting to conceive or father children within the projected duration of the trial, starting with the pre-screening or screening visit through 120 days after the last dose of trial treatment.

13. Has previously participated in any other pembrolizumab (MK-3475) trial, or received prior therapy with an anti-PD-1, anti-PD-L1, and anti-PD-L2 (including ipilimumab or any other antibody or drug specifically targeting T-cell co-stimulation or checkpoint pathways).

14. Has a known history of Human Immunodeficiency Virus (HIV) (HIV 1/2 antibodies).

15. Has known active Hepatitis B (e.g., HBsAg reactive) or Hepatitis C (e.g., HCV RNA [qualitative] is detected).

16. Has received a live vaccine within 30 days of planned start of study therapy.

17. Is or has an immediate family member (spouse or children) who is investigational site or sponsor staff directly involved with this trial, unless prospective IRB approval (by chair or designee) is given allowing exception to this criterion for a specific patient.

5.2 Trial Treatment(s)

The study drug dose and schedule to be used in this trial are outlined below in Table 2.

Table 2 Trial Treatment

Drug	Dose/Potency	Dose Frequency	Route of Administration	Regimen/ Treatment Period	Use
pembrolizumab (MK-3475)	10 mg/kg	Q2W	IV infusion	Day 1 of each cycle	Experimental
The pembrolizumab (MK-3475) dosing interval may be withheld due to toxicity as described in Section 5.2.1.2.					

Trial treatment should begin on the day of randomization or as close as possible to the date on which the subject is allocated/assigned.

The investigator shall take responsibility for and shall take all steps to maintain appropriate records and ensure appropriate supply, storage, handling, distribution and usage of trial treatments in accordance with the protocol and any applicable laws and regulations.

5.2.1 Dose Selection/Modification

5.2.1.1 Dose Selection (Preparation)

The rationale for selection of doses to be used in this trial is provided in Section 4.0 – Background and Rationale.

The dose amount required to prepare the pembrolizumab (MK-3475) infusion solution will be based on the subject’s weight in kilograms (kg). Details on the dose calculation, preparation and administration are provided in the Pharmacy Manual.

5.2.1.2 Dose Modification

For subjects whose dose was withheld due to toxicity, subjects may resume pembrolizumab (MK-3475) upon resolution of toxicity to Grade 0-1 or baseline. This dose would be considered Day 1 of the next cycle and should be in alignment with the new schedule.

Table 3 Dose Modification Guidelines for Drug-Related Adverse Events

Toxicity	Hold Treatment For Grade	Timing for Restarting Treatment	Discontinue Subject
Diarrhea/Colitis	2-3[1]	Toxicity resolves to Grade 0-1.	Toxicity does not resolve within 12 weeks of last dose or inability to reduce corticosteroid to 10 mg or less of prednisone or equivalent per day within 12 weeks.
	4[1]	Permanently discontinue	Permanently discontinue
Hypopysitis	2-3[1]	Toxicity resolves to Grade 0-1.	Toxicity does not resolve within 12 weeks of last dose or inability to reduce corticosteroid to 10 mg or less of prednisone or equivalent per day within 12 weeks.
	4[1]	Toxicity resolves to Grade 0-1	Toxicity does not resolve within 12 weeks of last dose or inability to reduce corticosteroid to 10 mg or less of prednisone or equivalent per day within 12 weeks.
AST, ALT, or Increased Bilirubin	2[1]	Toxicity resolves to Grade 0-1	Toxicity does not resolve within 12 weeks of last dose.
	3-4[1]	Permanently discontinue (see exception below)[2]	Permanently discontinue
Hyperthyroidism or Hypothyroidism	3[1]	Toxicity resolves to Grade 0-1	Toxicity does not resolve within 12 weeks of last dose or inability to reduce corticosteroid to 10 mg or less of prednisone or equivalent per day within 12 weeks.
	4[1]	Permanently discontinue	Permanently discontinue
Infusion Reaction	3-4	Permanently discontinue	Permanently discontinue
Pneumonitis	2[1]	Toxicity resolves to Grade 0-1	Toxicity does not resolve within 12 weeks of last dose or inability to reduce corticosteroid to 10 mg or less of prednisone or equivalent per day within 12 weeks.
	3-4[1]	Permanently discontinue	Permanently discontinue
Renal Failure or Nephritis	2[1]	Toxicity resolves to Grade 0-1	Toxicity does not resolve within 12 weeks of last dose or inability to reduce corticosteroid to 10 mg or less of prednisone or equivalent per day within 12 weeks.
	3-4[1]	Permanently discontinue	Permanently discontinue
All Other Drug-Related Toxicity[3]	3 or Severe[1]	Toxicity resolves to Grade 0-1	Toxicity does not resolve within 12 weeks of last dose or inability to reduce corticosteroid to 10 mg or less of prednisone or equivalent per day within 12 weeks.
	4	Permanently discontinue	Permanently discontinue

Note: Permanently discontinue for any severe or Grade 3 drug-related AE that recurs or any life-threatening event.

[1] *Administer corticosteroid (See also Events of Clinical Interest Guidance Document and Section 7.2.3.2 Events of Clinical Interest.*

[2] *For patients with liver metastasis who begin treatment with Grade 2 AST or ALT, if AST or ALT increases by greater than or equal to 50% relative to baseline and lasts for at least 1 week then patients should be discontinued.*

[3] Patients with intolerable or persistent Grade 2 drug-related AE may hold study medication at physician discretion. Permanently discontinue study drug for persistent Grade 2 adverse reactions for which treatment with study drug has been held, that do not recover to Grade 0-1 within 12 weeks of the last dose.

In case toxicity related to pembrolizumab (MK-3475) does not resolve to Grade 0-1 within 12 weeks after last infusion, trial treatment should be discontinued. Subjects with a laboratory adverse event still at Grade 2 after 12 weeks may continue treatment in the trial only if asymptomatic and controlled. For information on the management of adverse events, see Section 5.6.1.

5.2.2 Timing of Dose Administration

Study drug should be administered on Day 1 of each cycle after all procedures/assessments have been completed as detailed on the Trial Flow Chart (Section 6.0). Study drug may be administered up to 3 days before or after the scheduled Day 1 of each cycle due to administrative reasons.

All study drug will be administered on an outpatient basis.

Pembrolizumab (MK-3475) will be administered as a 30 minute IV infusion every 2 weeks (treatment cycle intervals may be withheld due to toxicity as described in Section 5.2.1.2). Sites should make every effort to target infusion timing to be as close to 30 minutes as possible. However, given the variability of infusion pumps from site to site, a window of -5 minutes and +10 minutes is permitted (i.e., infusion time is 30 minutes: -5 min/+10 min).

The Pharmacy Manual contains specific instructions for pembrolizumab (MK-3475) dose calculation, preparation of the infusion solution, and administration.

Continuing Study Drug Administration After Initial Evidence of Radiologic Disease Progression

Immunotherapeutic agents such as pembrolizumab (MK-3475) may produce antitumor effects by potentiating endogenous cancer-specific immune responses. The response patterns seen with such an approach may extend beyond the typical time course of responses seen with cytotoxic agents, and can manifest as a clinical response after an initial increase in tumor burden or even the appearance of new lesions.

If initial radiologic imaging shows PD, tumor assessment should be repeated ≥4 weeks later in order to confirm PD with the option of continuing treatment per below while awaiting radiologic confirmation of progression. If repeat imaging shows a reduction in the tumor burden compared to the initial scan demonstrating PD, treatment may be continued as per treatment calendar. If repeat imaging confirms progressive disease, subjects will be discontinued from study therapy (exception noted in Section 7.1.2.5.1). In determining whether or not the tumor burden has increased or decreased, Investigators should consider all target lesions as well as non-target lesions (please refer to the Procedures Manual).

When feasible, subjects should not be discontinued until progression is confirmed; however, the decision to continue study treatment after the 1st evidence of disease progression is at the Investigator's discretion based on the clinical status of the subject as described in Table 4 below. Subjects may receive study treatment while waiting for confirmation of PD if they are clinically stable as defined by the following criteria:

- Absence of signs and symptoms (including worsening of laboratory values) indicating disease progression
- No decline in ECOG performance status
- Absence of rapid progression of disease
- Absence of progressive tumor at critical anatomical sites (e.g., cord compression) requiring urgent alternative medical intervention

Table 4 Imaging and Treatment After 1st Radiologic Evidence of PD

	Clinically Stable		Clinically Unstable	
	Imaging	Treatment	Imaging	Treatment
1st radiologic evidence of PD	Repeat imaging at ≥4 weeks to confirm PD	May continue study treatment at the Investigator's discretion while awaiting confirmatory scan	Repeat imaging at ≥4 weeks to confirm PD if possible	Discontinue treatment
Repeat scan confirms PD	No additional imaging required	Discontinue treatment (exception noted in Section 7.1.2.5.1)	No additional imaging required	N/A
Repeat scan shows SD, PR or CR	Continue regularly scheduled imaging assessments every 8 weeks (after 6 months, imaging assessments every 12 weeks)	Continue study treatment at the Investigator's discretion	Continue regularly scheduled imaging assessments every 8 weeks (after 6 months, imaging assessments every 12 weeks)	May restart study treatment if condition has improved and/or clinically stable per Investigator's discretion

5.2.3 Trial Blinding/Masking

This is an open-label trial; therefore, the Sponsor, investigator and subject will know the treatment administered.

5.3 Randomization or Treatment Allocation

Subjects participating in this trial will be allocated by non-random assignment.

5.4 Stratification

No stratification based on age, sex or other characteristics will be used in this trial.

5.5 Concomitant Medications/Vaccinations (Allowed & Prohibited)

Medications or vaccinations specifically prohibited in the exclusion criteria are not allowed during the ongoing trial. If there is a clinical indication for any medication or vaccination specifically prohibited during the trial, discontinuation from trial therapy or vaccination may be required. The investigator should discuss any questions regarding this with the Sponsor Clinical Director. The final decision on any supportive therapy or vaccination rests with the investigator and/or the subject's primary physician. However, the decision to continue the subject on trial therapy or vaccination schedule requires the mutual agreement of the investigator, the Sponsor and the subject.

5.5.1 Acceptable Concomitant Medications

All treatments that the investigator considers necessary for a subject's welfare may be administered at the discretion of the investigator in keeping with the community standards of medical care. All concomitant medication will be recorded on the case report form (CRF) including all prescription, over-the-counter (OTC), herbal supplements, and IV medications and fluids. If changes occur during the trial period, documentation of drug dosage, frequency, route, and date may also be included on the CRF.

All concomitant medications received within 28 days before the first dose of trial treatment and 30 days after the last dose of trial treatment should be recorded. Concomitant medications administered after 30 days after the last dose of trial treatment should be recorded for SAEs and ECIs as defined in Section 7.2.

5.5.2 Prohibited Concomitant Medications

Subjects are prohibited from receiving the following therapies during the Screening and Treatment Phase (including retreatment for post-complete response relapse) of this trial:

- Anti-cancer systemic chemotherapy or biological therapy

  Note: Subjects with glioblastoma multiforme with any prior bevacizumab treatment are not eligible (exceptions may be considered after consultation with the sponsor).

  Note: Subjects with prostate cancer who are currently on LHRH analogs and breast cancer patients receiving LHRH analogs for ovarian suppression to avoid menses are eligible for this study and may continue to take the LHRH analogs while participating in this study.

- Immunotherapy not specified in this protocol
- Chemotherapy not specified in this protocol
- Investigational agents other than pembrolizumab (MK-3475)
- Radiation therapy

  Note: Radiation therapy to a symptomatic solitary lesion or to the brain may be allowed after consultation with Sponsor.

- Live vaccines within 30 days prior to the first dose of trial treatment and while participating in the trial. Examples of live vaccines include, but are not limited to, the following: measles, mumps, rubella, chicken pox, yellow fever, rabies, BCG, and typhoid (oral) vaccine. Seasonal influenza vaccines for injection are generally killed virus vaccines and are allowed; however intranasal influenza vaccines (e.g. Flu-Mist®) are live attenuated vaccines, and are not allowed

- Glucocorticoids for any purpose other than to modulate symptoms from an adverse event.

  Note: The use of physiologic doses (e.g., prednisone 10 mg) of corticosteroids may be approved after consultation with the Sponsor. Concomitant administration of higher steroid doses with study medication is prohibited.

  Note: Systemic steroid therapy allowed for subjects in the GBM cohort as long as ≤ dexamethasone 4 mg, or its steroid equivalent (other exceptions may be considered after sponsor consultation).

  Note: Use of prophylactic corticosteroids to avoid allergic reactions (e.g. IV contrast dye) is permitted.

Subjects who, in the assessment by the investigator, require the use of any of the aforementioned treatments for clinical management should be removed from the trial. Subjects may receive other medications that the investigator deems to be medically necessary.

The Exclusion Criteria describes other medications which are prohibited in this trial.

There are no prohibited therapies during the Post-Treatment Follow-up Phase.

5.6 Rescue Medications & Supportive Care

5.6.1 Supportive Care Guidelines

Subjects should receive appropriate supportive care measures as deemed necessary by the treating investigator including but not limited to the items outlined below:

- Diarrhea: Subjects should be carefully monitored for signs and symptoms of enterocolitis (such as diarrhea, abdominal pain, blood or mucus in stool, with or without fever) and of bowel perforation (such as peritoneal signs and ileus). In symptomatic subjects, infectious etiologies should be ruled out, and if symptoms are persistent and/or severe, endoscopic evaluation should be considered.

  - All subjects who experience diarrhea should be advised to drink liberal quantities of clear fluids. If sufficient oral fluid intake is not feasible, fluid and electrolytes should be substituted via IV infusion.

- Nausea/vomiting: Nausea and vomiting should be treated aggressively, and consideration should be given in subsequent cycles to the administration of prophylactic antiemetic therapy according to standard institutional practice. Subjects should be strongly encouraged to maintain liberal oral fluid intake.

- Anti-infectives: Subjects with a documented infectious complication should receive oral or IV antibiotics or other anti-infective agents as considered appropriate by the treating investigator for a given infectious condition, according to standard institutional practice.

- Immune-related adverse events: Please see Section 5.6.1.1 below and the separate guidance document in the administrative binder regarding diagnosis and management of adverse experiences of a potential immunologic etiology.

- Management of Infusion Reactions: Acute infusion reactions (which can include cytokine release syndrome, angioedema, or anaphylaxis) are different from allergic/hypersensitive reactions, although some of the manifestations are common to both AEs. Signs and symptoms usually develop during or shortly after drug infusion and generally resolve completely within 24 hours of completion of infusion. Signs/symptoms may include: Allergic reaction/hypersensitivity (including drug fever); Arthralgia (joint pain); Bronchospasm; Cough; Dizziness; Dyspnea (shortness of breath); Fatigue (asthenia, lethargy, malaise); Headache; Hypertension; Hypotension; Myalgia (muscle pain); Nausea; Pruritus/itching; Rash/desquamation; Rigors/chills; Sweating (diaphoresis); Tachycardia; Tumor pain (onset or exacerbation of tumor pain due to treatment); Urticaria (hives, welts, wheals); Vomiting.

Table 5 below shows treatment guidelines for subjects who experience an infusion reaction associated with administration of pembrolizumab (MK-3475).

Table 5 Infusion Reaction Treatment Guidelines

NCI CTCAE Grade	Treatment	Premedication at Subsequent Dosing
Grade 1<br>Mild reaction; infusion interruption not indicated; intervention not indicated	Increase monitoring of vital signs as medically indicated until the subject is deemed medically stable in the opinion of the investigator.	None
Grade 2<br>Requires infusion interruption but responds promptly to symptomatic treatment (e.g., antihistamines, NSAIDS, narcotics, IV fluids); prophylactic medications indicated for < =24 hrs	**Stop Infusion and monitor symptoms.**<br>Additional appropriate medical therapy may include but is not limited to:<br>IV fluids<br>Antihistamines<br>NSAIDS<br>Acetaminophen<br>Narcotics<br>Increase monitoring of vital signs as medically indicated until the subject is deemed medically stable in the opinion of the investigator.<br>If symptoms resolve within one hour of stopping drug infusion, the infusion may be restarted at 50% of the original infusion rate (e.g., from 100 mL/hr to 50 mL/hr). Otherwise dosing will be held until symptoms resolve and the subject should be premedicated for the next scheduled dose.<br>**Subjects who develop Grade 2 toxicity despite adequate premedication should be permanently discontinued from further trial treatment administration.**	Subject may be premedicated 1.5h (± 30 minutes) prior to infusion of pembrolizumab (MK-3475) with:<br><br>Diphenhydramine 50 mg po (or equivalent dose of antihistamine).<br><br>Acetaminophen 500-1000 mg po (or equivalent dose of antipyretic).
Grades 3 or 4<br><br>Grade 3:<br>Prolonged (i.e., not rapidly responsive to symptomatic medication and/or brief interruption of infusion); recurrence of symptoms following initial improvement; hospitalization indicated for other clinical sequelae (e.g., renal impairment, pulmonary infiltrates)<br><br>Grade 4:<br>Life-threatening; pressor or ventilatory support indicated	**Stop Infusion.**<br>Additional appropriate medical therapy may include but is not limited to:<br>IV fluids<br>Antihistamines<br>NSAIDS<br>Acetaminophen<br>Narcotics<br>Oxygen<br>Pressors<br>Corticosteroids<br>Epinephrine<br>Increase monitoring of vital signs as medically indicated until the subject is deemed medically stable in the opinion of the investigator.<br>Hospitalization may be indicated.<br>**Subject is permanently discontinued from further trial treatment administration.**	No subsequent dosing
Appropriate resuscitation equipment should be available in the room and a physician readily available during the period of drug administration.<br>For Further information, please refer to the Common Terminology Criteria for Adverse Events v4.0 (CTCAE) at http://ctep.cancer.gov		

5.6.1.1 Supportive Care Guidelines for Events of Clinical Interest and Immune-related Adverse Events (irAEs)

Immune-related Adverse Events (irAE)

Adverse events (both non-serious and serious) associated with drug exposure and consistent with an immune phenomenon may represent an immunologic etiology. These immune related adverse events (irAEs) may be predicted based on the nature of the pembrolizumab compound, its mechanism of action, and reported experience with immunotherapies that have a similar mechanism of action. An irAE can occur shortly after the first dose or several months after the last dose of treatment. Particular attention should be paid to AEs that may be suggestive of potential irAEs as outlined in 7.2.3.2. Information on how to identify and evaluate irAEs has been developed and is included in the Event of Clinical Interest Guidance Document located in the Administrative Binder.

Events of Clinical Interest (ECI)

Events of clinical interest (ECI) are non-serious and serious adverse events that may or may not be irAEs. ECIs must be reported to Merck **within 24 hours** regardless of attribution to study treatment. Information on how to identify and report ECIs can be referenced in both Section 7.2.3.2 and the Event of Clinical Interest Guidance Document.

5.6.1.2 Supportive Care Guidelines for Pneumonitis

Subjects with symptomatic pneumonitis should immediately stop receiving pembrolizumab (MK-3475) and have an evaluation. The evaluation may include bronchoscopy and pulmonary function tests to rule out other causes such as infection. If the subject is determined to have study drug associated pneumonitis, the suggested treatment plan is detailed in the Event of Clinical Interest Guidance Document located in the Administrative Binder.

5.7 Diet/Activity/Other Considerations

5.7.1 Diet

Subjects should maintain a normal diet unless modifications are required to manage an AE such as diarrhea, nausea or vomiting.

5.7.2 Contraception

Pembrolizumab (MK-3475) may have adverse effects on a fetus in utero. Furthermore, it is not known if MK 3475 has transient adverse effects on the composition of sperm. Non-pregnant, non-breast-feeding women may be enrolled if they are willing to use 2 methods of birth control or are considered highly unlikely to conceive. Highly unlikely to conceive is defined as 1) surgically sterilized, or 2) postmenopausal (a woman who is ≥45 years of age and has not had menses for greater than 1 year will be considered postmenopausal), or 3) not heterosexually active for the duration of the study. The two birth control methods can be

either two barrier methods or a barrier method plus a hormonal method to prevent pregnancy. Subjects should start using birth control from study Visit 1 throughout the study period up to 120 days after the last dose of study therapy.

The following are considered adequate barrier methods of contraception: diaphragm, condom (by the partner), copper intrauterine device, sponge, or spermicide, as per local regulations or guidelines. Appropriate hormonal contraceptives will include any registered and marketed contraceptive agent that contains an estrogen and/or a progestational agent (including oral, subcutaneous, intrauterine, or intramuscular agents).

Subjects should be informed that taking the study medication may involve unknown risks to the fetus (unborn baby) if pregnancy were to occur during the study. In order to participate in the study they must adhere to the contraception requirement (described above) for the duration of the study and during the follow-up period defined in section 7.2.2 - Reporting of Pregnancy and Lactation to the Sponsor. If there is any question that a subject will not reliably comply with the requirements for contraception, that subject should not be entered into the study.

5.7.3 Use in Pregnancy

If a subject inadvertently becomes pregnant while on treatment with pembrolizumab (MK-3475), the subject will immediately be removed from the study. The site will contact the subject at least monthly and document the subject's status until the pregnancy has been completed or terminated. The outcome of the pregnancy will be reported to the Sponsor without delay and within 24 hours if the outcome is a serious adverse experience (e.g., death, abortion, congenital anomaly, or other disabling or life-threatening complication to the mother or newborn). The study investigator will make every effort to obtain permission to follow the outcome of the pregnancy and report the condition of the fetus or newborn to the Sponsor. If a male subject impregnates his female partner the study personnel at the site must be informed immediately and the pregnancy reported to the Sponsor and followed as described above and in Section 7.2.2.

5.7.4 Use in Nursing Women

It is unknown whether MK 3475 is excreted in human milk. Since many drugs are excreted in human milk, and because of the potential for serious adverse reactions in the nursing infant, subjects who are breast-feeding are not eligible for enrollment.

5.8 Subject Withdrawal/Discontinuation Criteria

Subjects may withdraw consent at any time for any reason or be dropped from the trial at the discretion of the investigator should any untoward effect occur. In addition, a subject may be withdrawn by the investigator or the Sponsor if enrollment into the trial is inappropriate, the trial plan is violated, or for administrative and/or other safety reasons. Specific details regarding discontinuation or withdrawal procedures; including specific details regarding

withdrawal from Future Biomedical Research, are provided in Section 7.1.4 – Other Procedures.

A subject must be discontinued from the trial for any of the following reasons:

- The subject or legal representative (such as a parent or legal guardian) withdraws consent.

A subject must be discontinued from treatment (but may continue to be monitored in the trial) for any of the following reasons:

- The subject or legal representative (such as a parent or legal guardian) withdraws consent for treatment
- Confirmed radiographic disease progression

  Note: For unconfirmed radiographic disease progression, please see Section 5.2.2

  Note: A subject may be granted an exception to continue on treatment with confirmed radiographic progression if clinically stable or clinically improved, please see Section 7.1.2.5.1
- Unacceptable adverse experiences as described in Section 5.2.1.2
- Intercurrent illness that prevents further administration of treatment
- Investigator's decision to withdraw the subject
- The subject has a confirmed positive serum pregnancy test
- Noncompliance with trial treatment or procedure requirements
- The subject is lost to follow-up
- Completed 24 months of treatment with pembrolizumab (MK-3475)

  Note: 24 months of study medication is calculated from the date of first dose. Subjects who stop pembrolizumab (MK-3475) after 24 months may be eligible for up to one year of additional study treatment if they progress after stopping study treatment provided they meet the requirements detailed in Section 7.1.5.2.1.
- Administrative reasons

The End of Treatment and Follow-up visit procedures are listed in Section 6 (Protocol Flow Chart) and Section 7.1.5 (Visit Requirements). After the end of treatment, each subject will be followed for 30 days for adverse event monitoring (serious adverse events will be collected for 90 days after the end of treatment as described in Section 7.2.3.1). Subjects

who discontinue for reasons other than progressive disease will have post-treatment follow-up for disease status until disease progression, initiating a non-study cancer treatment, withdrawing consent or becoming lost to follow-up. After documented disease progression each subject will be followed by telephone for overall survival until death, withdrawal of consent, or the end of the study, whichever occurs first.

5.8.1 Discontinuation of Study Therapy after CR

Discontinuation of treatment may be considered for subjects who have attained a confirmed CR that have been treated for at least 24 weeks with pembrolizumab (MK-3475) and had at least two treatments with pembrolizumab (MK-3475) beyond the date when the initial CR was declared. Subjects who then experience radiographic disease progression may be eligible for up to one year of additional treatment with pembrolizumab (MK-3475) at the discretion of the investigator if no cancer treatment was administered since the last dose of pembrolizumab (MK-3475), the subject meets the safety parameters listed in the Inclusion/Exclusion criteria, and the trial is open. Subjects will resume therapy at the same dose and schedule at the time of initial discontinuation. Additional details are provided in Section 7.1.5.2.1.

5.9 Subject Replacement Strategy

Additional subjects may be enrolled in a given cohort to ensure that the required number of evaluable subjects in each cohort is achieved. A subject that discontinues the trial for progressive disease or a drug-related AE will not be replaced and will be counted in the evaluable population of subjects for the respective cohort. Further details are provided in Section 8.1.3.

5.10 Beginning and End of the Trial

The study begins when the first subject signs the informed consent (either pre-screening consent or main study consent). The end of the study may be designated as the time point when all subjects have discontinued the study or are a minimum of 6 months post initial study medication administration. If, by the end of the study, there remains at least 1 subject still on study treatment for at least 6 months, the subject(s) may enter additional treatment cycles. At this point a database lock of the trial may occur to allow the analysis of the study data. Any remaining subjects may continue to receive study medication and be seen by the investigator per usual standard of care for this subject population. In addition, the investigator will be expected to monitor for and report any serious adverse events, events of clinical interest, and pregnancies, as detailed in Section 7.2.3 (Serious Adverse Experiences). The subject is considered on study until such time that he/she meets any of the discontinuation criteria and written notification is given to the Sponsor.

5.11 Clinical Criteria for Early Trial Termination

Early trial termination will be the result of the criteria specified below:

1. Quality or quantity of data recording is inaccurate or incomplete

2. Poor adherence to protocol and regulatory requirements
3. Incidence or severity of adverse drug reaction in this or other studies indicates a potential health hazard to subjects
4. Plans to modify or discontinue the development of the study drug

In the event of Sponsor decision to no longer supply study drug, ample notification will be provided so that appropriate adjustments to subject treatment can be made.

6.0 TRIAL FLOW CHART

6.1 Study Flow Chart

**Trial Period:**	**Screening Phase**	**Treatment Cycles [a]**								**End of Treatment**	**Post-Treatment**		
						To be repeated beyond 8 cycles [a]							
Treatment Cycle/Title:	Screening (Visit 1)	1	2	3	4	5	6	7	8	Discon	Safety Follow-up	Follow Up Visits [b]	Survival Follow-Up
Scheduling Window (Days):	-28 to -1		± 3	± 3	± 3	± 3	± 3	± 3	± 3	At time of Discon	30 days from last dose (± 3 days)	Every 8 weeks post discon (± 7 days)	Every 12 weeks (± 4 weeks)
**Administrative Procedures**													
Pre-screening Consent	X[c]												
Informed Consent	X[d]												
Informed Consent for Future Biomedical Research (optional)	X												
Inclusion/Exclusion Criteria	X												
Subject Identification Card	X												
Demographics and Medical History	X												
Prior and Concomitant Medication Review	X[f]	X	X	X	X	X	X	X	X	X	X[f]		
Trial Treatment Administration		X	X	X	X	X	X	X	X				
Post-study Anticancer Therapy Status												X	X
Survival Status													X
**Clinical Procedures/Assessments**													
Review Adverse Events	X	X	X	X	X	X	X	X	X	X	X[g]	X[g]	
Full Physical Examination	X					X[h]							
Directed Physical Examination		X	X	X	X		X[u]	X[u]	X[u]	X			
Vital Signs and Weight [i]	X	X	X	X	X	X	X	X	X	X			
ECOG Performance Status	X	X	X	X	X	X	X[v]	X[v]	X[v]	X			

**Trial Period:**	**Screening Phase**	**Treatment Cycles [a]**								**End of Treatment**	**Post-Treatment**		
						To be repeated beyond 8 cycles [a]							
Treatment Cycle/Title:	Screening (Visit 1)	1	2	3	4	5	6	7	8	Discon	Safety Follow-up	Follow Up Visits [b]	Survival Follow-Up
Scheduling Window (Days):	-28 to -1		± 3	± 3	± 3	± 3	± 3	± 3	± 3	At time of Discon	30 days from last dose (± 3 days)	Every 8 weeks post discon (± 7 days)	Every 12 weeks (± 4 weeks)
**Laboratory Procedures/Assessments: analysis performed by LOCAL laboratory**													
Pregnancy Test – Urine or Serum β-HCG[k]	X												
PT/INR and aPTT	X[l]												
CBC with Differential[m]	X[l]		X	X	X	X	X	X	X	X	X[n]		
Comprehensive Chemistry Panel[m]	X[l]		X	X	X	X	X	X	X	X	X[n]		
Urinalysis[m]	X[l]					X[h]					X[n]		
T3, FT4 and TSH[m]	X[l]					X[h]					X[n]		
Tumor Marker Assessment (Optional) [j]	X [j]					X [j]				X [j]		X [j]	
**Laboratory Procedures/Assessments: analysis performed by CENTRAL laboratory**													
Blood for Future Biomedical Research[o]		X											
**Efficacy Measurements**													
Tumor Imaging [a,p,q]	X					X				X[r]		X[b]	
**Tumor Biopsies/Archival Tissue Collection/Correlative Studies Blood**													
Archival or Newly Obtained Tissue Collection [e]	X[S]												
Correlative Studies Blood Collection [e]		X[t]				X[t]				X[t]			

a. Treatment cycles are 2 weeks. Imaging should be performed every 8 weeks initially (56 days ± 7 days) and after 6 months, imaging assessments should be performed every 12 weeks regardless of any treatment delays (i.e. Screening visit, Cycle 5, Cycle 9, Cycle 13 and then every 6 cycles (12 weeks)).
b. In subjects who discontinue study therapy without documented disease progression, every effort should be made to continue monitoring their disease status by radiologic imaging[p] every 8 weeks (± 7 days) until (1) the start of new anti-cancer treatment, (2) documented disease progression, (3) death, or (4) the end of the study, whichever occurs first.
c. Pre-screening informed consent must be obtained prior to sending an archival sample to the vendor for characterization. Subjects that do not have archival tissue available must sign the main study consent prior to undergoing a newly obtained biopsy.
d. Written consent must be obtained prior to performing any protocol specific procedure. Results of a test performed prior to the subject signing consent as part of routine clinical management are acceptable in lieu of a screening test if performed within the specified time frame (e.g., within 28 days prior to the first dose of trial treatment). Baseline number will be assigned when the study informed consent is signed.
e. Leftover samples may be kept for Future Biomedical Research if the subject signs the FBR consent.
f. Prior medications – Record all medications taken 28 days before the first dose of trial treatment. Concomitant medications – Enter new medications started during the trial and 30 days after last dose of trial treatment regardless of when the Safety Follow-up visit occurs.
g. Record all AEs occurring within 30 days after the last dose of trial treatment regardless of when the Safety Follow-up visit occurs. After 30 days, record all SAEs (related and unrelated to trial treatment) / ECIs occurring up to 90 days after the last dose of trial treatment or the start of new anti-cancer treatment, whichever comes first. Afterwards, any drug related AE regardless of seriousness occurring outside of any reporting timeframes must be reported
h. To be repeated every 4 cycles after Cycle 5.
i. Height will be measured at visit 1 only.
j. Tumor marker assessment is not an additional study-related laboratory evaluation. The purpose is to collect information that may be a part of standard clinical assessment for certain tumor types. Data collection (if applicable and if available) should occur every 8 weeks initially (56 days ± 7 days) and after 6 months, assessments should be performed every 12 weeks regardless of any treatment delays (i.e. Screening visit, Cycle 5, Cycle 9, Cycle 13 and then every 6 cycles (12 weeks) thereafter coinciding with imaging visits). Upon discontinuation, tumor marker data collection will occur every 8 weeks coinciding with post-treatment imaging follow-up visits. See Section 7.1.2.7 and Section 7.1.3.1 for additional details.
k. For women of reproductive potential, a urine pregnancy test should be performed within 72 hours prior to first dose of trial treatment. If urine pregnancy results cannot be confirmed as negative, a serum pregnancy test performed by the local study site laboratory will be required. Pregnancy tests (serum and/or urine tests) should be repeated if required by local guidelines.
l. Laboratory tests for screening are to be performed within 10 days prior to the first dose of trial treatment. See Section 7.1.3 for details regarding laboratory tests.
m. After Cycle 1, lab samples can be collected up to 72 hours prior to the scheduled time point. See Section 7.1.3 for details regarding laboratory tests.
n. Unresolved abnormal labs that are drug related AEs should be followed until resolution.
o. Informed consent for future biomedical research samples must be obtained before the DNA sample. DNA sample for analysis should be obtained predose, on Day 1 (or with the next scheduled blood draw) as the last sample drawn or at a later date as soon as the informed consent is obtained.
p. The initial tumor imaging will be performed within 28 days prior to the first dose of trial treatment. Scans performed as part of routine clinical management are acceptable for use as the screening scan if they are of diagnostic quality and performed within 28 days prior to the first dose of trial treatment. On-study imaging will be performed every 8 weeks (± 7 days) after the first dose of trial treatment (after 6 months, imaging assessments every 12 weeks) and should follow calendar days and should not be adjusted for delays in cycle starts or withholding of pembrolizumab (MK-3475) cycle frequencies. The same imaging technique should be used in a subject throughout the trial. Local reading (investigator assessment with site radiology reading) will be used to determine eligibility and for subject management. Tumor imaging and assessment per local standard of care should be performed for patient management,

and may include additional imaging (e.g. bone scan for prostate cancer patients) and appropriate tumor markers. Sponsor will collect radiological assessments for retrospective analysis by a central vendor. The processes for image collection and transmission to the central vendor are in the Site Imaging Manual (SIM).

q. Per the modified RECIST 1.1 used in this protocol, if imaging shows progressive disease, the imaging assessment should be repeated at a minimum of 4 weeks later in order to confirm progressive disease as described in Section 4.2.3.1.
r. If a scan was obtained within 4 weeks prior to the date of discontinuation, then a scan at treatment discontinuation isn't mandatory. In subjects who discontinue study therapy without confirmed disease progression, a radiological evaluation should be repeated at the time of treatment discontinuation (i.e. date of discontinuation ± 4 week window).
s. Baseline tumor tissue for biomarker analysis from an archival tissue sample or newly obtained core or excisional biopsy (FNA not adequate) of a tumor lesion not previously irradiated must be provided (tumors progressing in a prior site of radiation are allowed for PD-L1 characterization, other exceptions may be considered after Sponsor consultation) and received by the central vendor for characterization of PD-L1 status prior to enrollment. These samples are not required to be obtained within 28 days of enrollment.
t. Blood for correlative studies should be collected prior to Cycle 1, at Cycle 5 and again at discontinuation (end of treatment).
u. Directed physical exams are performed at Cycles 1, 2, 3, 4, 6, 7, 8 and 11. After Cycle 11, directed physical exams should occur once every 4 cycles (Cycles 15, 19, etc.).
v. Following Cycle 8, the ECOG performance status should be determined only in conjunction with a protocol-specified full or directed physical exam (Cycle 9, 11, 13, 15, 17, 19 and every 2 cycles thereafter.

6.2 Second Course Phase (Retreatment ONLY)

**Trial Period:**	**SECOND COURSE PHASE: Treatment Cycles [a]**								**End of Treatment**	**Post-Treatment**		
					To be repeated beyond 8 cycles [a]							
Treatment Cycle/Title:	1	2	3	4	5	6	7	8	Discon	Safety Follow-up	Follow Up Visits [b]	Survival Follow-Up
Scheduling Window (Days):		± 3	± 3	± 3	± 3	± 3	± 3	± 3	At time of discon	30 days from last dose (± 3 days)	Every 8 weeks post discon (± 7 days)	Every 12 weeks (± 4 weeks)
**Administrative Procedures**												
Eligibility Criteria[c]	X											
Concomitant Medication Review[d]	X	X	X	X	X	X	X	X	X	X		
Trial Treatment Administration[e]	X	X	X	X	X	X	X	X				
Post-study Anticancer Therapy Status											X	X
Survival Status												X
**Clinical Procedures/Assessments**												
Review Adverse Events	X	X	X	X	X	X	X	X	X	X[f]	X[f]	
Full Physical Examination	X				X[g]							
Directed Physical Examination		X	X	X		X[o]	X[o]	X[o]	X			
Vital Signs and Weight	X	X	X	X	X	X	X	X	X			
ECOG Performance Status	X	X	X	X	X	X[p]	X[p]	X[p]	X			
**Laboratory Procedures/Assessments: analysis performed by LOCAL laboratory**												
Pregnancy Test – Urine or Serum β-HCG[h]	X											
PT/INR and aPTT	X[i]											
CBC with Differential[j]	X[i]	X	X	X	X	X	X	X	X	X[n]		
Comprehensive Chemistry Panel[j]	X[i]	X	X	X	X	X	X	X	X	X[n]		
T3, FT4 and TSH[j]	X[i]				X[g]					X[n]		
Tumor Marker Assessment (Optional) [q]	X				X				X		X	
**Efficacy Measurements**												
Tumor Imaging [a,k,l]	X				X				X[m]		X[b]	

a. Treatment cycles are 2 weeks. Imaging should be performed every 8 weeks initially (56 days ± 7 days) and after 6 months, imaging assessments should be performed every 12 weeks regardless of any treatment delays (i.e. Screening visit, Cycle 5, Cycle 9, Cycle 13and then every 6 cycles (12 weeks)).
b. In subjects who discontinue study therapy without documented disease progression, every effort should be made to continue monitoring their disease status by radiologic imaging[k] every 8 weeks (± 7 days) until (1) the start of new anti-cancer treatment, (2) documented disease progression, (3) death, or (4) the end of the study, whichever occurs first.
c. Subjects who either a) attain a CR and discontinue treatment or b) discontinue treatment after 24 months on pembrolizumab (MK-3475) for reasons other than disease progression or intolerability may restart trial treatment if they meet the criteria specified in Section 7.1.5.2.1.
d. Concomitant medications – Enter new medications started during the trial and 30 days after last dose of trial treatment regardless of when the Safety Follow-up visit occurs.
e. Subjects who restart treatment should resume at the same dose and cycle interval which they were receiving prior to discontinuation.
f. Record all AEs occurring within 30 days after the last dose of trial treatment regardless of when the Safety Follow-up visit occurs. After 30 days record all SAEs (related and unrelated to trial treatment) / ECIs occurring up until 90 days after the last dose of trial treatment or the start of new anti-cancer treatment, whichever comes first. Afterwards, any drug related AE regardless of seriousness occurring outside of any reporting timeframes must be reported.
g. To be repeated every 4 cycles after Cycle 5.
h. For women of reproductive potential, a urine pregnancy test will be performed within 72 hours prior to first dose of retreatment. If urine pregnancy results cannot be confirmed as negative, a serum pregnancy test performed by the local study site laboratory will be required. Pregnancy tests (serum and/or urine tests) should be repeated if required by local guidelines.
i. Laboratory tests for determining eligibility for retreatment are to be performed within 10 days prior to the first retreatment dose of pembrolizumab (MK-3475). See Section 7.1.3 for details regarding laboratory tests.
j. After the first dose, lab samples can be collected up to 72 hours prior to the scheduled time point. See Section 7.1.3 for details regarding laboratory tests.
k. A scan must be performed within 28 days prior to restarting treatment with pembrolizumab (MK-3475). Imaging should continue to be performed every 8 weeks (56 ± 7 days) (after 6 months, imaging assessments every 12 weeks) from the first dose of trial treatment or more frequently if clinically indicated. Imaging timing should follow calendar days and should not be adjusted for delays in cycle starts or withholding of pembrolizumab (MK-3475) cycle frequencies. The same imaging technique should be used in a subject throughout the trial. Local reading (investigator assessment with site radiology reading) will be used to determine eligibility and for subject management. Tumor imaging and assessment per local standard of care should be performed for patient management, and may include additional imaging (e.g. bone scan for prostate cancer patients) and appropriate tumor markers. The Sponsor will collect radiological assessments for retrospective analysis by a central vendor. The processes for image collection and transmission to the central vendor are in the Site Imaging Manual (SIM).
l. Per the modified RECIST 1.1 used in this protocol, if imaging shows progressive disease, the imaging assessment should be performed at a minimum of 4 weeks later in order to confirm progressive disease as described in Section 4.2.3.1.
m. If a scan was obtained within 4 weeks prior to the date of discontinuation, then a scan at treatment discontinuation isn't mandatory. In subjects who discontinue study therapy without confirmed disease progression, a radiological evaluation should be performed at the time of treatment discontinuation (i.e. date of discontinuation ± 4 week window). If a previous scan was obtained within 4 weeks prior to the date of discontinuation, then a scan at treatment discontinuation isn't mandatory.
n. Unresolved labs that are drug related AEs should be followed until resolution.
o. Directed physical exams are performed at Cycles 2, 3, 4, 6, 7, 8 and 11. After Cycle 11, directed physical exams should occur once every 4 cycles (Cycles 15, 19, etc.).
p. Following Cycle 8, the ECOG performance status should be determined only in conjunction with a protocol-specified full or directed physical exam (Cycle 9, 11, 13, 15, 17, 19 and every 2 cycles thereafter.
q. Tumor marker assessment is not an additional study-related laboratory evaluation. The purpose is to collect information that may be a part of standard clinical assessment for certain tumor types. Data collection (if applicable and if available) should occur every 8 weeks initially (56 days ± 7 days) and after 6 months, assessments should be performed every 12 weeks regardless of any treatment delays (i.e. Screening visit, Cycle 5, Cycle 9, Cycle 13 and then every 6 cycles (12 weeks) thereafter coinciding with imaging visits). Upon discontinuation, tumor marker data collection will occur every 8 weeks coinciding with post-treatment imaging follow-up visits. See Section 7.1.2.7 and Section 7.1.3.1 for additional details.

7.0 TRIAL PROCEDURES

7.1 Trial Procedures

The Trial Flow Chart - Section 6.0 summarizes the trial procedures to be performed at each visit. Individual trial procedures are described in detail below. It may be necessary to perform these procedures at unscheduled time points if deemed clinically necessary by the investigator.

Furthermore, additional evaluations/testing may be deemed necessary by the investigator and or the Sponsor for reasons related to subject safety. In some cases, such evaluation/testing may be potentially sensitive in nature (e.g., HIV, Hepatitis C, etc.), and thus local regulations may require that additional informed consent be obtained from the subject. In these cases, such evaluations/testing will be performed in accordance with those regulations.

7.1.1 Administrative Procedures

7.1.1.1 Informed Consent

The investigator or qualified designee must obtain documented consent from each potential subject or each subject's legally acceptable representative prior to participating in a clinical trial or Future Biomedical Research.

7.1.1.1.1 General Informed Consent

Consent must be documented by the subject's dated signature or by the subject's legally acceptable representative's dated signature on a consent form along with the dated signature of the person conducting the consent discussion.

A copy of the signed and dated consent form should be given to the subject before participation in the trial.

The initial informed consent form, any subsequent revised written informed consent form and any written information provided to the subject must receive the IRB/ERC's approval/favorable opinion in advance of use. The subject or his/her legally acceptable representative should be informed in a timely manner if new information becomes available that may be relevant to the subject's willingness to continue participation in the trial. The communication of this information will be provided and documented via a revised consent form or addendum to the original consent form that captures the subject's dated signature or by the subject's legally acceptable representative's dated signature.

Specifics about a trial and the trial population will be added to the consent form template at the protocol level.

The informed consent will adhere to IRB/ERC requirements, applicable laws and regulations and Sponsor requirements.

7.1.1.1.2 Consent and Collection of Specimens for Future Biomedical Research

The investigator or qualified designee will explain the Future Biomedical Research consent to the subject, answer all of his/her questions, and obtain written informed consent before performing any procedure related to the Future Biomedical Research sub-trial. A copy of the informed consent will be given to the subject.

7.1.1.2 Inclusion/Exclusion Criteria

All inclusion and exclusion criteria will be reviewed by the investigator or qualified designee to ensure that the subject qualifies for the trial.

7.1.1.3 Subject Identification Card

All subjects will be given a Subject Identification Card identifying them as participants in a research trial. The card will contain trial site contact information (including direct telephone numbers) to be utilized in the event of an emergency. The investigator or qualified designee will provide the subject with a Subject Identification Card immediately after the subject provides written informed consent.

7.1.1.4 Medical History

A medical history will be obtained by the investigator or qualified designee. Medical history will include all active conditions, and any condition diagnosed within the prior 10 years that are considered to be clinically significant by the Investigator. Details regarding the disease for which the subject has enrolled in this study will be recorded separately and not listed as medical history.

7.1.1.5 Prior and Concomitant Medications Review

7.1.1.5.1 Prior Medications

The investigator or qualified designee will review prior medication use, including any protocol-specified washout requirement, and record prior medication taken by the subject within 28 days before starting the trial. Treatment for the disease for which the subject has enrolled in the study will be recorded separately and not listed as a prior medication.

7.1.1.5.2 Concomitant Medications

The investigator or qualified designee will record medication, if any, taken by the subject during the trial. All medications related to reportable SAEs and ECIs should be recorded as defined in Section 7.2.

7.1.1.6 Disease Details and Treatments

7.1.1.6.1 Disease Details

The investigator or qualified designee will obtain prior and current details regarding disease status.

7.1.1.6.2 Prior Treatment Details

The investigator or qualified designee will review all prior cancer treatments including systemic treatments, radiation and surgeries.

7.1.1.6.3 Subsequent Anti-Cancer Therapy Status

The investigator or qualified designee will review all new anti-cancer therapy initiated after the last dose of trial treatment. If a subject initiates a new anti-cancer therapy within 30 days after the last dose of trial treatment, the 30 day Safety Follow-up visit must occur before the first dose of the new therapy. Once new anti-cancer therapy has been initiated the subject will move into survival follow-up.

7.1.1.7 Assignment of Screening Number

All consented subjects will be given a unique screening number that will be used to identify the subject for all procedures that occur prior to randomization or allocation. Each subject will be assigned only one screening number. Screening numbers must not be re-used for different subjects.

Any subject who is screened multiple times will retain the original screening number assigned at the initial screening visit.

7.1.1.8 Assignment of Randomization Number

All eligible subjects will be allocated, by non-random assignment, and will receive a randomization number. The randomization number identifies the subject for all procedures occurring after treatment allocation. This unique number is termed a randomization number throughout the protocol for operational purposes. Once a randomization number is assigned to a subject, it can never be re-assigned to another subject.

A single subject cannot be assigned more than 1 randomization number.

7.1.1.9 Trial Compliance (Medication/Diet/Activity/Other)

Interruptions from the protocol specified treatment plan for greater than 12 weeks between pembrolizumab (MK-3475) doses due to toxicity require consultation between the investigator and the Sponsor and written documentation of the collaborative decision on subject management.

Administration of trial medication will be witnessed by the investigator and/or trial staff. The total volume of trial treatment infused will be compared to the total volume prepared to determine compliance with each dose administered.

The instructions for preparing and administering pembrolizumab (MK-3475) will be provided in the Pharmacy Manual.

7.1.2 Clinical Procedures/Assessments

7.1.2.1 Adverse Event (AE) Monitoring

The investigator or qualified designee will assess each subject to evaluate for potential new or worsening AEs as specified in the Trial Flow Chart and more frequently if clinically indicated. Adverse experiences will be graded and recorded throughout the study and during the follow-up period according to NCI CTCAE Version 4.0 (see Section 12.5). Toxicities will be characterized in terms regarding seriousness, causality, toxicity grading, and action taken with regard to trial treatment.

All AEs of unknown etiology associated with pembrolizumab (MK-3475) exposure should be evaluated to determine if it is possibly an event of clinical interest (ECI) of a potentially immunologic etiology (irAE). See Section 5.6.1.1 and the separate guidance document in the administrative binder regarding the identification, evaluation and management of AEs of a potential immunological etiology.

Please refer to section 7.2 for detailed information regarding the assessment and recording of AEs.

7.1.2.2 Physical Exam

7.1.2.2.1 Full Physical Exam

The investigator or qualified designee will perform a complete physical exam during the screening period. Clinically significant abnormal findings should be recorded as medical history. A full physical exam should be performed during Screening, at Cycle 5 and every 4 cycles thereafter (Cycle 9, 13, 17, etc.). After the first dose of trial treatment new clinically significant abnormal findings should be recorded as AEs.

7.1.2.2.2 Directed Physical Exam

For cycles that do not require a full physical exam per the Trial Flow Chart, the investigator or qualified designee will perform a directed physical exam as clinically indicated prior to trial treatment administration (Cycles 1, 2, 3, 4, 6, 7, 8 and 11). After Cycle 11 directed physical exams should occur once every 4 cycles thereafter (Cycles 15, 19, etc.). New clinically significant abnormal findings should be recorded as AEs.

7.1.2.3 Vital Signs

The investigator or qualified designee will take vital signs at screening, prior to the administration of each dose of trial treatment and at treatment discontinuation as specified in the Trial Flow Chart (Section 6.0). Vital signs should include temperature, pulse, respiratory rate, weight and blood pressure. Height will be measured at screening only.

7.1.2.4 Eastern Cooperative Oncology Group (ECOG) Performance Scale

The investigator or qualified designee will assess ECOG status (see Section 12.4) at screening, prior to the administration of each dose of trial treatment and discontinuation of trial treatment as specified in the Trial Flow Chart. After Cycle 8 assessment of ECOG status will be performed every other cycle in conjunction with the directed or full physical exam.

7.1.2.5 Tumor Imaging and Assessment of Disease

Processes for image collection and transmission to the central vendor can be found in the Site Imaging Manual (SIM).

7.1.2.5.1 Assessment of Disease

RECIST 1.1 will be applied by the site as the primary measure for assessment of tumor response and as a basis for all protocol guidelines related to disease status (e.g., discontinuation of study therapy).

RECIST 1.1 will be adapted as follows to account for the unique tumor response seen in this class of therapeutics.

If imaging shows PD, tumor assessment should be repeated ≥4 weeks later in order to confirm PD with the option of continuing treatment for clinically stable subjects as discussed below in Table 6.

. Clinically stable is defined by the following criteria:

- Absence of signs and symptoms indicating disease progression
- No decline in ECOG performance status
- Absence of rapid progression of disease
- Absence of progressive tumor at critical anatomical sites (e.g., cord compression) requiring urgent alternative medical intervention

Table 6 Imaging and Treatment after 1st Radiologic Evidence of PD

	Clinically Stable		Clinically Unstable	
	Imaging	Treatment	Imaging	Treatment
1st radiologic evidence of PD	Repeat imaging at ≥ 4 weeks to confirm PD	May continue study treatment at the Investigator's discretion while awaiting confirmatory scan	Repeat imaging at ≥ 4 weeks to confirm PD if possible	Discontinue treatment
Repeat scan confirms PD	No additional imaging required	Discontinue treatment (exception noted in Section 7.1.2.5.1)	No additional imaging required	N/A
Repeat scan shows SD, PR or CR	Continue regularly scheduled imaging assessments every 8 weeks (after 6 months every 12 weeks)	Continue study treatment at the Investigator's discretion	Continue regularly scheduled imaging assessments every 8 weeks (after 6 months every 12 weeks)	May restart study treatment if condition has improved and/or clinically stable per Investigator's discretion

In determining whether or not the tumor burden has increased or decreased, Investigators should consider all target lesions as well as non-target lesions (please refer to the Procedures Manual). Subjects that are deemed clinically unstable are not required to have repeat imaging for confirmation. If radiologic progression is confirmed, then the subject will be discontinued from trial treatment. If radiologic progression is not confirmed, then the subject should resume/continue trial treatment and have their next scan according to the every 8 or 12 weeks (56 or 84 ±7 days) schedule based on their on-treatment imaging frequency.

NOTE: If a subject with confirmed radiographic progression (i.e. 2 scans at least 28 days apart demonstrating progressive disease) is clinically stable or clinically improved, and there is no further increase in the tumor dimensions at the confirmatory scan, an exception may be considered to continue treatment upon consultation with the Sponsor. Clinically stable subjects should also have at the confirmatory scan no further increase in the target lesions, no unequivocal increase in non-target lesions, and no additional new lesions develop (non-worsening PD) to continue study treatment.

Imaging during the follow-up period is to be repeated every 8 weeks (56 ±7 days) for subjects who discontinue trial treatment for reasons other than disease progression until the subject experiences confirmed disease progression or starts a new anti-cancer therapy.

Local reading (investigator assessment with site radiology reading) based on RECIST 1.1 will be used to determine subject eligibility and for subject management. Tumor imaging and assessment per local standard of care should be performed for patient management, and may include additional imaging (e.g. bone scan for prostate cancer patients) and appropriate tumor markers. The Sponsor will also receive radiologic images for a retrospective analysis of subject eligibility and treatment response to be performed by a central vendor, which may include RECIST 1.1, RANO, PCWG2 tumor response.

7.1.2.5.2 Initial Tumor Imaging

Initial tumor imaging must be performed within 28 days prior to the first dose of trial treatment. The site study team must review pre-trial images to confirm the subject has measurable disease per RECIST 1.1. The baseline imaging scan should be submitted to the central imaging vendor for a possible retrospective analysis of this eligibility criterion.

Scans performed as part of routine clinical management are acceptable for use as the screening scan if they are of diagnostic quality and performed within 28 days prior to the first dose of trial treatment. The same imaging technique should be used in a subject throughout the study.

7.1.2.5.3 Tumor Imaging During Trial

Tumor imaging may be performed by CT or magnetic resonance imaging (MRI), but the same imaging technique should be used in a subject throughout the trial. Imaging should be performed every 8 weeks (56 days ± 7 days) from the first dose of trial treatment or more frequently if clinically indicated. After 6 months, imaging should be performed every 12 weeks (84 ± 7 days). Imaging should not be delayed for delays in cycle starts or withholding of pembrolizumab (MK-3475) cycle intervals.

Per RECIST 1.1, response should be confirmed by a repeat radiographic assessment not less than 4 weeks from the date the response was first documented. The scan for confirmation of response may be performed at the earliest 4 weeks after the first indication of response, or at the next scheduled scan, whichever is clinically indicated.

Imaging should continue to be performed until documented disease progression, the start of new anti-cancer treatment, withdrawal of consent, death, or the end of the study, whichever occurs first. Disease progression should be confirmed at least 4 weeks after the first scan indicating progressive disease in clinically stable subjects. Subjects who have unconfirmed disease progression may continue on treatment until progression is confirmed provided they have met the conditions detailed in Section 7.1.2.5.1.

7.1.2.6 Tumor Tissue Collection and Correlative Studies Blood Sampling

Enrollment in this study is limited to those subjects with tumors that are characterized as PD-L1 positive by IHC. Archived FFPE tumor sample or newly obtained core or excisional biopsy (FNA not adequate) must be submitted for characterization at a central lab to determine subject eligibility. These samples are not required to be obtained within 28 days

of enrollment, however, a biopsy for screening purposes cannot be performed until the main consent is signed.

Blood for correlative biomarker studies should be collected prior to Cycle 1, at Cycle 5 and upon Discontinuation / End of Treatment.

Detailed instructions for tissue collection, process and shipment are provided in the Procedures Manual.

7.1.2.7 Tumor Marker Assessment (Optional)

Tumor marker assessment is not an additional study-related laboratory evaluation. The purpose is to collect information that may be a part of standard clinical assessment for certain tumor types. Data collection (if applicable and if available) should occur every 8 weeks (± 7 days) initially and after 6 months, assessments should be performed every 12 weeks (± 7 days) regardless of any treatment delays (i.e., Screening visit, Cycle 5, Cycle 9, Cycle 13 and then every 6 cycles [12 weeks] thereafter coinciding with imaging visits). Upon discontinuation, tumor marker data collection will occur every 8 weeks coinciding with post-treatment imaging follow-up visits.

Table 7 shows tumor types that are commonly evaluated using markers.

Table 7 Tumor Markers and Associated Tumor Type

Tumor Type	Tumor Marker
ER Positive HER2 Negative Breast Cancer	Cancer Antigen 15-3 (CA 15-3)
	Cancer Antigen 27-29 (CA 27-29)
Carcinoid Tumors	Chromogranin A (CgA)
	5-Hydroxyindoleacetic acid (5-HIAA) (24 hours in urine)
Colon or Rectal Adenocarcinoma	Carcinoembryonic Antigen (CEA)
Neuroendocrine Carcinoma	Chromogranin A (CgA)
	5-Hydroxyindoleacetic acid (5-HIAA) (24 hours in urine)
Ovarian Epithelial, Fallopian Tube or Primary Peritoneal Carcinoma	Cancer Antigen 125 (CA-125)
Pancreas Adenocarcinoma	Carbohydrate Antigen 19-9 (CA 19-9)
Prostate Adenocarcinoma	Prostate-Specific Antigen (PSA)
Thyroid Cancer	Thyroglobulin (Tg)

7.1.3 Laboratory Procedures/Assessments

Details regarding specific laboratory procedures/assessments to be performed in this trial are provided below. The total amount of blood/tissue to be drawn/collected over the course of the trial (from pre-trial to post-trial visits), including approximate blood/tissue volumes

drawn/collected by visit and by sample type per subject can be found in the Procedures Manual.

7.1.3.1 Laboratory Safety Evaluations (Hematology, Chemistry and Urinalysis)

Laboratory tests for hematology, chemistry and urinalysis are specified in Table 8.

Table 8 Laboratory Tests

Hematology	Chemistry	Urinalysis	Other
Hematocrit	Albumin	Blood	Serum β-human Chorionic Gonadotropin [a] (β-hCG) [a]
Hemoglobin	Alkaline Phosphatase	Glucose	PT (INR)
Platelet Count	Alanine Aminotransferase (ALT)	Protein	aPTT
WBC (Total and Differential)	Aspartate Aminotransferase (AST)	Specific Gravity	Total Triiodothyronine (T3) [e]
Red Blood Cell Count	Lactate Dehydrogenase (LDH)	Microscopic Exam *(If abnormal results are noted)* [c]	Free Thyroxine (FT4)
Absolute Neutrophil Count	Carbon Dioxide [b] *(CO_2 or bicarbonate)*	Urine Pregnancy Test [a]	Thyroid Stimulating Hormone (TSH)
Absolute Lymphocyte Count	Uric Acid		Blood for FBR
	Calcium		Blood for Correlative Studies
	Chloride		Cancer Antigen 15-3 (CA 15-3) [d]
	Glucose		Carbohydrate Antigen 19-9 (CA 19-9) [d]
	Phosphorus		Cancer Antigen 27-29 (CA 27-29) [d]
	Potassium		Cancer Antigen 125 (CA-125) [d]
	Sodium		Carcinoembryonic Antigen (CEA) [d]
	Magnesium		Chromogranin A (CgA) [d]
	Total Bilirubin		Prostate-Specific Antigen (PSA) [d]
	Direct Bilirubin *(If total bilirubin is elevated above the upper limit of normal)*		Thyroglobulin (Tg) [d]
	Total protein		5-Hydroxyindoleacetic acid (5-HIAA) [d] *(24 hours in urine)*
	Blood Urea Nitrogen		
	Creatinine [f]		

a. Perform on women of childbearing potential only. If urine pregnancy results cannot be confirmed as negative, a serum pregnancy test will be required.
b. If considered standard of care in your region.
c. Institutional standards are acceptable.
d. Tumor marker assessment is not an additional study-related laboratory evaluation. The purpose is to collect information that may be a part of standard clinical assessment for certain tumor types.
e. Free T3 may be performed in place of Total T3 per local standards
f. GFR (measured or calculated) or CrCl can be used in place of creatinine.

Laboratory tests for screening or entry into the Second Course Phase should be performed within 10 days prior to the first dose of treatment. After Cycle 1, pre-dose laboratory procedures can be conducted up to 72 hours prior to dosing. Results must be reviewed by the investigator or qualified designee and found to be acceptable prior to each dose of trial treatment.

7.1.3.2 Future Biomedical Research

The following specimens are to be obtained as part of Future Biomedical Research:

- Blood for genomics use
- Leftover archival tumor tissue or leftover newly obtained biopsy sample
- Leftover correlative blood samples

7.1.4 Other Procedures

7.1.4.1 Withdrawal/Discontinuation

When a subject discontinues/withdraws from participation in the trial, all applicable activities scheduled for the final trial visit should be performed at the time of discontinuation. Any adverse events which are present at the time of discontinuation/withdrawal should be followed in accordance with the safety requirements outlined in Section 7.2 - Assessing and Recording Adverse Events. Subjects who a) attain a CR or b) complete 24 months of treatment with pembrolizumab (MK-3475) may discontinue treatment with the option of restarting treatment if they meet the criteria specified in Section 7.1.5.2.1. After discontinuing treatment following assessment of CR or 24 months of therapy, these subjects should return to the site for a Safety Follow-up Visit (described in Section 7.1.5.3.1) and then proceed to the Follow-Up Period of the study (described in Section 7.1.5.4).

7.1.4.1.1 Withdrawal From Future Biomedical Research

Subjects may withdraw their consent for Future Biomedical Research and have their specimens and all derivatives destroyed. Subjects may withdraw consent at any time by contacting the principal investigator for the main trial. If medical records for the main trial are still available, the investigator will contact the Sponsor using the designated mailbox (clinical.specimen.management@merck.com), and a form will be provided by the Sponsor to obtain appropriate information to complete specimen withdrawal. Subsequently, the subject's specimens will be removed from the biorepository and be destroyed. A letter will be sent from the Sponsor to the investigator confirming the destruction. It is the responsibility of the investigator to inform the subject of completion of destruction. Any analyses in progress at the time of request for destruction or already performed prior to the request being received by the Sponsor will continue to be used as part of the overall research trial data and results. No new analyses would be generated after the request is received.

In the event that the medical records for the main trial are no longer available (e.g., if the investigator is no longer required by regulatory authorities to retain the main trial records) or the specimens have been completely anonymized, there will no longer be a link between the subject's personal information and their specimens. In this situation, the request for specimen destruction cannot be processed.

7.1.4.2 Blinding/Unblinding

This is an open label trial; there is no blinding for this trial.

7.1.4.3 Calibration of Critical Equipment

The investigator or qualified designee has the responsibility to ensure that any critical device or instrument used for a clinical evaluation/test during a clinical trial that provides important information about inclusion/exclusion criteria and/or safety or efficacy parameters shall be suitably calibrated and maintained to ensure that the data obtained is reliable and/or reproducible. Documentation of equipment calibration must be retained with the study documentation as source documentation at the trial site.

Critical Equipment for this trial includes:

- Laboratory equipment – as required for inclusion labs and trial assessments
- Imaging equipment – as required for study objectives

See protocol-specific Administrative Binder, Procedures Manual, Pharmacy Manual, and Site Imaging Manual.

7.1.5 Visit Requirements

Visit requirements are outlined in Section 6.0 - Trial Flow Chart. Specific procedure-related details are provided above in Section 7.1 - Trial Procedures.

7.1.5.1 Screening Period

Approximately 28 days prior to enrollment, potential subjects will be evaluated to determine that they fulfill the entry requirements as set forth in Section 5.1. Visit requirements are outlined in Section 6.0 – Trial Flow Chart.

Subjects that have an archival tumor biopsy sample may sign a prescreening consent for characterization of PD-L1 status. After providing prescreening consent, subjects will be assigned a screening number. Subjects characterized with a PD-L1 positive tumor must subsequently provide written consent for the main study prior to performing any protocol specific procedure. As noted in Section 7.2, AEs will NOT be collected for subjects during this pre-screening period (for determination of archival tissue status) as long as that subject has not undergone any protocol-specified procedure or intervention beyond archive sample collection for PD-L1 characterization.

Subjects that do not have an archival tumor biopsy sample available must provide written consent for the main study before the newly obtained tumor biopsy or any other protocol-specified procedures can occur. After providing main study consent, subjects not already provided a screening number will be assigned a screening number.

Results of a test performed prior to the subject signing consent as part of routine clinical management are acceptable in lieu of a screening test if performed within the specified time frame. Screening procedures are to be completed within 28 days prior to the first dose of trial treatment except for the following:

- Laboratory tests are to be performed within 10 days prior to the first dose of trial treatment
- For women of reproductive potential, a urine pregnancy test will be performed within 72 hours prior to the first dose of trial treatment. If urine pregnancy results cannot be confirmed as negative, a serum pregnancy test will be required (performed by the local study site laboratory)
- Tumor collection and PD-L1 characterization are not required to be completed within 28 days prior to the first dose of trial treatment

Subjects may be rescreened after initially failing to meet the inclusion/exclusion criteria. Results from assessments performed during the initial screening period are acceptable in lieu of a repeat screening test if performed within the specified time frame and the inclusion/exclusion criteria is met.

7.1.5.2 Treatment Period

7.1.5.2.1 Second Course Phase (Retreatment Period)

Subjects who stop pembrolizumab (MK-3475) with SD or better may be eligible for up to one year of additional pembrolizumab (MK-3475) therapy if they progress after stopping MK-3745. This retreatment is termed the Second Course Phase of this study and is only available if the study remains open and the subject meets the following conditions:

- Either
    - Stopped initial treatment with pembrolizumab (MK-3475) after attaining an investigator-determined confirmed CR according to RECIST 1.1
        - Was treated for at least 24 weeks with pembrolizumab (MK-3475) before discontinuing therapy
        - Received at least two treatments with pembrolizumab (MK-3475) beyond the date when the initial CR was declared

    OR

    - Subject had SD, PR or CR and stopped pembrolizumab (MK-3475) treatment after 24 months of study therapy for reasons other than disease progression or intolerability

AND

- Experienced an investigator-determined confirmed radiographic disease progression after stopping their initial treatment with pembrolizumab (MK-3475)

- Did not receive any anti-cancer treatment since the last dose of pembrolizumab (MK-3475)

- Have a performance status of 0 or 1 on the ECOG Performance Scale

- Demonstrate adequate organ function as detailed in Section 5.1.2

- Female subject of childbearing potential should have a negative urine or serum pregnancy test within 72 hours prior to receiving retreatment with study medication.

- Female subjects of childbearing potential should be willing to use 2 methods of birth control or be surgically sterile, or abstain from heterosexual activity for the course of the study through 120 days after the last dose of study medication (Reference Section 5.7.2). Subjects of child bearing potential are those who have not been surgically sterilized or have been free from menses for >1 year.

- Male subjects should agree to use an adequate method of contraception starting with the first dose of study therapy through 120 days after the last dose of study therapy.

- Does not have a history or current evidence of any condition, therapy, or laboratory abnormality that might interfere with the subject's participation for the full duration of the trial or is not in the best interest of the subject to participate, in the opinion of the treating investigator.

Subjects who restart treatment will be retreated at the same dose frequency as when they last received pembrolizumab (MK-3475). Treatment will be administered for up to one additional year.

Visit requirements are outlined in Section 6.0 – Trial Flow Chart.

7.1.5.3 Post-Treatment Visits

7.1.5.3.1 Safety Follow-Up Visit

The mandatory Safety Follow-Up Visit should be conducted approximately 30 days (±3 days) after the last dose of trial treatment or before the initiation of a new anti-cancer treatment, whichever comes first.

All AEs and concomitant medications within 30 days of last dose of trial treatment, regardless of when the Safety Follow-Up visit occurs should be recorded. After day 30, all SAEs and ECIs continue to be captured until 90 days after last dose of trial treatment or before initiation of a new anti-cancer treatment, whichever comes first. Subjects with an AE

of Grade >1 will be followed until the resolution of the AE to Grade 0-1 or until the beginning of a new anti-cancer therapy, whichever occurs first.

Subjects who are eligible for retreatment with pembrolizumab (MK-3475) (as described in Section 7.1.5.2.1) may have up to two safety follow-up visits, one after the Treatment Period and one after the Second Course Phase.

7.1.5.4 Follow-up Visits

Subjects who discontinue trial treatment for a reason other than disease progression will move into the Follow-Up Phase and should be assessed every 8 weeks (56 ±7 days) by radiologic imaging to monitor disease status. Every effort should be made to collect information regarding disease status until the start of new anti-cancer therapy, disease progression, death, end of the study or if the subject begins retreatment with pembrolizumab (MK-3475) as detailed in Section 7.1.5.2.1. Information regarding post-study anti-cancer treatment will be collected if new treatment is initiated.

Subjects who are eligible to receive retreatment with pembrolizumab (MK-3475) according to the criteria in Section 7.1.5.2.1 will move from the follow-up phase to the Second Course Phase when they experience disease progression. Details are provided in Section 6.2 – Trial Flow Chart for Retreatment.

7.1.5.4.1 Survival Follow-up

Once a subject experiences confirmed disease progression or starts a new anti-cancer therapy, the subject moves into the survival follow-up phase and should be contacted by telephone every 12 weeks (± 4 weeks) to assess for survival status until death, withdrawal of consent, or the end of the study, whichever occurs first.

7.2 Assessing and Recording Adverse Events

An adverse event is defined as any untoward medical occurrence in a patient or clinical investigation subject administered a pharmaceutical product and which does not necessarily have to have a causal relationship with this treatment. An adverse event can therefore be any unfavourable and unintended sign (including an abnormal laboratory finding, for example), symptom, or disease temporally associated with the use of a medicinal product or protocol-specified procedure, whether or not considered related to the medicinal product or protocol-specified procedure. Any worsening (i.e., any clinically significant adverse change in frequency and/or intensity) of a preexisting condition that is temporally associated with the use of the Sponsor's product, is also an adverse event.

Changes resulting from normal growth and development that do not vary significantly in frequency or severity from expected levels are not to be considered adverse events. Examples of this may include, but are not limited to, teething, typical crying in infants and children and onset of menses or menopause occurring at a physiologically appropriate time.

Sponsor's product includes any pharmaceutical product, biological product, device, diagnostic agent or protocol-specified procedure, whether investigational (including placebo or active comparator medication) or marketed, manufactured by, licensed by, provided by or distributed by the Sponsor for human use.

Adverse events may occur during the course of the use of the Sponsor's product in clinical trials or within the follow-up period specified by the protocol, or prescribed in clinical practice, from overdose (whether accidental or intentional), from abuse and from withdrawal.

Adverse events may also occur in screened subjects during any pre-allocation baseline period as a result of a protocol-specified intervention, including washout or discontinuation of usual therapy, diet, placebo treatment or a procedure.

Progression of the cancer under study is not considered an adverse event unless it is considered to be drug related by the investigator.

All adverse events will be recorded from the time the consent form is signed through 30 days following cessation of treatment and at each examination on the Adverse Event case report forms/worksheets. The reporting timeframe for adverse events meeting any serious criteria is described in section 7.2.3.1.

Adverse events will not be collected for subjects during the pre-screening period (for determination of archival tissue status) as long as that subject has not undergone any protocol-specified procedure or intervention. If the subject requires a blood draw, fresh tumor biopsy etc., the subject is first required to provide consent to the main study and AEs will be captured according to guidelines for standard AE reporting.

7.2.1 Definition of an Overdose for This Protocol and Reporting of Overdose to the Sponsor

For purposes of this trial, an overdose will be defined as any dose exceeding the prescribed dose for pembrolizumab (MK-3475) by 20% over the prescribed dose. No specific information is available on the treatment of overdose of pembrolizumab (MK-3475). In the event of overdose, pembrolizumab (MK-3475) should be discontinued and the subject should be observed closely for signs of toxicity. Appropriate supportive treatment should be provided if clinically indicated.

If an adverse event(s) is associated with ("results from") the overdose of Sponsor's product or vaccine, the adverse event(s) is reported as a serious adverse event, even if no other seriousness criteria are met.

If a dose of Sponsor's product or vaccine meeting the protocol definition of overdose is taken without any associated clinical symptoms or abnormal laboratory results, the overdose is reported as a non-serious Event of Clinical Interest (ECI), using the terminology "accidental or intentional overdose without adverse effect."

All reports of overdose with and without an adverse event must be reported within 24 hours to the Sponsor either by electronic media or paper. Sponsor Contact information can be found in the Investigator Trial File Binder (or equivalent).

7.2.2 Reporting of Pregnancy and Lactation to the Sponsor

Although pregnancy and lactation are not considered adverse events, it is the responsibility of investigators or their designees to report any pregnancy or lactation in a subject (spontaneously reported to them) that occurs during the trial or within 120 days of completing the trial, or 30 days following cessation of treatment if the subject initiates new anticancer therapy, whichever is earlier. All subjects who become pregnant must be followed to the completion/termination of the pregnancy. Pregnancy outcomes of spontaneous abortion, missed abortion, benign hydatidiform mole, blighted ovum, fetal death, intrauterine death, miscarriage and stillbirth must be reported as serious events (Important Medical Events). If the pregnancy continues to term, the outcome (health of infant) must also be reported.

Such events must be reported within 24 hours to the Sponsor either by electronic media or paper. Sponsor Contact information can be found in the Investigator Trial File Binder (or equivalent).

7.2.3 Immediate Reporting of Adverse Events to the Sponsor

7.2.3.1 Serious Adverse Events

A serious adverse event is any adverse event occurring at any dose or during any use of Sponsor's product that:

- Results in death;
- Is life threatening;
- Results in persistent or significant disability/incapacity;
- Results in or prolongs an existing inpatient hospitalization;
- Is a congenital anomaly/birth defect;
- Is a new cancer (that is not a condition of the study);
- Is associated with an overdose;
- Is an other important medical event

Refer to Table 9 for additional details regarding each of the above criteria.

Any serious adverse event, or follow up to a serious adverse event, including death due to any cause other than progression of the cancer under study (reference Section 7.2.3.3 for additional details) that occurs to any subject from the time the consent is signed through 90 days following cessation of treatment, or 30 days following cessation of treatment if the subject initiates new anticancer therapy, whichever is earlier, whether or not related to the Sponsor's product, must be reported within 24 hours to the Sponsor either by electronic media or paper. Sponsor Contact information can be found in the Investigator Trial File Binder (or equivalent).

Additionally, any serious adverse event, considered by an investigator who is a qualified physician to be related to the Sponsor's product that is brought to the attention of the investigator at any time outside of the time period specified in the previous paragraph also must be reported immediately to the Sponsor.

All subjects with serious adverse events must be followed up for outcome.

7.2.3.2 Events of Clinical Interest

Selected non-serious and serious adverse events are also known as Events of Clinical Interest (ECI) and must be recorded as such on the Adverse Event case report forms/worksheets and reported within 24 hours to the Sponsor either by electronic media or paper. Sponsor Contact information can be found in the Investigator Trial File Binder (or equivalent).

Events of clinical interest for this trial include:

1. an overdose of Sponsor's product, as defined in Section 7.2.1 - Definition of an Overdose for This Protocol and Reporting of Overdose to the Sponsor, that is not associated with clinical symptoms or abnormal laboratory results.

2. an elevated AST or ALT lab value that is greater than or equal to 3X the upper limit of normal and an elevated total bilirubin lab value that is greater than or equal to 2X the upper limit of normal and, at the same time, an alkaline phosphatase lab value that is less than 2X the upper limit of normal, as determined by way of protocol-specified laboratory testing or unscheduled laboratory testing.*

   <u>*Note:</u> These criteria are based upon available regulatory guidance documents. The purpose of the criteria is to specify a threshold of abnormal hepatic tests that may require an additional evaluation for an underlying etiology. The trial site guidance for assessment and follow up of these criteria can be found in the Investigator Trial File Binder (or equivalent).

3. Additional adverse events:

   A separate guidance document has been provided entitled “Event of Clinical Interest Guidance Document” (previously entitled, “Event of Clinical Interest and Immune-Related Adverse Event Guidance Document”). This document can be found in the administrative binder and provides guidance regarding identification, evaluation and management of ECIs and irAEs.

   ECIs (both non-serious and serious adverse events) identified in this guidance document from the date of first dose through 90 days following cessation of treatment, or 30 days after the initiation of a new anticancer therapy, whichever is earlier, need to be reported to the SPONSOR **within 24 hours** of the event, regardless of attribution to study treatment, consistent with standard SAE reporting guidelines and either by electronic media or paper. Sponsor Contact information can be found in the administrative binder.

Subjects should be assessed for possible ECIs prior to each dose. Lab results should be evaluated and subjects should be asked for signs and symptoms suggestive of an immune-related event. Subjects who develop an ECI thought to be immune-related should have additional testing to rule out other etiologic causes. If lab results or symptoms indicate a possible immune-related ECI, then additional testing should be performed to rule out other etiologic causes. If no other cause is found, then it is assumed to be immune-related.

7.2.3.3 Protocol-Specific Exceptions to Serious Adverse Event Reporting

Efficacy endpoints as outlined in this section will not be reported to the Sponsor as described in Section 7.2.3 - Immediate Reporting of Adverse Events to the Sponsor, unless there is evidence suggesting a causal relationship between the drug and the event. Any such event will be submitted to the Sponsor within 24 hours either by electronic or paper media.

Specifically, the suspected/actual events covered in this exception include any event that is disease progression of the cancer under study.

The Sponsor will monitor unblinded aggregated efficacy endpoint events and safety data to ensure the safety of the subjects in the trial. Any suspected endpoint which upon review is not progression of the cancer under study will be forwarded to global safety as a SAE within 24 hours of determination that the event is not progression of the cancer under study.

7.2.4 Evaluating Adverse Events

An investigator who is a qualified physician will evaluate all adverse events according to the NCI Common Terminology for Adverse Events (CTCAE), version 4.0. Any adverse event which changes CTCAE grade over the course of a given episode will have each change of grade recorded on the adverse event case report forms/worksheets.

All adverse events regardless of CTCAE grade must also be evaluated for seriousness.

Table 9 Evaluating Adverse Events
An investigator who is a qualified physician, will evaluate all adverse events as to:

**V4.0 CTCAE Grading**	**Grade 1**	**Mild; asymptomatic or mid symptoms; clinical or diagnostic observations only; intervention not indicated.**
	**Grade 2**	**Moderate; minimal, local or noninvasive intervention indicated; limiting age-appropriate instrumental ADL.**
	**Grade 3**	**Severe or medically significant but not immediately life-threatening; hospitalization or prolongation or hospitalization indicated; disabling; limiting self-care ADL.**
	**Grade 4**	**Life threatening consequences; urgent intervention indicated.**
	**Grade 5**	**Death related to AE**
**Seriousness**	A serious adverse event is any adverse event occurring at any dose or during any use of Sponsor's product that:	
	†**Results in death**; or	
	†**Is life threatening; or** places the subject, in the view of the investigator, at immediate risk of death from the event as it occurred (Note: This does not include an adverse event that, had it occurred in a more severe form, might have caused death.); or	
	†**Results in a persistent or significant disability/incapacity** (substantial disruption of one's ability to conduct normal life functions); or	
	†**Results in or prolongs an existing inpatient hospitalization** (hospitalization is defined as an inpatient admission, regardless of length of stay, even if the hospitalization is a precautionary measure for continued observation. (Note: Hospitalization for an elective procedure to treat a pre-existing condition that has not worsened is not a serious adverse event. A pre-existing condition is a clinical condition that is diagnosed prior to the use of a Merck product and is documented in the patient's medical history.); or	
	†**Is a congenital anomaly/birth defect** (in offspring of subject taking the product regardless of time to diagnosis);or	
	**Is a** new **cancer;** (that is not a condition of the study) **or**	
	**Is an overdose** (whether accidental or intentional). Any adverse event associated with an overdose is considered a serious adverse event. An overdose that is not associated with an adverse event is considered a non-serious event of clinical interest and must be reported within 24 hours.	
	**Other important medical events** that may not result in death, not be life threatening, or not require hospitalization may be considered a serious adverse event when, based upon appropriate medical judgment, the event may jeopardize the subject and may require medical or surgical intervention to prevent one of the outcomes listed previously (designated above by a †).	
**Duration**	Record the start and stop dates of the adverse event. If less than 1 day, indicate the appropriate length of time and units	
**Action taken**	Did the adverse event cause the Sponsor's product to be discontinued?	
**Relationship to test drug**	Did the Sponsor's product cause the adverse event? The determination of the likelihood that the Sponsor's product caused the adverse event will be provided by an investigator who is a qualified physician. The investigator's signed/dated initials on the source document or worksheet that supports the causality noted on the AE form, ensures that a medically qualified assessment of causality was done. This initialed document must be retained for the required regulatory time frame. The criteria below are intended as reference guidelines to assist the investigator in assessing the likelihood of a relationship between the test drug and the adverse event based upon the available information.<br>**The following components are to be used to assess the relationship between the Sponsor's product and the AE**; the greater the correlation with the components and their respective elements (in number and/or intensity), the more likely the Sponsor's product caused the adverse event (AE):	
	**Exposure**	Is there evidence that the subject was actually exposed to the Sponsor's product such as: reliable history, acceptable compliance assessment (pill count, diary, etc.), expected pharmacologic effect, or measurement of drug/metabolite in bodily specimen?
	**Time Course**	Did the AE follow in a reasonable temporal sequence from administration of the Sponsor's product?<br>Is the time of onset of the AE compatible with a drug-induced effect (applies to trials with investigational medicinal product)?
	**Likely Cause**	Is the AE not reasonably explained by another etiology such as underlying disease, other drug(s)/vaccine(s), or other host or environmental factors

**Relationship to Sponsor's Product (continued)**	**The following components are to be used to assess the relationship between the test drug and the AE: (continued)**	
	**Dechallenge**	Was the Sponsor’s product discontinued or dose/exposure/frequency reduced?<br>If yes, did the AE resolve or improve?<br>If yes, this is a positive dechallenge. If no, this is a negative dechallenge.<br>(Note: This criterion is not applicable if: (1) the AE resulted in death or permanent disability; (2) the AE resolved/improved despite continuation of the Sponsor’s product; or (3) the trial is a single-dose drug trial); or (4) Sponsor’s product(s) is/are only used one time.)
	**Rechallenge**	Was the subject re-exposed to the Sponsor’s product in this study?<br>If yes, did the AE recur or worsen?<br>If yes, this is a positive rechallenge. If no, this is a negative rechallenge.<br>(Note: This criterion is not applicable if: (1) the initial AE resulted in death or permanent disability, or (2) the trial is a single-dose drug trial); or (3) Sponsor’s product(s) is/are used only one time).<br>NOTE: IF A RECHALLENGE IS PLANNED FOR AN ADVERSE EVENT WHICH WAS SERIOUS AND WHICH MAY HAVE BEEN CAUSED BY THE SPONSOR'S PRODUCT, OR IF REEXPOSURE TO THE SPONSOR'S PRODUCT POSES ADDITIONAL POTENTIAL SIGNIFICANT RISK TO THE SUBJECT, THEN THE RECHALLENGE MUST BE APPROVED IN ADVANCE BY THE SPONSOR CLINICAL DIRECTOR AS PER DOSE MODIFICATION GUIDELINES IN THE PROTOCOL.
	**Consistency with Trial Treatment Profile**	Is the clinical/pathological presentation of the AE consistent with previous knowledge regarding the Sponsor’s product or drug class pharmacology or toxicology?
The assessment of relationship will be reported on the case report forms /worksheets by an investigator who is a qualified physician according to his/her best clinical judgment, including consideration of the above elements.		
**Record one of the following**		**Use the following scale of criteria as guidance (not all criteria must be present to be indicative of a Sponsor’s product relationship).**
**Yes, there is a reasonable possibility of Sponsor's product relationship.**		There is evidence of exposure to the Sponsor's product. The temporal sequence of the AE onset relative to the administration of the Sponsor's product is reasonable. The AE is more likely explained by the Sponsor's product than by another cause.
**No, there is not a reasonable possibility of Sponsor's product relationship**		Subject did not receive the Sponsor's product OR temporal sequence of the AE onset relative to administration of the Sponsor's product is not reasonable OR there is another obvious cause of the AE. (Also entered for a subject with overdose without an associated AE.)

7.2.5 Sponsor Responsibility for Reporting Adverse Events

All Adverse Events will be reported to regulatory authorities, IRB/IECs and investigators in accordance with all applicable global laws and regulations.

8.0 STATISTICAL ANALYSIS PLAN

This section outlines the statistical analysis strategy and procedures for the study. If, after the study has begun, changes are made to primary and/or key secondary hypotheses, or the statistical methods related to those hypotheses, then the protocol will be amended (consistent with ICH Guideline E-9). Changes to exploratory or other non-confirmatory analyses made after the protocol has been finalized, along with an explanation as to when and why they occurred, will be listed in the Clinical Study Report (CSR) for the study. Post hoc exploratory analyses will be clearly identified in the CSR. No separate Statistical Analysis Plan (SAP) for the primary, secondary and exploratory endpoints will be issued for this study, however, a separate molecular profiling analysis plan will be developed to explicitly address exploratory biomarker objectives.

8.1 Statistical Analysis Plan Summary

This section contains a brief summary of the statistical analyses for this trial. Full detail is in the Statistical Analysis Plan (SAP) (Section 8.2).

8.1.1 Efficacy Analyses

The primary and key secondary endpoints, primary analysis population, and statistical methods that will be employed for the efficacy analyses are presented in Table 10 below**.**

The primary hypothesis will be evaluated separately in each disease indication by evaluating best overall response rate by RECIST 1.1. A sequential monitoring approach will be used following the time that a minimum of 6 subjects have had at least one post-baseline scan in each indication.

The Type-I error rate over the multiple evaluations within an indication will be controlled by the truncated sequential probability ratio test procedure at 0.08 (1-sided) [32].

Table 10 Summary of Analysis Strategy for Key Efficacy Endpoints

Endpoint/Variable (Description, Timepoint)	Statistical Method	Analysis Population	Missing Data Approach
Primary Hypothesis #1:			
Best Overall Response Rate by RECIST 1.1 in each disease indication	Truncated sequential probability ratio test [32]	Full Analysis set	Missing observation counted as non-responder
Secondary Objectives – Within Indication			
PFS	Summary statistics using Kaplan-Meier method	FAS	Censored at last assessment
OS	Kaplan-Meier method	FAS	Censored at last assessment
Duration of Response (DOR)	Summary statistics using Kaplan-Meier method	All responders	Non-responders are excluded in analysis

8.1.2 Safety Analyses

The All-Patients-as-Treated population will be employed for safety analyses. Immune related adverse experiences (as defined the Event of Clinical Interest Guidance Document located in the Administrative Binder) are prespecified as events of interest.

8.1.3 Power and Sample Size

Within each indication, the study will enroll a minimum of 6 subjects. Following the time that at least 6 subjects have had at least one post-baseline response assessment, a sequential monitoring procedure will be used to evaluate for efficacy and futility simultaneously based on the number of subjects with a confirmed or unconfirmed response according to the rules outlined in Figure 2, Table 11, and Table 12.

Depending on the enrollment rate, it is possible that more than 6 subjects may be enrolled prior to the first evaluation of efficacy or futility. Enrollment is expected to be continuous and will not be held within an indication.

Once at least 6 subjects are evaluable for confirmed or unconfirmed response, subsequent rules for pausing enrollment and future evaluations will be based on the boundaries identified by the sequential monitoring procedure. A maximum of approximately 22 subjects will be enrolled in each indication. The maximum total sample size is approximately 440 subjects, however, it is expected that the total sample size will be approximately 320 subjects. Further details are provided in section 8.2.7.

With 22 subjects per indication, this study provides 80% power to demonstrate that the best overall response rate induced by pembrolizumab (MK-3475) exceeds 10% at an overall one-sided 8% alpha-level, if the true best overall response rate within an indication is 35%. The underlying treatment effect is regarded as clinically important in each of the indications studied. The calculation is based on the binomialSPRT function in the gsDesign package and

is carried out using R. The minimum criterion for success is that the lower bound of the repeated CI > 10% [33]. Given the underlying true rate, this may occur when at least 6/22 subjects develop a response. Further details are provided in section 8.2.7.

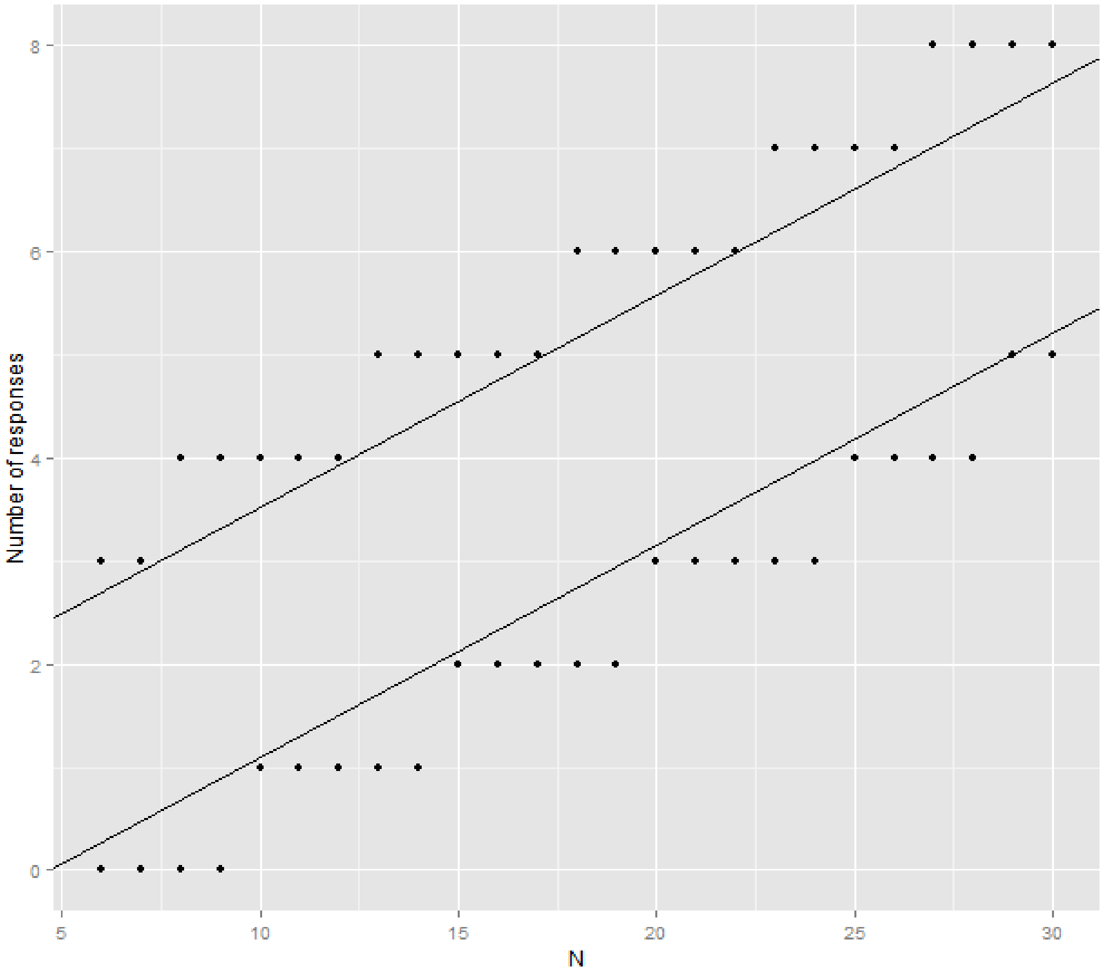

Figure 2 Sequential Monitoring Rules for Efficacy and Futility

Table 11 Decision Rules Based on Efficacy Bounds

Monitoring Point (# Subjects)	Minimum # Responders to Start Future Study Planning
6-7	3
8-12	4
13-17	5
18-22*	6
23-26	7
27-30	8

Design assumes overall Type I error of 8% (1-sided) and 80% power.
*Success at the final analysis requires ≥6/22 subjects with a response.

Table 12 Decision Rules Based on Futility Bounds

Monitoring Point (# Subjects)	Maximum # Subjects with Response to Declare Futility
6-9	0
10-14	1
15-19	2
20-24*	3
25-28	4
29-30	5

Design assumes overall Type I error of 8% (1-sided) and 80% power.
*Success at the final analysis requires ≥6/22 subjects with a response.

8.1.4 Interim Analysis

Multiple interim analyses may be performed in this study due to the sequential design of the trial. Results will be reviewed by the study team. The endpoint(s), timing, and purpose of the interim analysis are summarized in the table below. The decision rule and other statistical details are further described in Section 8.2.9.

Table 13 Summary of Interim Analysis Strategy

Key Endpoints for Interim Analysis	Timing of Interim Analysis	Purpose of Interim Analysis
Objective response rate (confirmed or unconfirmed)	Sequential monitoring approach	• Stop for futility<br>• Go to future study planning

8.2 Statistical Analysis Plan

8.2.1 Responsibility for Analyses/In-House Blinding

The statistical analysis of the data obtained from this study will be the responsibility of the Clinical Biostatistics department of the SPONSOR.

This trial is being conducted as an open-label study, i.e., subjects, investigators, and SPONSOR personnel will be aware of subject treatment assignments after each subject is enrolled and treatment is assigned.

The Clinical Biostatistics department will generate the allocation schedule(s) for study treatment assignment. Allocation will be implemented in an interactive voice response system (IVRS).

8.2.2 Hypotheses/Estimation

Objectives and hypotheses of the study are stated in Section 3.0.

8.2.3 Analysis Endpoints

Efficacy and safety endpoints that will be evaluated for within- and/or between-treatment differences are listed below, followed by the descriptions of the derivations of selected endpoints.

8.2.3.1 Efficacy Endpoints

Efficacy endpoints that will be evaluated for are listed below, followed by the descriptions of the derivations of selected endpoints.

The primary efficacy endpoint is best overall response rate, defined as the proportion of subjects in the analysis population who have complete response (CR) or partial response (PR) using RECIST 1.1 at any time during the study. Response for the primary analysis will be determined by the investigator assessment, and a confirmation assessment is required per RECIST 1.1.

Secondary efficacy endpoints include: (1) duration of response, defined as time from first RECIST 1.1 response to disease progression in subjects who achieve a PR or better; (2) progression-free survival (PFS), defined as the time from allocation to the first documented

disease progression according to RECIST 1.1 or death due to any cause, whichever occurs first; and (3) overall survival (OS).

Additional supportive analyses of best overall response rate, duration of response, and PFS will be conducted using modified RECIST 1.1 criteria, in which a confirmation assessment of disease progression must be obtained at least 4 weeks after the initial disease assessment indicating progressive disease.

8.2.3.2 Safety Endpoints

A description of safety measures is provided in Section 4.2.3.2.

The primary safety endpoints are AEs graded using CTCAE (Version 4.0) criteria. Safety will be assessed by quantifying the toxicities and grades experienced by subjects who have received pembrolizumab (MK-3475), including serious adverse events (SAEs) and events of clinical interest (ECIs). Immune related adverse experiences (irAEs) (as defined the Pembrolizumab Event of Clinical Interest Guidance Document located in the Administrative Binder) are prespecified as events of interest. Other safety endpoints include laboratory safety assessments, ECOG performance status, vital signs and physical examinations.

8.2.4 Analysis Populations

8.2.4.1 Efficacy Analysis Populations

The Full Analysis Set (FAS) population will serve as the primary population for the analysis of efficacy data in this study. The FAS population consists of all subjects within each indication who:

- Receive at least one dose of study treatment, and
- Have a baseline scan with measurable disease per RECIST 1.1

Subjects will be included in the treatment group to which they are allocated for the analysis of efficacy data. Details on the approach to handling missing data are provided in Section 8.2.5 Statistical Methods.

8.2.4.2 Safety Analysis Populations

The All Patients as Treated (APaT) population will be used for the analysis of safety data in this study. The APaT population consists of all allocated subjects who received at least one dose of study treatment.

At least one laboratory or vital sign measurement obtained subsequent to at least one dose of study treatment is required for inclusion in the analysis of each specific parameter. To assess change from baseline, a baseline measurement is also required.

Details on the approach to handling missing data for safety analyses are provided in Section 8.2.5 Statistical Methods.

8.2.5 Statistical Methods

Statistical testing and inference for safety analyses are described in 8.2.5.2. Efficacy results that will be considered to be statistically significant after consideration of the strategy for controlling the Type I error are described in Section 8.2.6, Multiplicity. Nominal p-values may be computed for other efficacy analyses as a measure of strength of association between the endpoint and the treatment effect rather than formal tests of hypotheses. Unless otherwise stated, all statistical tests will be conducted at the α=0.05 (2-sided) level.

8.2.5.1 Statistical Methods for Efficacy Analyses

Efficacy will be evaluated separately in each cohort. For the primary efficacy endpoint investigator assessed RECIST 1.1 best overall response rate, the point estimate, repeated confidence interval, and adjusted p-value for testing the RECIST 1.1 response rate is greater than 10% for each disease indication will be provided using a truncated sequential probability ratio test [32], which is a specific instance of an exact binomial group sequential design for a single arm trial with a binary outcome. Subjects in the primary analysis population (FAS) without response data will be counted as non-responder. Interim decisions may be made based on confirmed or unconfirmed response assessments (see Sections 8.2.7 and 8.2.9). However, the final analysis (if enrollment in a given indication expands to 22 subjects) will require a confirmation assessment for all subjects who develop a CR or PR.

For PFS endpoint, Kaplan-Meier (KM) curves and median estimates from the KM curves will be provided as appropriate. Subjects without efficacy evaluation data or without survival data will be censored at Day 1.

Table 14 summarizes the key efficacy analyses.

Table 14 Analysis Strategy for Key Efficacy Variables

Endpoint/Variable (Description, Time Point)	Primary vs. Supportive Approach†	Statistical Method	Analysis Population	Missing Data Approach
**Primary Hypothesis #1 – Within Indication**				
Best Overall RECIST 1.1 response rate (each disease indication evaluated separately) by site radiology assessment	P	Truncated sequential probability test	FAS	Subjects with missing data are considered non-responders
Best Overall modified RECIST 1.1 response rate (each disease indication evaluated separately) by site radiology assessment	S	Truncated sequential probability test	FAS	Subjects with missing data are considered non-responders
**Secondary Endpoints/Objectives– Within Indication**				
PFS using RECIST 1.1 criteria by site assessment	P	Summary statistics using Kaplan-Meier method	FAS	Censored at last assessment
PFS using modified RECIST 1.1 criteria by site assessment	S	Summary statistics using Kaplan-Meier method	FAS	Censored at last assessment
OS	P	Kaplan-Meier method	FAS	Censored at last assessment
Duration of response (DOR) by site assessment	P	Summary statistics using Kaplan-Meier method	All responders	Non-responders are excluded in analysis
Duration of modified RECIST 1.1 response (DOR) by site assessment	S	Summary statistics using Kaplan-Meier method	All modified RECIST 1.1 responders	Non-responders are excluded in analysis

† P=Primary approach; S=Secondary approach.

The strategy to address multiplicity issues with regard to multiple efficacy endpoints is described in Section 8.2.6, Multiplicity and Section 8.2.9, Interim Analyses.

8.2.5.2 Statistical Methods for Safety Analyses

Safety and tolerability will be assessed by clinical review of all relevant parameters including adverse experiences (AEs), laboratory tests, and vital signs. Safety summaries will be pooled across all indications.

Immune related adverse experiences (irAEs, as defined the Event of Clinical Interest Guidance Document located in the Administrative Binder) are prespecified as events of interest. These events will be summarized in separate tables from other AEs by toxicity grade and will include the counts, percentage, and 95% CI. Any AE of unknown etiology associated with pembrolizumab (MK-3475) exposure will be evaluated to determine if it is

possibly an event of clinical interest (ECI) of a potentially immunologic etiology (irECI). Other ECIs listed in section 7.2.3.2 will also be summarized in the same manner as irAEs.

Adverse experiences (specific terms as well as system organ class terms) and predefined limits of change in laboratory, and vital sign parameters that are not pre-specified as events of interest will be summarized with descriptive statistics (counts, percentage, mean, standard deviation, etc.).

Continuous measures such as changes from baseline in laboratory, and vital signs parameters that are not pre-specified as events of interest will be summarized using descriptive statistics (mean, standard deviation, etc.) for baseline, on-treatment, and change from baseline values.

8.2.5.3 Summaries of Baseline Characteristics, Demographics, and Other Analyses

8.2.5.3.1 Demographic and Baseline Characteristics

Baseline characteristics will be assessed by the use of tables and/or graphs for each indication. No statistical hypothesis tests will be performed on these characteristics. The number and percentage of subjects screened, allocated, the primary reasons for screening failure, and the primary reason for discontinuation will be displayed. Demographic variables (e.g., age, gender), baseline characteristics, primary and secondary diagnoses, and prior and concomitant therapies will be summarized by treatment either by descriptive statistics or categorical tables.

8.2.6 Multiplicity

The false positive rate for testing the primary efficacy endpoint in each disease indication is controlled at 0.08 (1-sided) for each cohort. No additional multiplicity adjustment is required because each disease indication will be evaluated independently.

8.2.7 Sample Size and Power Calculations

Efficacy

Within each indication, the study will enroll a minimum of 6 subjects. Following the time that at least 6 subjects have had at least one post-baseline response assessment, a sequential monitoring procedure will be used to evaluate for efficacy and futility simultaneously based on the number of subjects with a confirmed or unconfirmed response according to the rules outlined in Figure 2, Table 11, and Table 12.

Enrollment will not be suspended at 6 subjects for the initial evaluation of efficacy.

Once at least 6 subjects are evaluable for confirmed or unconfirmed response, subsequent rules for pausing enrollment and future evaluations will be based on the boundaries identified by the sequential monitoring procedure. If 0 out of the first 6 subjects with a post-baseline evaluation have a confirmed or unconfirmed response following the first post-baseline assessment, then enrollment will be paused in that indication. The first 6 subjects and any additional subjects enrolled up to the time that the first 6 subjects are evaluated will continue to be followed for response. If the required number of subjects subsequently develop a confirmed or unconfirmed response to continue enrollment according to Figure 2, Table 11,

and Table 12, then enrollment may resume in that indication. If ≥1 of the first 6 subjects with a post-baseline evaluation have a confirmed or unconfirmed response, then enrollment will continue. Depending on the enrollment rate, additional monitoring may be performed according to the boundaries defined in Figure 2, Table 11, and Table 12 until a maximum of approximately 22 subjects is enrolled within an indication.

If at any time during the trial, the efficacy boundary is crossed for a given indication, enrollment will continue to 22 subjects and subsequent trial planning may be initiated.

If enrollment within an indication is so rapid that 22 subjects are assigned a screening slot for potential enrollment at the time that the first 6 subjects are evaluable for response, then no sequential monitoring will be performed. If enrollment within an indication is so slow that a cohort is unlikely to complete within a reasonable amount of time, a cohort may be closed (e.g. low prevalence of PD-L1 such that fewer than 3 PD-L1 positive subjects are enrolled within a cohort after approximately 6 months of screening activity from all sites).

With an approximate maximum of 22 subjects enrolled within each indication, the study provides 80% power to demonstrate that the best overall response rate induced by pembrolizumab (MK-3475) exceeds 10% at an overall one-sided 8% alpha-level, if the true best overall response rate is 35%. The null hypothesis of 10% is based on the assumption that the population for each indication is expected to consist of subjects with incurable solid tumors that have failed multiple lines of standard therapy. The best ORR for the limited treatment options available in these subject populations is generally <10%. The alternative best overall response rate is determined to be a clinically meaningful improvement over other standard treatment options within each studied indication. The calculation is based on the binomialSPRT function in the gsDesign package and is carried out using R assuming a null best ORR of 10%, an alternative best ORR of 35%, type I error of 0.08 and type II error of 0.2 (binomialSPRT (p0=0.1, p1=0.35, alpha=0.08, beta=0.2, minn=6, maxn=22)).

For the final analysis, the response assessment will be based on confirmed best overall response rate. The adjusted p-value and repeated confidence interval will be computed using sequential methods outlined by Jennison and Turnbull (2000) [33]. The minimum criterion for success is that the lower bound of the adjusted CI > 10%. Given the underlying true rate, this may occur when at least 6/22 subjects develop a confirmed PR or CR. Table 15 summarizes the power under various assumptions.

If the optional exploratory efficacy objective of best overall response rate per RECIST 1.1 based on central radiology review, best overall response rate per RANO criteria (GBM cohort), or best overall response rate per PCWG2 (prostate cancer cohort) is evaluated in an indication, and if fewer than 22 subjects in the respective indication have centrally confirmed measurable disease at baseline in that indication, then additional subjects may be enrolled to ensure that at least 22 subjects with centrally confirmed measurable disease are evaluable for the applicable central radiologist response assessment.

Table 15 Operating Characteristics of the Sequential Monitoring Approach

True RR	Probability of Stopping for Futility	Probability of Go at end of Study	Average Sample Size
10%	0.92	0.05	9.9
15%	0.76	0.15	12.6
20%	0.57	0.31	15.2
25%	0.39	0.50	17.3
30%	0.25	0.67	18.9
35%	0.14	0.80	20.1
40%	0.08	0.89	20.9
45%	0.04	0.95	21.4
50%	0.02	0.97	21.7

Because the maximum sample size within each indication is 22 subjects (assuming no additional subjects needed for the optional exploratory objective evaluation) the maximum sample size for the entire study is approximately 440 subjects. However, the total enrollment is estimated as ~320 subjects, as it is expected that 10 indications will enroll to the full 22 subjects (22*10=220 subjects), and that the other 10 indications will be stopped for futility with, on average, 10 subjects enrolled (10*10=100 subjects).

Safety

The probability of observing at least 10 subjects with a Grade 3-5 immune related in this study depends on the number of subjects treated and the underlying percentage of subjects with irAEs in the study population. If the underlying incidence of Grade 3-5 irAE is 10%, there is a 77% chance of observing at least 10 subjects with irAEs among the minimum 120 subjects enrolled in the study across all indications, whereas if the underlying incidence is 5% there is a 7.8% chance of observing at 10 occurrences or irAEs. Table 16 provides the point estimate and 95% CI for the underlying percentage of subjects with irAEs given various hypothetical numbers of subjects with irAEs and sample size across indications.

Table 16 Point Estimate and 95% CI for Hypothetical Number of Subjects with a Grade 3-5 irAE

Number of Subjects with Grade 3-5 irAE	Number of Subjects	Point Estimate	95% CI†
10	120	8.3%	(4.1%, 14.8%)
15	120	12.5%	(7.2%, 19.8%)
20	120	16.7%	(10.5%, 24.6%)
10	220	4.5%	(2.2%, 8.2%)
15	220	6.8%	(3.9%, 11.0%)
20	220	9.1%	(5.6%, 13.7%)
10	320	3.1%	(1.5%, 5.7%)
15	320	4.7%	(2.6%, 7.6%)
20	320	6.3%	(3.9%, 9.5%)
10	440	2.3%	(1.1%, 4.1%)
15	440	3.4%	(1.9%, 5.6%)
20	440	4.5%	(2.8%, 6.9%)

† Based on the two-tailed exact confidence interval of a binomial proportion (Clopper and Pearson, 1934) [34].

8.2.8 Subgroup Analyses and Effect of Baseline Factors

No subgroup analysis is planned.

8.2.9 Interim Analyses

Multiple interim analyses may be performed in this study due to the sequential design of the trial. Results will be reviewed by the study team. The primary best overall response rate endpoint will be used for all interim decision-making with or without a confirmation assessment; however, a confirmation assessment is required for the final best overall response rate analyses after 22 subjects are enrolled in a cohort. For purpose of interim monitoring, subjects who are still on study but without a post baseline scan will be excluded from the analyses specified in Table 14. The decision rule and other statistical details are further described in Section 8.2.7.

8.2.10 Compliance (Medication Adherence)

A day within the study will be considered an On-Therapy day if the subject receives the study medication infusion. The number of Days Should be on Therapy is the total number of days from the first day of study medication to the date of the last dose of study medication. For each subject, percent compliance will then be calculated using the following formula:

$$\text{Percent Compliance} = \frac{\text{Number of Days on Therapy}}{\text{Number of Days Should be on Therapy}} \text{ x } 100$$

Summary statistics will be provided on percent compliance by treatment group for the FAS population.

8.2.11 Extent of Exposure

Extent of Exposure for a subject is defined as number of cycles in which the subject receives the study medication infusion. Summary statistics will be provided on Extent of Exposure for APaT population.

9.0 LABELING, PACKAGING, STORAGE AND RETURN OF CLINICAL SUPPLIES

9.1 Investigational Product

The investigator shall take responsibility for and shall take all steps to maintain appropriate records and ensure appropriate supply, storage, handling, distribution and usage of investigational product in accordance with the protocol and any applicable laws and regulations.

Clinical Supplies will be provided by the Sponsor as summarized in Table 17.

Table 17 Product Descriptions

Product Name & Potency	Dosage Form
pembrolizumab (MK-3475) 100 mg/4 mL	Solution for Infusion

9.2 Packaging and Labeling Information

Clinical supplies will be affixed with a clinical label in accordance with regulatory requirements.

Vials will be provided in an open label fashion for subject dosing.

9.3 Clinical Supplies Disclosure

This trial is open-label; therefore, the subject, the trial site personnel, the Sponsor and/or designee are not blinded. Treatment (name, strength or potency) is included in the label text; random code/disclosure envelopes or lists are not provided.

9.4 Storage and Handling Requirements

Clinical supplies must be stored in a secure, limited-access location under the storage conditions specified on the label.

Receipt and dispensing of trial medication must be recorded by an authorized person at the trial site.

Clinical supplies may not be used for any purpose other than that stated in the protocol.

9.5 Returns and Reconciliation

The investigator is responsible for keeping accurate records of the clinical supplies received from the Sponsor or designee, the amount dispensed to and returned by the subjects and the amount remaining at the conclusion of the trial.

For all trial sites, the local country Sponsor personnel or designee will provide appropriate documentation that must be completed for drug accountability and return.

9.6 Standard Policies

Trial site personnel will have access to a central electronic randomization system (IVRS/IWRS system) to allocate subjects, to assign treatment to subjects and to manage the distribution of clinical supplies. Each person accessing the IVRS system must be assigned an individual unique PIN. They must use only their assigned PIN to access the system, and they must not share their assigned PIN with anyone.

10.0 ADMINISTRATIVE AND REGULATORY DETAILS

10.1 Confidentiality

10.1.1 Confidentiality of Data

By signing this protocol, the investigator affirms to the Sponsor that information furnished to the investigator by the Sponsor will be maintained in confidence, and such information will be divulged to the institutional review board, ethics review committee (IRB/ERC) or similar or expert committee; affiliated institution and employees, only under an appropriate understanding of confidentiality with such board or committee, affiliated institution and employees. Data generated by this trial will be considered confidential by the investigator, except to the extent that it is included in a publication as provided in the Publications section of this protocol.

10.1.2 Confidentiality of Subject Records

By signing this protocol, the investigator agrees that the Sponsor (or Sponsor representative), IRB/ERC, or regulatory authority representatives may consult and/or copy trial documents in order to verify worksheet/case report form data. By signing the consent form, the subject agrees to this process. If trial documents will be photocopied during the process of verifying worksheet/case report form information, the subject will be identified by unique code only; full names/initials will be masked prior to transmission to the Sponsor.

By signing this protocol, the investigator agrees to treat all subject data used and disclosed in connection with this trial in accordance with all applicable privacy laws, rules and regulations.

10.1.3 Confidentiality of Investigator Information

By signing this protocol, the investigator recognizes that certain personal identifying information with respect to the investigator, and all subinvestigators and trial site personnel, may be used and disclosed for trial management purposes, as part of a regulatory submissions, and as required by law. This information may include:

1. name, address, telephone number and e-mail address;
2. hospital or clinic address and telephone number;
3. curriculum vitae or other summary of qualifications and credentials; and
4. other professional documentation.

Consistent with the purposes described above, this information may be transmitted to the Sponsor, and subsidiaries, affiliates and agents of the Sponsor, in your country and other countries, including countries that do not have laws protecting such information. Additionally, the investigator's name and business contact information may be included when reporting certain serious adverse events to regulatory authorities or to other investigators. By signing this protocol, the investigator expressly consents to these uses and disclosures.

If this is a multicenter trial, in order to facilitate contact between investigators, the Sponsor may share an investigator's name and contact information with other participating investigators upon request.

10.1.4 Confidentiality of IRB/IEC Information

The Sponsor is required to record the name and address of each IRB/IEC member that reviews and approves this trial. The Sponsor is also required to document that each IRB/IEC meets regulatory and ICH GCP requirements by requesting and maintaining records of the names and qualifications of the IRB/IEC members and to make these records available for regulatory agency review upon request by those agencies.

10.2 Compliance with Financial Disclosure Requirements

Financial Disclosure requirements are outlined in the US Food and Drug Administration Regulations, Financial Disclosure by Clinical Investigators (21 CFR Part 54). It is the Sponsor's responsibility to determine, based on these regulations, whether a request for Financial Disclosure information is required. It is the investigator's/subinvestigator's responsibility to comply with any such request.

The investigator/subinvestigator(s) agree, if requested by the Sponsor in accordance with 21 CFR Part 54, to provide his/her financial interests in and/or arrangements with the Sponsor to allow for the submission of complete and accurate certification and disclosure statements. The investigator/subinvestigator(s) further agree to provide this information on a

Certification/Disclosure Form, commonly known as a financial disclosure form, provided by the Sponsor or through a secure password-protected electronic portal provided by the Sponsor. The investigator/subinvestigator(s) also consent to the transmission of this information to the Sponsor in the United States for these purposes. This may involve the transmission of information to countries that do not have laws protecting personal data.

10.3 Compliance with Law, Audit and Debarment

By signing this protocol, the investigator agrees to conduct the trial in an efficient and diligent manner and in conformance with this protocol; generally accepted standards of Good Clinical Practice (e.g., International Conference on Harmonization of Technical Requirements for Registration of Pharmaceuticals for Human Use Good Clinical Practice: Consolidated Guideline and other generally accepted standards of good clinical practice); and all applicable federal, state and local laws, rules and regulations relating to the conduct of the clinical trial.

The Code of Conduct, a collection of goals and considerations that govern the ethical and scientific conduct of clinical investigations sponsored by Merck, is provided in Section 12.1 - Merck Code of Conduct for Clinical Trials.

The investigator also agrees to allow monitoring, audits, IRB/ERC review and regulatory authority inspection of trial-related documents and procedures and provide for direct access to all trial-related source data and documents.

The investigator agrees not to seek reimbursement from subjects, their insurance providers or from government programs for procedures included as part of the trial reimbursed to the investigator by the Sponsor.

The investigator shall prepare and maintain complete and accurate trial documentation in compliance with Good Clinical Practice standards and applicable federal, state and local laws, rules and regulations; and, for each subject participating in the trial, provide all data, and, upon completion or termination of the clinical trial, submit any other reports to the Sponsor as required by this protocol or as otherwise required pursuant to any agreement with the Sponsor.

Trial documentation will be promptly and fully disclosed to the Sponsor by the investigator upon request and also shall be made available at the trial site upon request for inspection, copying, review and audit at reasonable times by representatives of the Sponsor or any regulatory authorities. The investigator agrees to promptly take any reasonable steps that are requested by the Sponsor as a result of an audit to cure deficiencies in the trial documentation and worksheets/case report forms.

The investigator must maintain copies of all documentation and records relating to the conduct of the trial in compliance with all applicable legal and regulatory requirements. This documentation includes, but is not limited to, the protocol, worksheets/case report forms, advertising for subject participation, adverse event reports, subject source data,

correspondence with regulatory authorities and IRBs/ERCs, consent forms, investigator's curricula vitae, monitor visit logs, laboratory reference ranges, laboratory certification or quality control procedures and laboratory director curriculum vitae. By signing this protocol, the investigator agrees that documentation shall be retained until at least 2 years after the last approval of a marketing application in an ICH region or until there are no pending or contemplated marketing applications in an ICH region or until at least 2 years have elapsed since the formal discontinuation of clinical development of the investigational product. Because the clinical development and marketing application process is variable, it is anticipated that the retention period can be up to 15 years or longer after protocol database lock. The Sponsor will determine the minimum retention period and notify the investigator when documents may be destroyed. The sponsor also recognizes that documents may need to be retained for a longer period if required by local regulatory requirements. All trial documents shall be made available if required by relevant regulatory authorities. The investigator must consult with and obtain written approval by the Sponsor prior to discarding trial and/or subject files.

ICH Good Clinical Practice guidelines recommend that the investigator inform the subject's primary physician about the subject's participation in the trial if the subject has a primary physician and if the subject agrees to the primary physician being informed.

The investigator will promptly inform the Sponsor of any regulatory authority inspection conducted for this trial.

Persons debarred from conducting or working on clinical trials by any court or regulatory authority will not be allowed to conduct or work on this Sponsor's trials. The investigator will immediately disclose in writing to the Sponsor if any person who is involved in conducting the trial is debarred or if any proceeding for debarment is pending or, to the best of the investigator's knowledge, threatened.

In the event the Sponsor prematurely terminates a particular trial site, the Sponsor will promptly notify that trial site's IRB/IEC.

According to European legislation, a Sponsor must designate an overall coordinating investigator for a multi-center trial (including multinational). When more than one trial site is open in an EU country, Merck, as the Sponsor, will designate, per country, a national principal coordinator (Protocol CI), responsible for coordinating the work of the principal investigators at the different trial sites in that Member State, according to national regulations. For a single-center trial, the Protocol CI is the principal investigator. In addition, the Sponsor must designate a principal or coordinating investigator to review the trial report that summarizes the trial results and confirm that, to the best of his/her knowledge, the report accurately describes the conduct and results of the trial [Clinical Study Report (CSR) CI]. The Sponsor may consider one or more factors in the selection of the individual to serve as the Protocol CI and or CSR CI (e.g., availability of the CI during the anticipated review process, thorough understanding of clinical trial methods, appropriate enrollment of subject cohort, timely achievement of trial milestones). The Protocol CI must be a participating trial investigator.

10.4 Compliance with Trial Registration and Results Posting Requirements

Under the terms of the Food and Drug Administration Modernization Act (FDAMA) and the Food and Drug Administration Amendments Act (FDAAA), the Sponsor of the trial is solely responsible for determining whether the trial and its results are subject to the requirements for submission to the Clinical Trials Data Bank, http://www.clinicaltrials.gov. Merck, as Sponsor of this trial, will review this protocol and submit the information necessary to fulfill these requirements. Merck entries are not limited to FDAMA/FDAAA mandated trials. Information posted will allow subjects to identify potentially appropriate trials for their disease conditions and pursue participation by calling a central contact number for further information on appropriate trial locations and trial site contact information.

By signing this protocol, the investigator acknowledges that the statutory obligations under FDAMA/FDAAA are that of the Sponsor and agrees not to submit any information about this trial or its results to the Clinical Trials Data Bank.

10.5 Quality Management System

By signing this protocol, the Sponsor agrees to be responsible for implementing and maintaining a quality management system with written development procedures and functional area standard operating procedures (SOPs) to ensure that trials are conducted and data are generated, documented, and reported in compliance with the protocol, accepted standards of Good Clinical Practice, and all applicable federal, state, and local laws, rules and regulations relating to the conduct of the clinical trial.

10.6 Data Management

The investigator or qualified designee is responsible for recording and verifying the accuracy of subject data. By signing this protocol, the investigator acknowledges that his/her electronic signature is the legally binding equivalent of a written signature. By entering his/her electronic signature, the investigator confirms that all recorded data have been verified as accurate.

Detailed information regarding Data Management procedures for this protocol will be provided separately.

10.7 Publications

This trial is intended for publication, even if terminated prematurely. Publication may include any or all of the following: posting of a synopsis online, abstract and/or presentation at a scientific conference, or publication of a full manuscript. The Sponsor will work with the authors to submit a manuscript describing trial results within 12 months after the last data become available, which may take up to several months after the last subject visit in some cases such as vaccine trials. However, manuscript submission timelines may be extended on OTC trials. For trials intended for pediatric-related regulatory filings, the investigator agrees to delay publication of the trial results until the Sponsor notifies the investigator that all relevant regulatory authority decisions on the trial drug have been made with regard to

pediatric-related regulatory filings. Merck will post a synopsis of trial results for approved products on www.clinicaltrials.gov by 12 months after the last subject's last visit for the primary outcome, 12 months after the decision to discontinue development, or product marketing (dispensed, administered, delivered or promoted), whichever is later.

These timelines may be extended for products that are not yet marketed, if additional time is needed for analysis, to protect intellectual property, or to comply with confidentiality agreements with other parties. Authors of the primary results manuscript will be provided the complete results from the Clinical Study Report, subject to the confidentiality agreement. When a manuscript is submitted to a biomedical journal, the Sponsor's policy is to also include the protocol and statistical analysis plan to facilitate the peer and editorial review of the manuscript. If the manuscript is subsequently accepted for publication, the Sponsor will allow the journal, if it so desires, to post on its website the key sections of the protocol that are relevant to evaluating the trial, specifically those sections describing the trial objectives and hypotheses, the subject inclusion and exclusion criteria, the trial design and procedures, the efficacy and safety measures, the statistical analysis plan, and any amendments relating to those sections. The Sponsor reserves the right to redact proprietary information.

For multicenter trials, subsequent to the multicenter publication (or after public disclosure of the results online at www.clinicaltrials.gov if a multicenter manuscript is not planned), an investigator and his/her colleagues may publish their data independently. In most cases, publication of individual trial site data does not add value to complete multicenter results, due to statistical concerns. In rare cases, publication of single trial site data prior to the main paper may be of value. Limitations of single trial site observations in a multicenter trial should always be described in such a manuscript.

Authorship credit should be based on 1) substantial contributions to conception and design, or acquisition of data, or analysis and interpretation of data; 2) drafting the article or revising it critically for important intellectual content; and 3) final approval of the version to be published. Authors must meet conditions 1, 2 and 3. Significant contributions to trial execution may also be taken into account to determine authorship, provided that contributions have also been made to all three of the preceding authorship criteria. Although publication planning may begin before conducting the trial, final decisions on authorship and the order of authors' names will be made based on participation and actual contributions to the trial and writing, as discussed above. The first author is responsible for defending the integrity of the data, method(s) of data analysis and the scientific content of the manuscript.

The Sponsor must have the opportunity to review all proposed abstracts, manuscripts or presentations regarding this trial 45 days prior to submission for publication/presentation. Any information identified by the Sponsor as confidential must be deleted prior to submission; this confidentiality does not include efficacy and safety results. Sponsor review can be expedited to meet publication timelines.

11.0 LIST OF REFERENCES

[1] Disis ML. Immune regulation of cancer. J Clin Oncol 2010;28(29):4531-8.

[2] Dong H, Strome SE, Salomao DR, Tamura H, Hirano F, Flies DB, et al. Tumor-associated B7-H1 promotes T-cell apoptosis: a potential mechanism of immune evasion. Nat Med 2002;8(8):793-800.

[3] Sharpe AH, Freeman GJ. The B7-CD28 superfamily. Nature 2002;2:116-26.

[4] Brown JA, Dorfman DM, Ma F-R, Sullivan EL, Munoz O, Wood CR, et al. Blockade of programmed death-1 ligands on dendritic cells enhances T cell activation and cytokine production. J Immunol 2003;170:1257-66.

[5] Francisco LM, Sage PT, Sharpe AH. The PD-1 pathway in tolerance and autoimmunity. Immunol Rev 2010;236:219-42.

[6] Thompson RH, Dong H, Lohse CM, Leibovich BC, Blute ML, Cheville JC, et al. PD-1 expressed by tumor-infiltrating immune cells and is associated with poor outcome for patients with renal cell carcinoma. Clin Caner Res 2007;13(6):1757-61.

[7] Talmadge JE, Donkor M, Scholar E. Inflammatory cell infiltration of tumors: Jekyll or Hyde. Cancer Metastasis Rev 2007;26:373-400.

[8] Usubütün A, Ayhan A, Uygur MC, Özen H, Toklu C, Ruacan S. Prognostic factors in renal cell carcinoma. J Exp Clin Cancer Res 1998;17(1):77-81.

[9] Al-Shibli KI, Donnem T, Al-Saad S, Persson M, Bremnes RM, Busund L-T. Prognostic effect of epithelial and stromal symphocyte infiltration in non-small cell lung cancer. Clin Cancer Res 2008;14(16):5220-7.

[10] Deschoolmeester V, Baay M, Van Marck E, Weyler J, Vermeulen p, Lardon F, et al. Tumor infiltrating lymphocytes: an intriguing player in the survival of colorectal cancer patients. BMC Immunol 2010;11:19.

[11] Diez M, Pollán M, Enriquez JM, Dominguez P, Santana A, Tobaruela E, et al. Histopathologic prognostic score in colorectal adenocarcinomas. Anticancer Res 1998;18:689-94.

[12] Galon J, Costes A, Sanchez-Cabo F, Kirilovsky A, Mlecnik B, Lagorce-Pagès C, et al. Type, density, and location of immune cells within human colorectal tumors predict clinical outcome. Science 2006;313:1960-4.

[13] Hiraoka N. Tumor-infiltrating lymphocytes and hepatocellular carcinoma: molecular biology. Int J Clin Oncol 2010;15:544-51.

[14] Nobili C, Degrate L, Caprotti R, Franciosi C, Leone BE, Trezzi R, et al. Prolonged survival of a patient affected by pancreatic adenocarcinoma with massive lymphocyte and dendritic cell infiltration after interleukin-2 immunotherapy. Report of a case. Tumori 2008;94:426-30.

[15] Hodi FS, Dranoff G. The biologic importance of tumor-infiltrating lymphocytes. J Cutan Pathol 2010;37(Suppl 1):48-53.

[16] Kloor M. Lymphocyte infiltration and prognosis in colorectal cancer. Lancet 2009;10(840):841.

[17] Hillen F, Baeten CIM, van de Winkel A, Creytens D, van der Schaft DWJ, Winnepenninckx V, et al. Leukocyte infiltration and tumor cell plasticity are parameters of aggressiveness in primary cutaneous melanoma. Cancer Immunol Immunother 2008;57:97-106.

[18] Lee HE, Chae SW, Lee YJ, Kim MA, Lee HS, Lee BL, et al. Prognostic implications of type and density of tumour-infiltrating lymphocytes in gastric cancer. Br J Cancer 2008;99(10):1704-11.

[19] Leffers N, Gooden MJM, de Jong RA, Hoogeboom B-N, ten Hoor KA, Hollema H, et al. Prognostic significance of tumor-infiltrating T-lymphocytes in primary and metastatic lesions of advanced stage ovarian cancer. Cancer Immunol Immunother 2009;58:449-59.

[20] Nishimura H, Honjo T, Minato N. Facilitation of ßselection and modification of positive selection in the thymus of PD-1-deficient mice. J Exp Med 2000;191(5):891-7.

[21] Liotta F, Gacci M, Frosali F, Querci V, Vittori G, Lapini A, et al. Frequency of regulatory T cells in peripheral blood and in tumour-infiltrating lymphocytes correlates with poor prognosis in renal cell carcinoma. BJU Intern 2010;107:1500-6.

[22] Ropponen KM, Eskelinen MJ, Lipponen PK, Alhava E, Kosma V-M. Prognostic value of tumour-infiltrating lynmphocytes (TILs) in colorectal cancer. J Pathol 1997;182:318-24.

[23] Dudley ME, Wunderlich JR, Yang JC, Sherry RM, Topalian SL, Restifo NP, et al. Adoptive cell transfer therapy following non-myeloablative but lymphodepleting chemotherapy for the treatment of patients with refractory metastatic melanoma. J Clin Oncol 2005;23(10):2346-57.

[24] Hunder NN, Wallen H, Cao J, Hendricks DW, Reilly JZ, Rodmyre R, et al. Treatment of metastatic melanoma with autologous CD4+ T cells against NY-ESO-1. N Engl J Med 2008;358(25):2698-703.

[25] Pölcher M, Braun M, Friedrichs N, Rudlowski C, Bercht E, Fimmers R, et al. Foxp3+ cell infiltration and granzyme B+ /Foxp3+ cell ratio are associated with outcome in neoadjuvant chemotherapy-treated ovarian carcinoma. Cancer Immunol Immunother 2010;59:909-19.

[26] Okazaki T, Maeda A, Nishimura H, Kurosaki T, Honjo T. PD-1 immunoreceptor inhibits B cell receptor-mediated signaling by recruiting src homology 2-domain-containing tyrosine phosphatase 2 to phosphotyrosine. Proc Natl Acad Sci U S A 2001;98(24):13866-71.

[27] Greenwald RJ, Freeman GJ, Sharpe AH. The B7 family revisted. Annu Rev Immunol 2005;23:515-48.

[28] Topalian SL, Hodi FS, Brahmer JR, Gettinger SN, Smith DC, McDermott DF, et al. Safety, activity, and immune correlates of Anti-PD-1 antibody in cancer. N Engl J Med. "in press" 2012.

[29] Eisenhauer EA, Therasse P, Bogaerts J, Schwartz LH, Sargent D, Ford R, et al. New response evaluation criteria in solid tumors: revised RECIST guideline (version 1.1). Eur J Cancer 2009;45:228-47.

[30] Wen PY, Macdonald DR, Reardon DA, Cloughesy TF, Sorensen AG, Galanis E., et al. Updated response assessment criteria for high-grade gliomas: Response assessment in neuro-oncology working group. J Clin Oncol 2010;28(11):1963-72.

[31] Scher HI, Halabi S, Tannock I, Morris M, Sternberg CN, Carducci MA, Eisenberger MA, et al. Design and end points of clinical trials for patients with progressive prostate cancer and castrate levels of testosterone: Recommendations of the prostate Cancer clinical trials working group. J Clin Oncol 2008;26(7):1148-59.

[32] Romeu, JL. Understanding Binomial Sequential Testing. Statistical Confidence, Reliability Information Analysis Center (RIAC START). Department of Defense United States of America, 2013.

[33] Jennison C, Turnbull BW. Analysis following a sequential test. In: Group Sequential Methods with Application to Clinical Trials. Boca Raton, FL: Chapman & Hall/CRC, 2000:171-81.

[34] Clopper CJ, Pearson ES. The use of confidence or Fiducial limits illustrated in the case of the binomial. Biometrika 1934;26(4):404-13.
